# A high dimensionality approach reveals immunopathogenic responses driving severe pediatric acute respiratory distress syndrome

**DOI:** 10.1038/s41467-026-73181-2

**Published:** 2026-05-15

**Authors:** Judith Ju Ming Wong, Herng Lee Tan, Clare Wei Tian Foo, Nicholas Kim Huat Khoo, Yik-Lam Cho, Su Li Poh, Martin Wasser, Sharifah Hazirah, Jing Yao Leong, Yee Hui Mok, Daryl Zhang Wei Lee, Pavanish Kumar, Joo Guan Yeo, Sylvie Alonso, Salvatore Albani

**Affiliations:** 1 Children’s Intensive Care Unit, Department of Pediatric Subspecialties, KK Women’s and Children’s Hospital, Singapore, Singapore; 2 Duke-NUS Medical School, Singapore, Singapore; 3 Translational Immunology Institute, SingHealth Duke-NUS Academic Medical Centre, Singapore, Singapore; 4 Respiratory Therapy Service, Division of Allied Health Specialties, KK Women’s and Children’s Hospital, Singapore, Singapore; 5 Division of Medicine, KK Women’s and Children’s Hospital, Singapore, Singapore; 6 Infectious Diseases Translational Research Programme, Department of Microbiology & Immunology, Yong Loo Lin School of Medicine, National University of Singapore, Singapore, Singapore; 7 Immunology Programme, Life Sciences Institute, National University of Singapore, Singapore, Singapore; 8 National Centre for Infectious Diseases, Singapore, Singapore

**Keywords:** Acute inflammation, Toll-like receptors, Respiratory distress syndrome, RNA sequencing

## Abstract

Mechanisms underlying paediatric acute respiratory distress syndrome (PARDS) remain poorly understood, limiting advances in diagnosis and treatment. To address this, we conduct a high-dimensional, multi-omics analysis of paired pulmonary and blood samples from children with PARDS and age-matched controls. Our approach includes transcriptomics, proteomics, flow and mass cytometry, and single-cell RNA sequencing, with further validation using cytokine assays and in vitro models. Severe PARDS is characterised by three convergent immune abnormalities; Pulmonary CD8 ^+^ T cells display an interferon-driven cytotoxic profile, with exhaustion and apoptosis genes; Pulmonary T cells and myeloid cells exhibit strong interferon-stimulated gene expression; Distinct macrophage subsets show high interferon but suppressed IL-1 pathway genes, associated with impaired leukocyte chemotaxis, phagocytosis, and M1-polarization. This is mirrored by reduced pulmonary IL-1α/β and elevated IFN-γ. The systemic IL-1 signature is similarly dampened, while interferon responses are compartmentalised to the lung. Using an in vitro model, IFN-γ priming is shown to suppress TLR7-induced IL-1 β production through transcriptional inhibition of downstream inflammatory pathways, recapitulating the immune signature observed in patients. Our results reveal interferon-driven immune dysregulation and IL-1 suppression as central features of severe PARDS, highlighting parallels and differences from adult ARDS and underscoring the need for paediatric-specific therapeutic strategies.

## Abstract (web summary)

Paediatric acute respiratory distress syndrome (ARDS) is a potentially life-threatening condition in children, understanding the immuno-pathology is of importance. Here the authors employ a high-dimensionality approach to identify dysregulated immune pathways underpinning the condition, and find important differences compared to ARDS in adults.

## Introduction

Pediatric acute respiratory distress syndrome (PARDS) is characterized by persistent inflammation with increased permeability in the lung, manifesting as severe oxygenation failure, and associated with high morbidity and mortality^1^. As a consequence of a transient inciting agent (e.g., pneumonia, aspiration, ischemia-reperfusion, transfusion), PARDS is characterized by persistent inflammation with increased permeability in the lung beyond the duration of the trigger^2^. Indeed, PARDS may progressively worsen even after elimination of the trigger, indicative of an exuberant/ dysregulated immune response^3^. The pathogenesis of ARDS, studied mainly in adults, involves a dysregulated immune response. Evidence for an immune-mediated mechanism of lung injury has implicated deployment of neutrophil extracellular traps (NET)^4^, sustained influx of CD14^+^CD11b^+^ monocytes into alveolar spaces^5^, inflammatory Th17 responses^6^ and deficiency of CD4^+^CD25^+^FOXP3^+^ T regulatory cells^7^ as maladaptive processes which contribute to lung injury. However, PARDS is distinct from adult ARDS in its epidemiology^8,9^, mechanical and physiological properties of the lung^10^, ultrastructure^11^, and the stage of immune maturation^12–14^. Therefore, the immunopathogenic mechanism(s) in PARDS are likely distinct from adult ARDS^8,15^.

Currently, PARDS is diagnosed by the Pediatric Acute Lung Injury Consensus Conference (PALICC) clinical criteria^16^. Some patients diagnosed with PARDS were later found to have rapidly reversible hypoxemia (within 24–48 hours). It is postulated that they may not display the pathological changes or immune-mediated injury originally described which generally progress over the first week of illness through the exudative, proliferative and fibrotic stages^17^. And yet, there are also patients who unpredictably progress from non-severe to severe PARDS with its associated poorer outcomes. This highlights the limitation of clinical diagnostic criteria. In addition, there is a lack of clinical or laboratory indicators which can reliably discriminate whether a patient diagnosed with PARDS will resolve quickly or progress to severe disease. There is no method to identify which patient at-risk of PARDS, may develop PARDS. One reason for this unmet clinical need is that there are limited studies evaluating the pathogenesis in PARDS. While most studies focus on single, or several predetermined markers based on adult literature^18^, it has been shown that there are age-dependent differences in biomarker concentration, which are more prominent than disease-dependent changes supporting the notion that the host immune response to triggering agents are different compared to adults and that data from adult studies may not be extrapolated to children^19^. Blood biomarker panels derived from adult data including inflammatory cytokines, chemokines, and tissue injury markers (IL-6, IL-8, sTNFR1, CCL3, MMP8, ANG2) have been used as tools to predict sub-phenotypes in PARDS (hypoinflammatory and hyperinflammatory phenotypes) which are associated with distinct mortality risks^20,21^. However, none of these single or panel-based biomarker studies shed insight into the pathogenesis of PARDS.

As PARDS differs from adult ARDS in immune development, epidemiology, and lung physiology, adult-derived biomarkers and pathogenesis studies do not fully extrapolate to children. To address this gap, we adopt a high-dimensional multi-omics approach to better understand immune dysregulation in PARDS, aiming to identify distinct pathogenic pathways. Downstream experiments validate the pathways identified, ensuring a comprehensive understanding of paediatric-specific mechanisms. We demonstrate that severe PARDS is marked by interferon-driven immune dysregulation, with pulmonary T cells and myeloid cells exhibiting strong interferon responses and suppressed IL-1 pathways, resulting in impaired immune cell function and reduced IL-1α/β levels. These findings distinguish severe PARDS from adult ARDS and emphasise the importance of developing pediatric-specific treatments.

## Results

### Patient cohort

Out of the cohort of patients recruited, 84/156 (53.8%) consented to biological sampling. After excluding patients with known immunosuppression, 69/84 (82.1%) samples were available for analysis. The median (interquartile range) age was 2.7 (0.5, 9.0) years, 38/70 (54.3%) were males and 41/70 (58.6%) had pre-existing comorbidities (Table 1). The majority of cases had direct PARDS 61/69 (88.4%) and of these, a viral pathogen was identified in 37/69 (53%). The median oxygenation index, a clinical marker of severity for hypoxic respiratory failure, was 9.5 (6.7, 15.0).

**Table 1:** Clinical characteristics of patients with PARDS

Clinical characteristics	Total, *n* = 69
Age, years	3.4 (0.6, 10.5)
Weight, kg	13.7 (6.4, 30.7)
Male	37 (53.6)
Comorbidities	40 (58.0)
Extrapulmonary PARDS	14 (21.9)
PIM 3 score, %	4.0 (1.7, 7.1)
PELOD 2 score	6 (3, 9)
Oxygenation index	10.5 (7.1, 15.0)
PARDS severity Mild Moderate Severe	19 (27.5) 25 (36.2) 25 (36.2)
Pathogen isolated^a^	
Virus	37 (53.6)
Bacteria	25 (36.2)
Fungal	5 (7.3)
None	13 (18.8)
Ventilator duration, days	7 (3, 12)
ICU length of stay, days	12 (7, 20)
ICU mortality	9 (13.0)

^a^Some patients had mixed infections and the total exceeds 69 patients.

### Pulmonary CD3^+^ T cells exhibited an IFN-γ response gene signature with increased expression of cytolytic, exhaustion/inhibition and apoptosis genes in severe PARDS

**Fig. 1:**
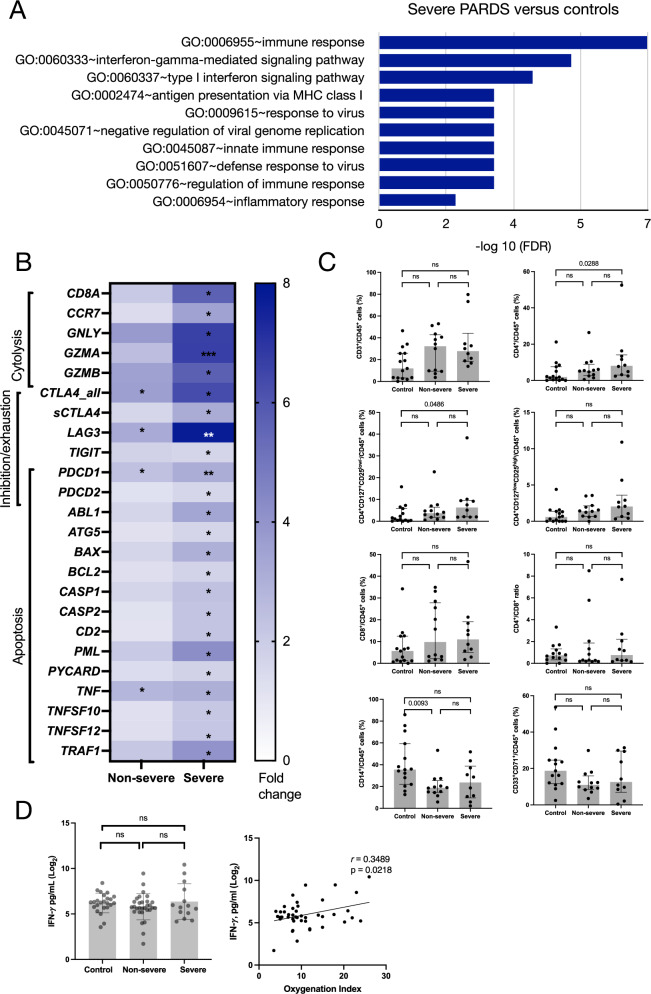
Derangements in the immune response of CD3^+^ T cells from the lung microenvironment of patients with PARDS. Immune cells from deep tracheal lavage samples were sorted using fluorescence-activated cell sorting for CD3^+^ T cells (PARDS at diagnosis, *n* = 22 and controls, *n* = 15). T cell subsets were then quantified using flow cytometry and their transcriptomic gene expression profiled using *NanoString* (PARDS at diagnosis, *n* = 18 and controls, *n* = 11). IFN-γ concentration within the deep tracheal lavage fluid was quantified using ELISA and dilution was normalized using the urea correction method (PARDS at diagnosis/ progression, *n* = 40 and controls, *n* = 24). **A** Top 10 upregulated gene ontology pathways enriched in CD3^+^ T cells of severe PARDS vs. control. **B** Heatmap of cytolytic, inhibition/ exhaustion and apoptosis genes in severe and non-severe PARDS vs. control. Two-tailed *t*-test was used without correction for multiple testing (*p < 0.05, **p < 0.01, ***p < 0.001). **C** Percentage of T cell subsets of total CD45^+^ immune cells in the lung microenvironment (median, interquartile range). A two-tailed Mann-Whitney U test was used for two-group comparisons of cell subset frequency between severe PARDS vs. control, non-severe PARDS vs control and non-severe vs severe PARDS. Each data point refers to an independent patient/control sample.** D** IFN-γ concentration in the deep tracheal lavage fluid in each comparison group (mean, standard deviation) and its correlation (Pearson’s, *r*) with the oxygenation index. ELISA was performed in duplicates, with the average presented; each data point refers to an independent patient/control sample. Two-tailed Student’s *t*-test was used for two group comparisons of IFN-γ concentration between severe PARDS vs. control, non-severe PARDS vs. control and non-severe vs. severe PARDS. Only significant *p* values after Bonferroni correction were reported (non-significant, ns).

Pulmonary CD3^+^ T-cells and CD14^+^ monocyte/macrophages from deep tracheal lavage (DTL) were fluorescence activated cell sorted (FACS) for probe-based transcriptomic analysis with *NanoString*. The most upregulated genes in severe PARDS were related to interferon-stimulation (*MX1*, *IFI16*, *IRF7*, *IFITM1*, *CCL4*) (Table S4). Gene set enrichment analysis of differentially upregulated lymphocyte genes in severe PARDS compared to control were involved in biological processes for immune response against insults (external and internal), downstream signaling after ligation of IFN-γ and type I IFN with their cognate receptors (Fig. 1A). Specifically, genes relating to cytolysis (*CD8A*, *CCR7*, *GNLY*, *GZMA*, *GZMB*), cellular exhaustion and immune-checkpoints (*CTLA4*, *PDCD1*, *LAG3*, *TIGIT*) and apoptosis (*BCL2*) genes were significantly upregulated in severe PARDS (Fig. 1B).

Concurrently, we examined the pulmonary immune cell composition by FACS to determine the T-cell subsets contributing to the CD3^+^ transcriptome. There was a significant increase of all pulmonary T-cell subsets in patients with PARDS as compared to age-matched controls (Fig. S1) though this influx of T-cell subsets was not significantly associated with disease severity (Fig. 1C). The T-cell changes involved an increase in the CD4^+^ T-cells and T-effector (CD4^+^CD127^+^CD25^low/-^) cells in severe PARDS compared to control, but not CD8^+^ T cells in PARDS samples. This finding correlated with the increased expression of exhaustion and apoptotic markers in CD8^+^ T cells observed by transcriptomics, possibly indicating qualitative (transcriptomic) and quantitative (cell frequencies) derangement. Consistent with the CD3^+^ T cell IFN-γ responsive gene signature, IFN-γ levels measured in the DTL samples were positively correlated to the severity of PARDS based on the oxygenation index (Pearson correlation coefficient, *r*_p_ = 0.3489, *p* = 0.0218.) (Fig. 1D).

### Pulmonary CD14^+^ myeloid cells exhibited a dampened immune response, impacting chemotaxis and migration, phagocytosis and M1 polarization in severe PARDS

**Fig. 2:**
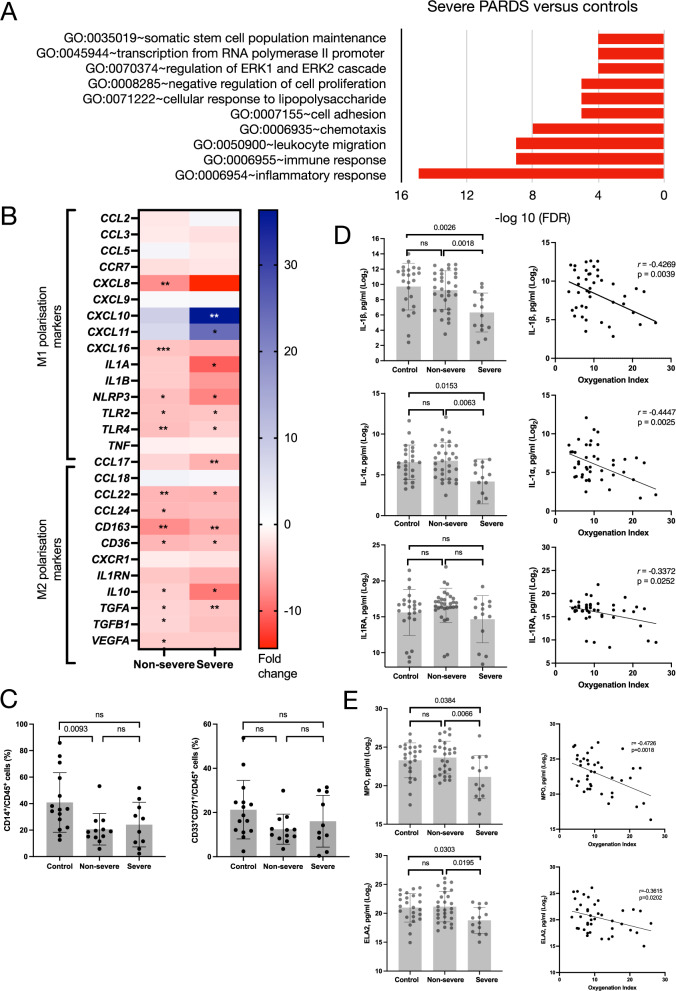
Derangements in the immune response of CD14^+^ myeloid cells from the lung microenvironment of patients with PARDS. Immune cells from deep tracheal lavage samples were sorted using fluorescence-activated cell sorting for CD14^+^ myeloid cells (PARDS at diagnosis, *n* = 22 and controls, *n* = 15). Myeloid cell subsets were then quantified using flow cytometry and their transcriptomic gene expression profiled using *NanoString* (PARDS at diagnosis, *n* = 18 and controls, *n* = 11). IL-1α, IL-1β and IL-1RA concentration within the deep tracheal lavage fluid was quantified using ELISA and dilution was normalized using the urea correction method (PARDS at diagnosis/ progression, *n* = 40 and controls, *n* = 24). **A**Top 10 downregulated gene ontology pathways enriched in CD14^+^ myeloid cells of severe PARDS vs. control. **B** Heatmap of M1 and M2 macrophage polarisation genes in severe and non-severe PARDS vs control. Two-tailed *t*-test was used without correction for multiple testing (*p < 0.05, **p < 0.01, ***p < 0.001). **C** Percentage of myeloid cell subsets of total CD45^+^ immune cells in the lung microenvironment (median, interquartile range). A two-tailed Mann-Whitney U test was used for two-group comparisons of cell subset frequency between severe PARDS vs control, non-severe PARDS vs. control and non-severe vs. severe PARDS. Each data point refers to an independent patient/control sample. **D** IL-1α, IL-1β, IL-1RA, and **E** myeloperoxidase (MPO) and neutrophil elastase (ELA2) concentration in the deep tracheal lavage fluid in each comparison group (mean, standard deviation) and its correlation (Pearson’s, *r*) with the oxygenation index. ELISA was performed in duplicates with the average presented, each data point refers to an independent patient/control sample. Two-tailed Student’s *t*-test was used for two group comparisons of cytokine concentration between severe PARDS vs. control, non-severe PARDS vs. control and non-severe vs. severe PARDS. Only significant *p* values after Bonferroni correction were reported (non-significant, ns).

In contrast to the pulmonary CD3^+^ T cells, the pulmonary CD14^+^ myeloid cell population showed a general down-regulation of immune pathways in severe PARDS compared to controls (Fig. 2A). The transcriptome of the myeloid cells was downregulated for biological processes involving “response to lipopolysaccharide” and “chemotaxis”. Notably, the biological process (GO:0006954) relating to the biological process of inflammatory response, which was upregulated in the CD3^+^ T cells, showed the greatest degree of downregulation in the pulmonary CD14^+^ myeloid cells. Even though the CD14^+^ myeloid cells exhibited an overall dampened inflammatory and immune response, when individual genes were examined in the severe PARDS group, the top 10 upregulated genes were mostly interferon-related (*CXCL10*, *ISG15*, *CCL8*, *CXCL11*, *IFIT1, SIGLEC1, PSMB9, MX1, CCL7, IRF7*) (Table S4).

Gene expression levels of key monocyte markers associated with the classical, intermediate and non-classical monocyte subsets were downregulated along with macrophage M1 and M2 polarisation markers, especially those associated with the M1 markers (Fig. 2B). When examined in a targeted manner, a signature with downregulation of *NLRP3*, *IL1A*, *IL1B*, and *CXCL8* was evident in the *NanoString* dataset obtained from patients with severe PARDS compared to control.

Quantitatively, the proportion of pulmonary CD14^+^ cells was significantly reduced in DTL samples from PARDS vs controls (Fig. S1), in particular non-severe PARDS vs. controls (Fig. 2C). Furthermore, consistent with the gene expression signature, a reduction in IL-1α and IL-1β levels was measured in the DTL samples across the severity groups, with a negative correlation with the oxygenation index (Fig. 2D). Similarly, the levels of IL-1RA, a naturally occurring antagonist by competitive inhibition at IL-1R1, showed a negative correlation with the oxygenation index (Fig. 2D). To understand if these cytokine changes were related to the inflammasome pathway, we evaluated IL-18 as a corroborative marker of inflammasome activation however it had no relationship with PARDS severity (Fig. S2). Finally, a negative correlation was observed between the oxygenation index and MPO and ELA2, which represent downstream IL-1β effector function, neutrophil activation and migration (Fig. 2E). For patients who progressed in PARDS disease severity, disease progression was associated with a further reduction in cytokines IL-1α, IL-1β and IL-1RA (Fig. S2), though this was not significant after Bonferonni correction. In the independent validation cohort, this reduction in IL-1β levels across the PARDS severity groups, negative correlation with the oxygenation index and trend over disease progression was consistently found (Fig. S3).

### Single-cell RNA sequencing reveals T cell and alveolar myeloid subsets that are highly diverse in PARDS

**Fig. 3:**
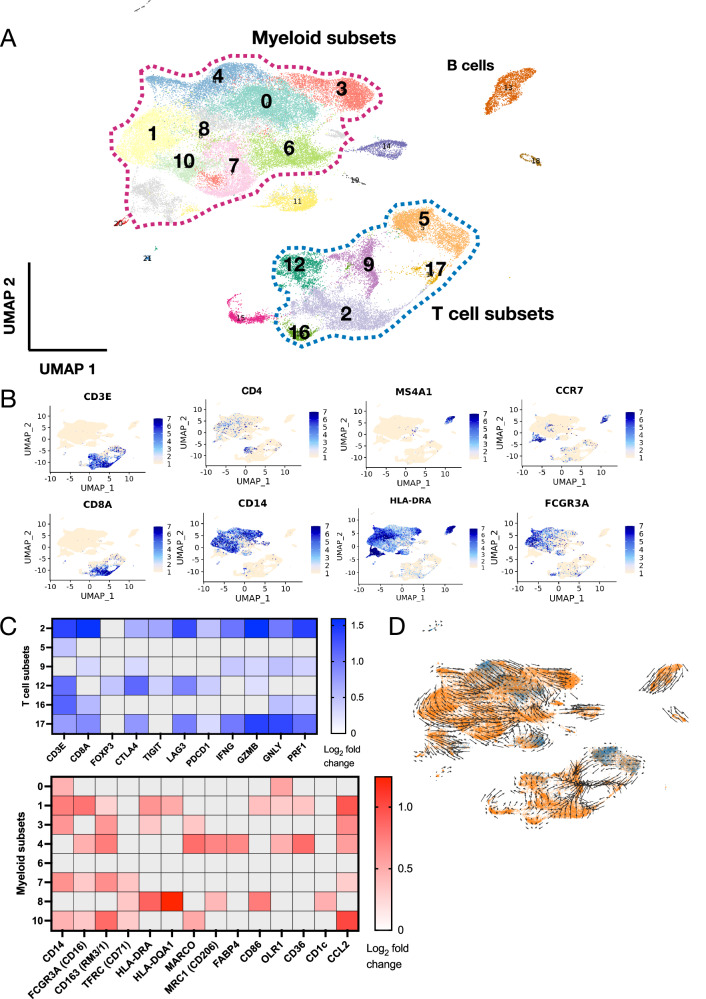
Single-cell RNA sequencing of immune cells from deep tracheal lavage samples from patients with PARDS and controls. Immune cells from deep tracheal lavage samples were sorted using fluorescence-activated cell sorting for CD45^+^ live cells. Single-cell RNA sequencing was then performed on these cells using the 10X Genomics pipeline (PARDS at diagnosis and at extubation, *n* = 10 and controls, *n* = 4). **A** UMAP of 22 unique immune cells clusters from PARDS and control. **B** Lineage gene markers overlayed onto the UMAP. **C** Heatmap of T cell and myeloid cell genes. **D** RNA velocity.

To get further resolution of the heterogeneous pulmonary cellular environment and validate the *NanoString* transcriptomic analysis above, we performed single-cell RNA sequencing. We identified 22 unique cell clusters, 8 of which were of myeloid lineage and 6 of T-cell lineage (Fig. 3A, B). As anticipated, the most abundant cell types were alveolar myeloid cells followed by T cells (Figs. S4–5, Table S5). The top 5 marker genes in each cluster are presented in Fig. S6.

**Fig. 4:**
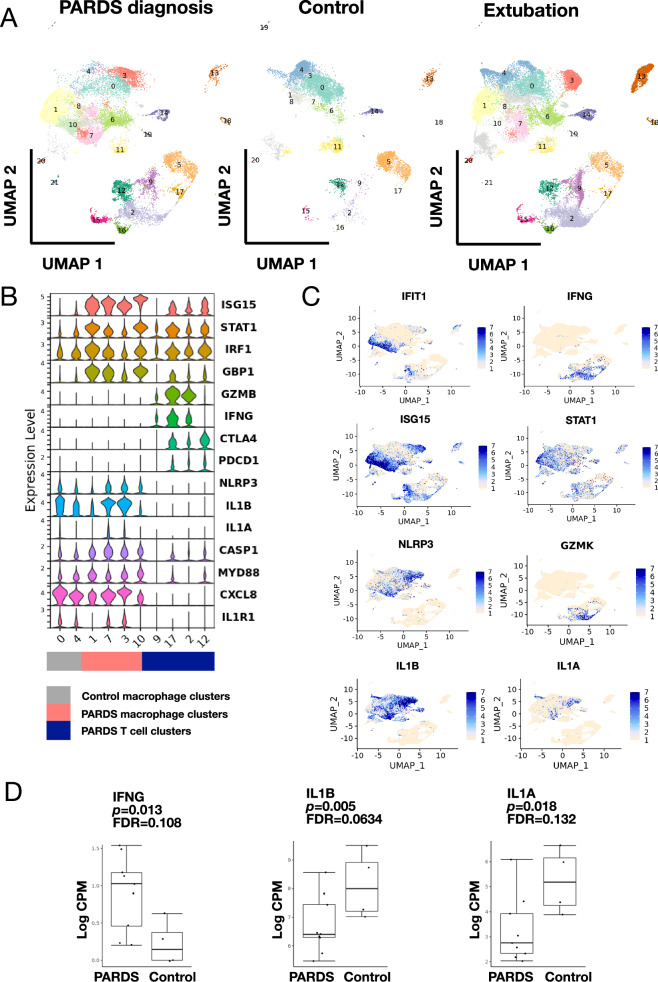
Differential gene expression of single-cell RNA sequencing cells clusters from deep tracheal lavage samples from patients with PARDS and controls. Immune cells from deep tracheal lavage samples were sorted using fluorescence-activated cell sorting for CD45^+^ live cells. Single-cell RNA sequencing was then performed on these cells using the 10X Genomics pipeline (PARDS at diagnosis and at extubation, *n* = 10 and controls, *n* = 4). **A** UMAP of 22 unique immune cells clusters from samples taken at PARDS diagnosis (left), control (middle) and extubation (right). **B** Violin plot of cell clusters and their log-normalized gene expression profile for selected *IL1* and *IFNG* gene pathway. **C** T cell and macrophage functional markers overlayed onto the UMAP. **D** Pseudobulk analysis of *IL1A* and *IL1B* gene expression (Log CPM) from all myeloid cell clusters, and *IFNG* gene expression from T cell clusters. The exact test as implemented in the *edgeR* package was used for differential gene expression analysis between PARDS and control patients. Box plot – box depicts the median, first and third quartiles, whiskers depict minimum and maximum values that are not outliers, each data point refers to an independent patient/control sample.

Overall, we identified 6 T-cell clusters, of which 4 expressed *CD8A*, one cluster expressed genes consistent with Tregs (*FOXP3, CTLA4, IL2RA –* cluster 12) and one considered to be a double-negative T-cell subset for the lack of both CD4 and CD8 markers (cluster 5) (Fig. 3C). Cluster 16 expressed T-cell receptor γδ variable genes associated with a γδT cell phenotype. Naïve T cell markers (*SELL, TCF7*) were expressed by cluster 5 and 16, whereas tissue resident memory T cell markers (*CXCR6, ITGA1*) were expressed by clusters 2, 9 and 17. Effector (*GZMB, GNLY, IFNG, CCL5*) and exhaustion (*CTLA4, LAG3, PDCD1*) T cell genes were found in clusters 2, 9 and 17. Cell subsets were plotted onto individual uniform manifold approximation and projection for dimension reduction plots (UMAP) for PARDS and control to observe differences in abundance between the groups (Fig. 4A). The abundance of T-cell clusters 2, 9, 16 and 17 was higher in PARDS vs. control patients (Fig. 4A and Fig. S5). To understand this difference, we analyzed RNA velocities to infer cell developmental trajectories (Fig. 3D). This visualization defined activation trajectories from naïve (cluster 5) and interferon response (cluster 2 and 17) to cluster 9 which expresses genes associated with regulation of T-cell activation and proliferation (*PDE3B, CBLB, SKAP1, RORA, CD96, PTPN22*)^22–26^. A summary of the top 20 expressed genes in T-cell clusters can be found in the supplementary material (Table S6).

We identified 8 myeloid subsets (clusters 0, 1, 3, 4, 6, 7, 8, 10) characterized by distinct gene expressions (Fig. 3C). Monocyte cluster 0 was characterized by the presence of monocyte markers (*FCN1, VCAN*) and the absence of macrophage markers^27–29^. This monocyte cluster demonstrated a pro-inflammation phenotype expressing *IL1B, S100A8, S100A9*, and *CXCL8* genes. Whereas alveolar macrophage (AM) clusters 4, 7 and 10 were characterized by the expression of *CD163, TRFC, MARCO, MSR1, MRC1*, and* FABP4* macrophage genes^27–29^. Of these AMs, a mature phenotype (cluster 4) was attributed to the expression of genes involved in lipid metabolism (*FABP4, APOC1, APOE*) as well as scavenger functions (*MARCO, MSR1, MRC1*). The other two AM clusters expressed pro-inflammatory genes (*IL6, IL1B, TNFAIP6* - cluster 7) and IFN induced anti-viral genes (*CXCL10, ISG15, DEFB1, MX1* - cluster 10). Two clusters, 1 and 3, were regarded as monocyte-derived macrophages for expressing a combination of both sets of monocyte and macrophage markers. These in turn were also characterized by an inflammatory (*IL1B, S100A8, S100A9*, and *CXCL8* - cluster 3) and IFN response (*CXCL9, CXCL10, IFI27, IFITM3* - cluster 1) phenotype. Finally, we identified a dendritic cell (DC) cluster (cluster 8) characterized by the expression of *CD1C, HLADRA,* and* CLEC10A*. A summary of the top 20 expressed genes in myeloid clusters (Table S7) and a summary comparison of annotation genes with published single-cell datasets (Table S8) can be found in the supplementary material.

The prevalence of myeloid clusters 1 (IFN response monocyte-derived macrophage), 3 (inflammatory monocyte-derived macrophage), 7 (inflammatory AM) and 10 (IFN response AM) were higher in PARDS vs. control (Fig. 4A and Fig. S5). Along with cluster 4 (mature AM), these disease clusters had high RNA velocities indicating that they were actively transitioning cells. The direction of RNA velocity showed that cells originating from these clusters converge into the indeterminate cluster 6 (Fig. 3D). This population exhibits a lack of any distinguishing lineage or functional markers and may represent a terminal subset of cells.

An increase in IFN-γ and its pathway activation genes (*STAT1*, *ISG15*, *IFIT1*) were found in T-cell clusters (cluster 2, 9, 17) enriched in PARDS (Fig. 4B, C). In a pseudo-bulk RNA analysis of concatenated T cell clusters, a significant increase in IFN-γ expression was detected in PARDS compared to controls (Fig. 4D). Correspondingly, IFN response genes (*IFIT1*, *IFI27*, *ISG15*, *CXCL10*, *CXCL11*) were expressed in disease myeloid clusters (1, 3, 7, 10) and were absent in the control myeloid clusters (cluster 0 and 4). Disease myeloid cell clusters with a higher expression of IFN response genes were observed to have a lower expression of IL-1 genes (*NLRP3, IL1A, IL1B, IRAK1, CXCL8*) (clusters 1 and 10) (Fig. 4B). Whereas control and disease myeloid clusters expressing low or absent IFN response genes had a higher expression of IL-1 genes. Pseudo-bulk analysis of disease vs control myeloid clusters demonstrated decreased *IL1A* and *IL1B* gene expression (Fig. 4D).

### The circulating immunome is distinct between pediatric acute respiratory distress and healthy exhibiting the dampened IL1 signature in circulating myeloid cells but not the IFN-γ response signature in the circulating T cells

**Fig. 5:**
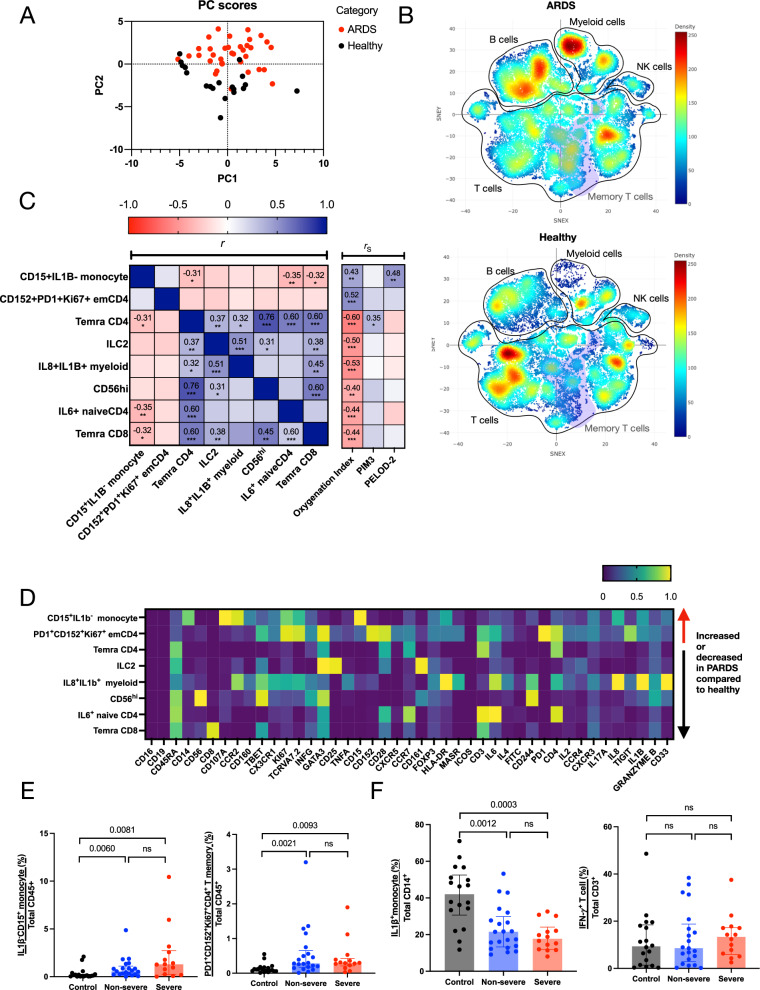
The systemic immunome of patients with PARDS. PBMCs of patients with PARDS at diagnosis, *n* = 35 and controls, *n* = 19 were studied using mass cytometry. **A** PCA plot of all mass cytometry included markers – PARDS (red dots) and control (black dots). **B** Density plots of immune cells from patients with PARDS and healthy controls. **C** Correlation matrix of significantly increased or decreased subsets (Pearson) and their correlation with clinical markers of severity (Spearman). **D** Heatmap of increased and decreased cell subsets in PARDS vs. controls and their protein expression profile. **E** Bivariate gating to verify frequencies of CD15^+^IL-1β^-^ monocyte and PD1^+^CD152^+^Ki67^+^ memory CD4 subsets (median, interquartile range) identified on unsupervised FlowSOM clustering and **F**. frequency of IFN-γ^+^ T cells and IL-1β^+^ monocytes across PARDS severity groups and control (median, interquartile range). Two-tailed Mann-Whitney U test was used for two group comparisons of cell subset frequency between severe PARDS vs control, non-severe PARDS vs control and non-severe vs. severe PARDS (non-significant, ns). Each data point refers to an independent patient/control sample.

The systemic immunome in PARDS vs healthy was analyzed by mass cytometry. Using 45 unique FlowSOM cell clusters (Table S9), PARDS and healthy controls segregated readily on PCA plot (Fig. 5A). Altered cell frequencies in PARDS and healthy samples could be seen in most major cell lineages on tSNE cell density plot (T cell, B cell, monocytes/myeloid cells and NK cells) (Fig. 5B & Fig. S7). When comparing cell frequencies in samples of PARDS vs healthy, there were 8 cell subsets observed to be significantly different after multiple correction – two were increased (CD15^+^IL-1β^-^ monocyte, PD1^+^CD152^+^Ki67^+^ memory CD4) and six were decreased (temra CD4, ILC2, IL8^+^IL-1β^+^ myeloid cell, CD56^hi^ NK cells, IL6^+^ naïve CD4, and temra CD8) (Fig. 5D). The increase in CD15^+^IL-1β^-^ monocytes and decrease in IL8^+^IL-1β^+^ myeloid cells, respectively, may account for the overall reduction in IL-1β in the circulation.

When further analyzed, the two cell subsets which were raised in PARDS compared to control also demonstrated a stepwise increase across the severity categories from non-severe to severe PARDS (Fig. 5E). In the correlation analysis, the two increased subsets were positively correlated with the oxygenation index and the six decreased subsets were negatively correlated with the oxygenation index (Fig. 5C). This trend was not seen with the PIM3 and PELOD2 scores indicating specificity towards PARDS severity. The decreased subsets were positively correlated with one another, indicating possible related pathways leading to their derangement. On supervised bivariate gating, there was a lower frequency of IL-1β-producing monocytes in severe PARDS compared to controls. (Fig. 5F). However, there was no detectable difference in IFN-γ expression across severity groups in the circulation.

### IFN-γ priming suppresses TLR7-induced IL-1β production in a human monocyte cell culture model

**Fig. 6:**
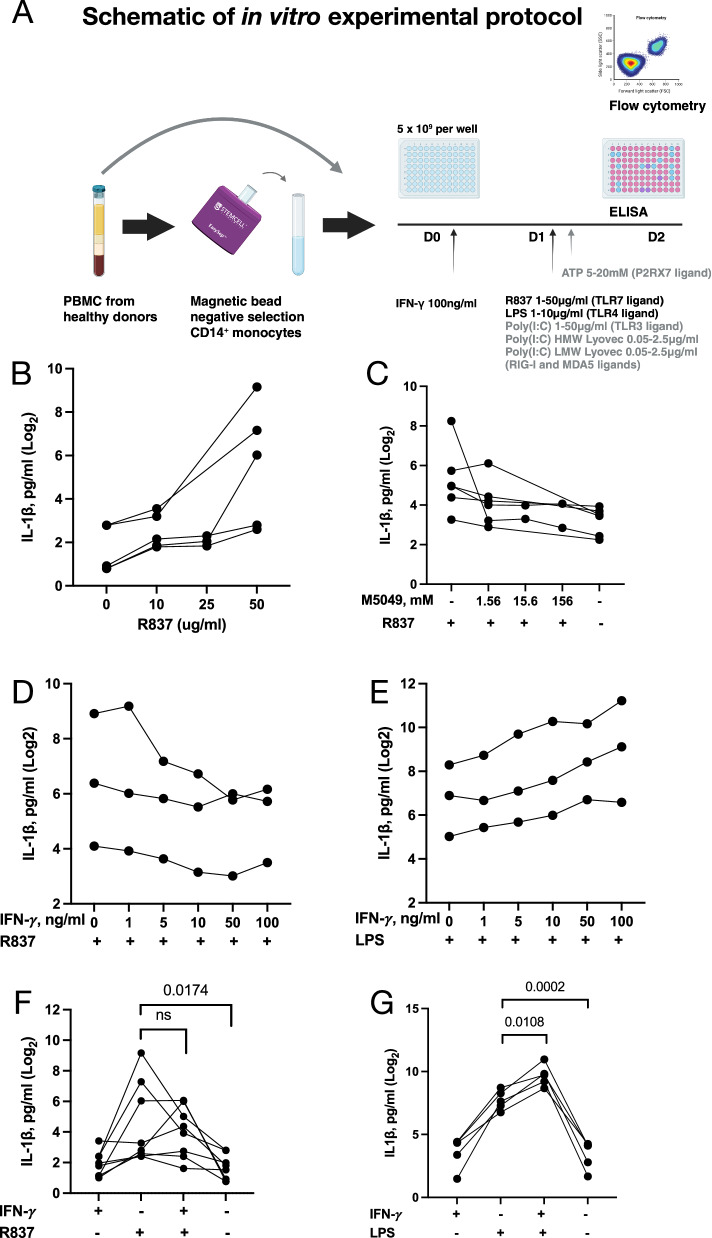
In vitro human PBMC model suggesting the mechanism of IL1 suppression in severe PARDS. **A** Schematic of the in vitro experimental protocol. Results utilizing reagents in *black* for the in vitro cell culture were reported. Reagents in *grey* [Poly(I:C), Poly(I:C) HMW, Poly(I:C) LMW and ATP] were tested but did not produce measureable IL-1β response – these were not presented. IL-1β concentration was measured with ELISA, which was performed in duplicates, with the average presented (Created in BioRender. Wong, J. (2026). https://BioRender.com/1zedwy0). **B** Dose-response plot of IL-1β production across increasing concentrations of R837. **C** IL-1β suppression by increasing doses of M5049 (specific TLR7 antagonist) 3 hours prior to R837 activation. **D** Suppression of R837 (at 50μg/ml) dependent IL-1β production after 24 h priming with increasing concentration of IFN-γ. **E** Enhancement of LPS (at 10μg/ml) dependent IL-1β production after 24 h priming with increasing concentration of IFN-γ. **F** Summary plot of IL-1β production upon activation with 50 μg/ml R837 (*n* = 8 independent samples) and **G** 10μg/ml LPS with and without 100 ng/ml IFN-γ priming (*n* = 5 independent samples). A two-tailed Student’s t-test was used for two-group comparisons. Only significant *p* values after Bonferroni correction were reported (non-significant, ns). Experimental conditions for each donor sample were performed in duplicates, with the average presented; each data point refers to an independent donor sample.

Our multidimensional analyses in the DTL samples revealed concomitant reduction in IL-1β (mostly in the CD14^+^ cell subset) with increased IFN-γ, with both trends correlating with severe PARDS, defined by lower oxygenation levels. To further investigate the relationship and inter-dependence of IL-1β and IFN-γ, peripheral blood mononuclear cells (PBMC) and monocytes from healthy donors were stimulated with R837, a specific TLR7 agonist (Fig. 6A). TLR7 recognises viral ssRNA in keeping with the common triggering agent in PARDS. In PBMC models, a dose-dependent increase in IL-1β concentration was measured in the culture supernatants when stimulated with increasing concentrations of R837 (Fig. 6B). Similar trends were observed with two other TLR7 ligands, CL264 and CL307 (Fig. S8), although the effect was less pronounced than the effect seen with R837. The stimulating effect of R837 on IL-1β production was abrogated when M5049 (a specific TLR7 antagonist) was added to the monocyte culture (Fig. 6C), thus supporting that R837-induced IL-1β production was TLR7-mediated. Next, we demonstrated that the R837-induced IL-1β production was suppressed by priming with IFN-γ and that this effect was also dose dependent on the IFN-γ priming concentration (Fig. 6D), though the IFN-γ suppression was inconsistent across donor samples (Fig. 6F). This suppression was evident in PBMCs from healthy children, but not PARDS patients (Fig. S9). In contrast, IFN-γ priming enhanced IL-1β production when cells were stimulated with LPS (a specific TLR4 agonist) (Fig. 6E, G).

**Fig. 7:**
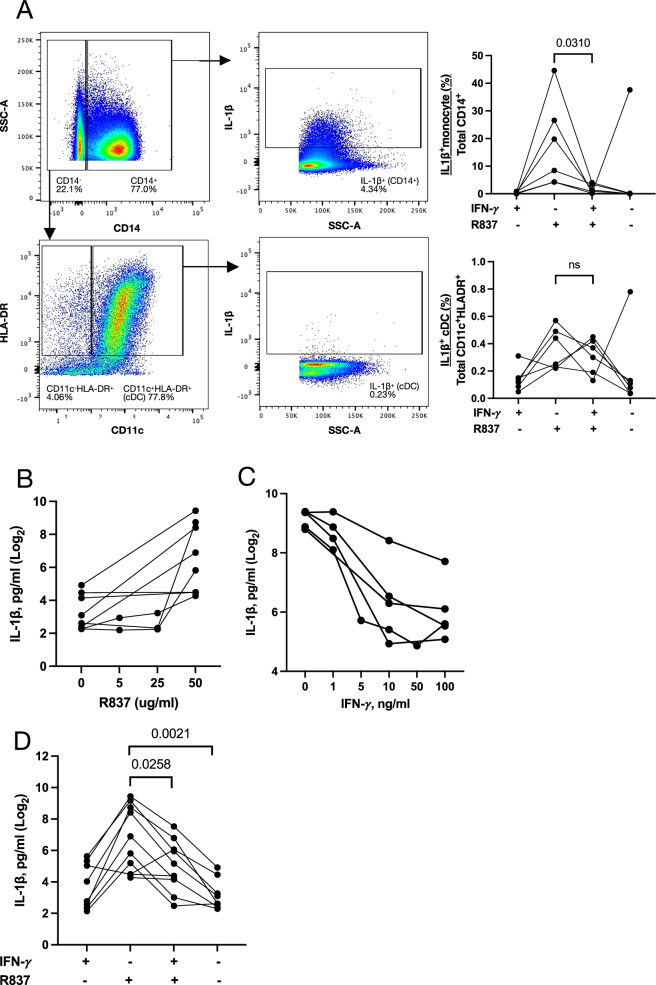
In vitro human monocyte model recapitulating the mechanism of IL1 suppression in severe PARDS. **A** Flow cytometry gating strategy of PBMCs and percentage of IL-1β^+^CD14^+^ monocytes and IL-1β^+^conventional dendritic cells of the parent population upon R837 activation (*n* = 6 independent samples). A two-tailed Mann-Whitney U test was used for the two-group comparison of cell subset frequencies between the indicated experimental conditions. **B** Dose-response plot of IL-1β production in monocytes across increasing R837 concentration. **C** Suppression of R837 (at 50μg/ml) dependent IL-1β production in monocytes by increasing doses of IFN-γ priming. **D** Summary plot of IL-1β production in monocytes upon activation with 50μg/ml R837 with and without 100 ng/ml IFN-γ priming (*n* = 10 independent samples). IL-1β concentration was measured with ELISA, which was performed in duplicates, with the average presented; each data point refers to an independent donor sample. Two-tailed Student’s t-test was used for two-group comparisons of IL-1β concentration between the indicated experimental conditions. Only significant *p* values after Bonferroni correction were reported. Experimental conditions for each donor sample were performed in duplicates (non-significant, ns).

Utilizing a panel to distinguish monocytes and DCs from other cell lineages, flow cytometry identified the cell population within PBMCs reacting to IFN-γ priming and R837 stimulation to be CD14^+^ monocytes (Fig. 7A) - TLR7 stimulation resulted in IL-1β production from 80x more monocytes than DCs. Flow cytometry demonstrated that the reduction in IL-1β production occurred in live monocytes and was not due to reduced cell viability/death (Fig. S10A). Hence, cell cultures were repeated using purified primary human monocytes. Purified monocytes responded to increasing concentrations of R837 with a dose-dependent increase in IL-1β production and exhibited suppression to IFN-γ priming with a dose-dependent reduction in IL-1β production (Fig. 7C). When purified monocytes were primed overnight with IFN-γ, R837-induced IL-1β production was significantly suppressed (Fig. 7D). Collectively, these data indicated that IFN-γ specifically suppressed TLR7-induced IL-1β production in monocytes.

In vitro monocytes and their culture supernatant were examined for IL-1β and relevant proteins in the classical inflammasome pathway by western blot (caspase 1 and gasdermin-D) (Fig. S10B). IL-1β was again shown to be produced upon TLR7 activation by R837 and this was suppressed by IFN-γ priming, however, there was no observable change in band intensity for full length caspase 1, it’s cleaved product p20, full length gasdermin-D or its active N-terminal which may indicate that the activation of the classical inflammasome pathway was not involved in the production of IL-1β by R837 (Fig. S10C).

### Altered gene expression pathways in monocytes undergoing TLR7 activation with and without IFN-γ priming closely resembles gene expression in PARDS patients

**Fig. 8:**
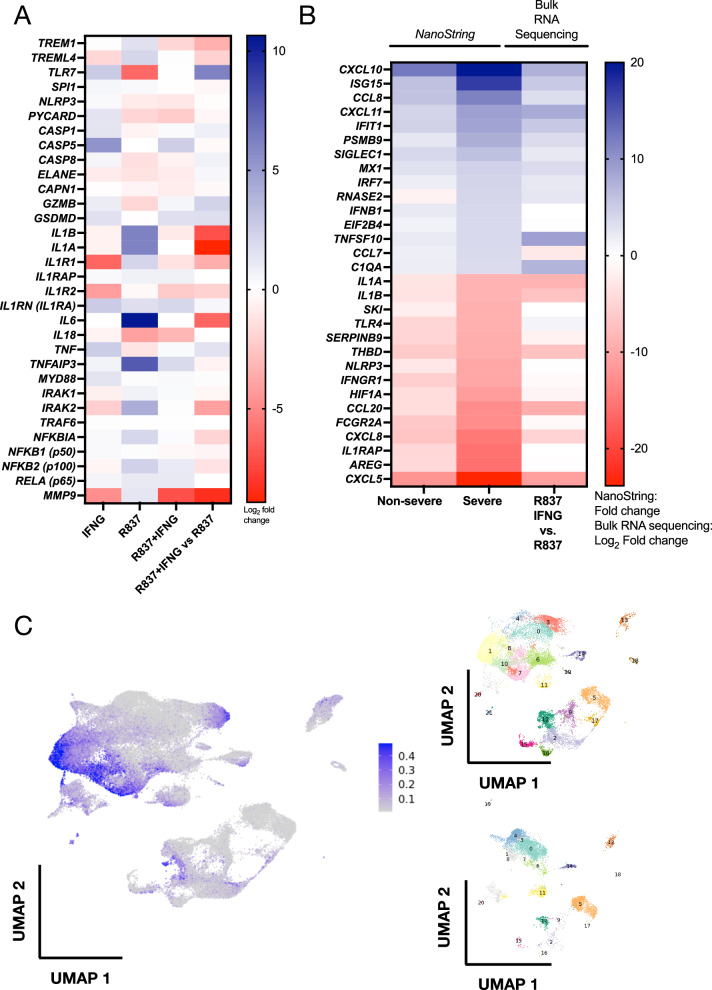
Bulk RNA sequencing of in vitro monocyte conditions mapped alongside NanoString and single-cell RNA sequencing clinical samples. **A** Bulk RNA sequencing of in vitro monocyte conditions; (left to right) IFN-γ priming vs. negative control (PBS alone), activation with 50μg/ml R837 vs. negative control, activation with 50μg/ml R837 with IFN-γ priming vs. negative control and activation with 50μg/ml R837 with IFN-γ priming vs. R837 alone (*n* = 5 independent samples). **B** Juxtaposition of bulk RNA sequencing of monocytes activated with 50μg/ml R837 with IFN-γ priming vs. R837 alone with *NanoString* non-severe and severe PARDS samples. **C** Mapping of gene module from bulk RNA sequencing of monocytes activated with 50μg/ml R837 with IFN-γ priming vs. R837 alone onto the single-cell RNA sequencing UMAP of clinical samples.

Bulk RNA sequencing was performed on IFN-γ-primed or unprimed monocytes treated with R837. Gene set enrichment analysis identifying the top up/down regulated gene ontology pathways are presented in an over representation analysis (Fig. S11). In the absence of IFN-γ priming, when R837-stimulated monocytes were compared with unstimulated monocytes, upregulated pathways included “inflammatory response” and “cellular response to lipopolysaccharide” (Fig. S11A). Specifically, activation of TLR7 with R837 resulted in upregulation of genes for IL-1β, IL-1α, IL-1R1, IL6 and downstream NFκB transcription factor (Fig. 8A). It was noted that TLR7 activation did not modify transcription of genes in the NLRP3 canonical and non-canonical pathway nor genes encoding proteolytic enzymes involved in the processing of mature IL-1α and IL-1β.

However, when comparing (IFN-γ and R837) vs. (R837 alone), “chemokine signalling”, “neutrophil chemotaxis”, “monocyte chemotaxis” and “cellular response to IL-1β” pathways were downregulated (Fig. S11B). Similarly, when comparing (IFN-γ and R837 vs IFN-γ alone) “regulation of IL-1β production” and “regulation of NFκB signal transduction” pathways were downregulated in the former (Fig. S11C). Specifically, the expression of genes for *IL1B, IL1A, IL1R1, IL6* and downstream NFκB transcription factor were dampened in IFN-γ primed monocytes (Fig. 8A) but not *TNFA* and *IL18*. Hence, the bulk RNA sequencing data indicated that IFN-γ-mediated suppression of TLR7-induced IL-1β production involved the suppression of TLR7-mediated signalling, including downstream NFκB activation.

To further explore the relevance and adequacy of the in vitro monocyte culture model to recapitulate some of the immune perturbations observed in PARDS samples, we juxtaposed the top 15 up and downregulated genes in severe PARDS (derived from *NanoString* dataset) against the same genes expressed from the bulk RNA sequencing of cultured monocytes in the (IFN-γ and R837) vs. (R837 alone) condition, in a heatmap (Fig. 8B). This demonstrated that the overall direction of differential gene expression was similar in the in vitro model and clinical samples. Significantly up and downregulated genes from the bulk RNA sequencing (IFN-γ and R837) vs. (R837 alone) was used to develop a gene module score, which was then overlayed on the UMAP from single-cell RNA sequencing of PARDS samples (Fig. 8C) – this gene module mapped directly onto the UMAP coordinates of disease clusters 1, 3, 7 and 10. Both these visualisations supports the suggestion that the gene expression profile from the in vitro cell culture closely resembles the gene expression profile in our PARDS cohort.

To contextualize whether the identified signatures were unique to PARDS or conserved across age groups, we compared our data with two available published single-cell RNA sequencing datasets. The first, a pediatric single-cell RNA sequencing dataset of tracheal aspirates from young children with PARDS, we observe a similar IFN-gene response high–*IL1B* gene low phenomena in *CD14* expressing myeloid cells (Fig. S12). In this comparison dataset, 12 patients with PARDS were included – 11/12 (92%) had a viral pathogen detected, of which 7/12 (58%) had RSV. Using the monocyte in vitro cell culture bulk RNA sequencing gene module, upregulated genes correspond to IFN-stimulated clusters and downregulated genes correspond to *IL1B* expressing clusters. The second, an adult critical coronavirus disease 2019 (COVID-19) bronchoalveolar lavage dataset, an enriched population of CD4^+^, CD8^+^ T cells and monocytes exhibiting a strong *IFNG* response was observed. Here, though there were cell clusters co-expressing IFN-response genes and *IL1B* genes (TRAM2 and MoAM1), there were also clusters which expressed IFN response genes (*CXCL10*) without *IL1B* genes (MoAM2). Applying the monocyte in vitro cell culture bulk RNA sequencing gene module showed that cells expressing *ISG15* and *STAT1* expressed little *IL1B* (Fig. S13).

## Discussion

Through the adoption of a high-dimensionality analytical approach, this study identifies three potential pathogenic immune mechanisms in PARDS. CD8^+^ T cells exhibited a cytolytic, exhaustion/ inhibition, and apoptosis genotype corresponding to a muted increase in CD8^+^ T cells frequency in the severe PARDS group. Severe PARDS was further characterized by elevated expression of interferon-related genes in both CD3^+^ T cells and CD14^+^ myeloid cells. Distinct macrophage subsets expressing interferon-stimulated genes demonstrated concurrent downregulation of IL1 pathway genes, which was associated with diminished leukocyte migration, chemotaxis, phagocytosis, and M1 polarization pathways. Decreased IL-1β and increased IFN-γ levels in DTL fluid were positively correlated with severe viral PARDS. These pathological immune features were reproduced in a human primary monocyte cell culture model, where the suppressive effect of IFN-γ on TLR7-induced IL-1β was evident.

CD8^+^ T cell cytotoxicity was closely linked to the severity of PARDS and may contribute directly to lung injury. This relationship highlights the potential impact of these immune cells on disease progression and tissue damage in the lungs. Supporting evidence from single-cell RNA sequencing analyses of bronchoalveolar lavage (BAL) fluid in adults with COVID-19-induced ARDS revealed the presence of cytotoxic T-cells in cases of moderate and severe ARDS^30–32^. Notably, cytotoxic CD8^+^ T-cells expressing granzyme B (GZMB^+^) were found to infiltrate the lungs of patients who succumbed to ARDS during the acute phase of diffuse alveolar damage, which is a hallmark histological feature of ARDS^33^. In that same study, only a limited number of CD8^+^ T cells expressed immune checkpoint markers such as CTLA4/CD25 and PD1. This finding suggests that, in adult ARDS, immune checkpoint regulation of T cells may be less prominent. In contrast, transcriptomic data from our study indicated an upregulation of multiple immune checkpoint and apoptosis-related genes within CD8^+^ T-cell clusters in severe PARDS. The enhanced expression of these genes may lead to T-cell exhaustion, thereby restricting further proliferation and cytotoxic activity, thereby modulating the extent of lung injury. Further studies are required to confirm if infiltrating CD8^+^ T-cells in PARDS indeed exhibit higher levels of immune checkpoint gene expression compared to those in adult ARDS.

The second prominent feature from the PARDS transcriptome was the strong IFN response which aligns with the high prevalence of viral-induced PARDS in the study population. Within both CD3^+^ T-cells and myeloid cells, the most upregulated genes were interferon-stimulated genes, highlighting the central role of IFN signaling in the immune landscape of PARDS. Though no direct data is available on the role of IFN-γ in PARDS, animal studies and adult data provide consistent findings of high IFN-γ in the setting of ARDS. In a murine model of ARDS, IFN-γ has been shown to induce pulmonary inflammation, drive hypercytokinemia (TNFα, IL-1β, IL6) and contribute to mortality^34^. Similarly, in adult cases of moderate and severe COVID-19-related ARDS, single-cell RNA sequencing data revealed activation of IFN signaling pathways in circulating CD8^+^ T cells, with a clear correlation between this activation and disease severity^32^. These findings in severe COVID-19 ARDS are consistent with observations in PARDS, suggesting close parallels in the role of CD8^+^ T cells and IFN signaling between pediatric and adult forms of the disease. However, a notable difference arises in the transcriptomic response of monocytes and macrophages. Unlike the robust IFN signaling seen in adult COVID-19 ARDS^32^, the transcriptomic data from PARDS indicate a deficient response to interferon signaling in monocyte and macrophage populations, which stands in contrast to previous adult studies.

An unexpected decrease in IL-1 pathway genes and proteins was observed in macrophages from patients with severe, viral-induced PARDS, despite a high expression of interferon-stimulated genes within these cells. Specifically, we noted reduced IL-1β-related gene expression in IFN-stimulated myeloid clusters (clusters 1 and 10). Nevertheless, other clusters associated with PARDS (clusters 3 and 7) showed upregulated IL1B expression, suggesting that, at the time of sampling, diverse cell populations were concurrently present in the lung microenvironment. These findings are in agreement with single-cell RNA sequencing data from both pediatric PARDS and adult critical COVID-19 cases, which demonstrate heterogeneous monocyte and macrophage populations exhibiting varying pro-inflammatory profiles^35,36^. This cellular heterogeneity indicates that further research is necessary to fully understand the contributions of each cell population to the progression and severity of the disease. The coexistence of these cell clusters influences the lung secretome, and our study demonstrated an overall reduction of IL-1β in severe disease states. IL-1 is a potent pro-inflammatory cytokine essential for pathogen clearance and host survival, and it typically increases during lung infections^37^. A host of bacterial (including staphylococcus aureus, streptococcus pneumoniae and Gram negative bacteria), viral (including influenza virus and adenovirus) and fungal (including candida albicans and aspergillus fumigatus) pathogens known to trigger ARDS, have been documented to induce IL-1 production in the human host^38^. Lung injury in the context of acute respiratory infection is believed to result in part from these host-driven inflammatory processes involving IL-1^37^. IL-1 production is closely associated with inflammasome activation, although inflammasome-independent IL-1 production has also been documented^39^. Our study found that IL-18, an inflammasome-related cytokine and IFN-γ inducer, did not show a correlation with disease severity in PARDS patients, and inflammasome genes were not differentially expressed in the in vitro model. These results suggest that the observed changes in IL-1 are likely independent of inflammasome activity. In adult ARDS, IL-1β hypersecretion has been documented in cases with traditional ARDS etiologies^40–42^ as well as in viral-induced ARDS, such as COVID-19^30,32^. In contrast, the majority of PARDS patients in our cohort had viral or presumed viral infections and exhibited a suppressed IL-1 signature. It remains to be determined whether patients with bacterial-only etiologies experience a similar immune response. Based on these observations, we hypothesized that the elevated IFN-γ levels induced by viral infection are responsible for the suppression of IL-1 in severe PARDS.

Although the in vitro monocyte model utilized healthy adult PBMCs or monocytes, we believe that it can recapitulate the immune pathophysiology of PARDS. Children have been shown to express a high basal interferon tone as compared to adults in both the healthy state and during acute viral infection (COVID-19)^43^. This was postulated to be due to frequent and recurrent asymptomatic viral infections in childhood. We postulate that the higher interferon tone behaves as the priming effect seen in our in vitro monocyte model from adult donors. Indeed, PBMCs from healthy children consistently suppressed IL-1β production upon IFN-γ priming. However, children with PARDS who had been recently exposed to a respiratory viral infection (and presumably developed an interferon response in vivo) showed no further suppression when primed in vitro with IFN-γ. Patients who develop severe PARDS possibly experience an intense IFN-γ response, are hyperresponsive to the IL-1 suppressive effects of IFN-γ or already have a high basal expression of interferon, resulting in low IL-1β expression. A study to directly compare pediatric and adult IFN-γ and IL-1β responses during viral ARDS would be necessary in the future to confirm this hypothesis.

The suppressive effect of IFN-γ on IL-1β production has been reported before albeit under different conditions. In murine models, TLR4 stimulation of bone marrow derived macrophages describe that IFN-γ priming inhibits the p65 region of NFκB from binding to the IL-1β promoter region which directly reduced *IL1B* mRNA expression^44,45^. It was suggested that an epigenomic mechanism was responsible for this effect – H3K9 and H3K14 acetylation on the *IL1B* promoter region is induced by LPS, but when acetylation was inhibited by IFN-γ priming, chromatin accessibility was reduced and *IL1B* transcription reduced^45^. Separately, type I interferon priming was shown to inhibit inflammasome activation through LPS-induced IL-10 via a STAT3-dependent mechanism^46^. However, we showed that IFN-γ priming instead enhanced TLR4-induced IL-1β production in human monocytes. Indeed, the effect of IFN-γ priming on TLR4 activation may be species specific^47–49^ and the described epigenetic mechanism may not be preserved in humans. Other potential mechanisms include the IL1 antagonizing effect of IL10^50^ and IL-1RA^51^, however, both were not raised in PARDS patients or in the in vitro cell model and therefore, cannot account for our observations. Further studies are thus required to determine the mechanism involved in the suppressive effect of IFN-γ on IL-1β production in PARDS.

Lastly, there were notable similarities and differences between the alveolar macrophage phenotype described in this study and previous reports. Existing flow cytometry studies inform that the alveolar space is infiltrated with macrophages whose cell frequency is increased in adult ARDS compared to controls^5^. These CD14^+^ macrophages are thought to resemble peripheral blood monocytes that have migrated from the circulation into the alveolar compartment as they demonstrated increased expression of CD14, CD11b, CD71 and HLA-DR, and decreased expression of 25F9 (marker of mature tissue macrophages). Pro-inflammatory 27E10 (*S100A8* and *S100A9*) expression was high and macrophage marker RM3/1 (*CD163*) expression was low^5^. This is supported by other single-cell RNA sequencing reports^27–29^ and consistent with the results from our study which identify a similar set of macrophage markers – in the single-cell RNA sequencing data macrophage clusters expressed various combinations of *CD14, CD163, CD71, CD86, MARCO, MRC1/CD206, OLR1, FCGR3A, HLA-DQA1, S100A8, S100A9* and *HBEGF* genes associated with human alveolar macrophage identity^52^. A high expression of the chemotaxis gene, *CCL2*, in our data was found in disease samples and the presence of *CD14* expression is supportive of the notion that migrating monocytes contribute to the monocyte/macrophage population in the lung during PARDS. However, several differences exist. The most striking difference is that though macrophages remain the most numerous cell type, the cell frequency was observed to be decreased in PARDS rather than increased, as reported in adult ARDS. Marker CD206/*MRC1* was not uniformly expressed in all macrophage clusters, and CD169 (*Siglec-1)* expression was not differentially expressed in PARDS. In contrast, CD163 (thought to be a marker of tissue resident macrophages) expression was upregulated by almost all macrophage cell clusters (cluster 1, 3, 4, 7, 10), including the disease clusters.

Our work demonstrates similarities and differences between adult ARDS and PARDS with regards to cytolytic CD8^+^ T cell infiltration, interferon responses by both T cell and macrophages and the suppressed IL1 pathway in macrophages. However, there are some limitations to acknowledge in our work. Due to the low alveolar macrophage cell number obtained from DTL, we were unable to perform functional studies on these primary cells. Instead, we used healthy donor monocytes isolated from PBMCs. Secondly, due to the cryopreservation process, we were unable to study the role of neutrophils in PARDS. Instead, we studied surrogate markers of neutrophil activation and migration, including neutrophil elastase and myeloperoxidase. And lastly, though BAL is considered the gold standard method of sampling of alveolar lining fluid, bronchoscopies are rarely performed in children. As such, we adopted the DTL method which is similar to a blind BAL. Though the cellular composition of BAL and DTL was shown to be similar, there may be minor differences between the two sampling methods^53^. A key distinction lies in the variability of the return volume, which introduces differences in the dilution factor across samples. To account for this variability, we expressed cell subset data as a percentage of total CD45^+^ cells within each lavage specimen rather than absolute cell counts. We confirm that the cellular composition of DTL yielded macrophages with alveolar macrophage phenotype indicating their likely origin from the alveolar space. As the majority of cases included in this study had direct PARDS triggers, future work should examine differences, if any, between indirect and direct ARDS over a wide age spectrum. Additionally, pathophysiolocial evidence from in vivo studies will provide more robust evidence of biological relationships described in this study.

In conclusion, we adopted a high dimensionality approach to understand the immune dysregulation in PARDS. Three main pathogenic immune mechanisms were found to be associated with the severe PARDS phenotype; infiltrating CD8^+^ T cells exhibit exhaustion/ inhibition and apoptosis markers which may play a role in regulating cytotoxic inflammation in PARDS, interferon responses were evidenced in both T cell and macrophages, and IFN-γ induced IL-1β suppression in macrophage subsets. There are similarities and differences between the pathogenic mechanisms involved in PARDS and previously published adult ARDS data, therapies may not be extrapolated without further pediatric-specific translational studies.

## Methods

### Patient recruitment and ethics approval

Biological samples from children diagnosed with PARDS were obtained from patients who fulfilled the PALICC 2015 definition of PARDS^54^. This study screened and enrolled consecutive patients with PARDS over January 2018 to August 2023. Patients received protocolized mechanical ventilation adherent to lung protective strategies, and an extubation readiness test ensuring timely extubation. The study protocol was approved by the Singhealth centralized institutional review board (CIRB reference number 2017-3076). In this study, viral PARDS was defined as PARDS caused by pneumonia, whereby a viral or no pathogen (presumed viral) was identified; this included mixed infections where a virus was identified. An independent cohort under the same study protocol was recruited from the same site over September 2023 to December 2024 as a validation cohort. Sex/gender was not anticipated to influence the findings and was not considered in the study design. DTL samples were obtained concurrently with the blood samples within 72 hours of developing PARDS, indicating the acute phase of the disease.

Biological samples from control patients were obtained from two sources. DTL samples were obtained from patients admitted post-elective surgery with no known lung disease (CIRB reference number 2015–2221). For blood samples, patients were enrolled pre-anesthesia for elective day surgery with no indication of inflammation (CIRB reference number 2019–2961). In vitro cell culture experiments were conducted using cryopreserved PBMCs from healthy adult blood donors (CIRB reference number 2018–2676). Informed consent was obtained from volunteers or a parent and/or legal guardian of all patients before entrance into the study.

### Flow cytometry

Single-cell suspensions of DTL or PBMC were incubated with fluorochrome-tagged antibodies (Table S1). Samples were analyzed using a multicolor BD FACSAria II SORP (Becton Dickinson) instrument, and the data were analyzed using FlowJo software. The expanded description and gating strategy used are presented in respective results sections to profile the major immune lineages in the lung of patients with PARDS (PARDS *n* = 23, controls *n* = 15), sort live CD3^+^ and CD14^+^ cells for *NanoString* transcriptomic analysis, sort live CD45^+^ immune cells for single-cell RNA sequencing, and profile myeloid lineage markers of PBMCs in the in vitro cell culture.

### NanoString

CD3^+^ and CD14^+^ cells purified with FACS were treated with RNA extraction buffer (PicoPure^TM^ RNA isolation kit, Thermo Fisher Scientific) for transcriptomic analysis on the *NanoString* platform (PARDS *n* = 18, controls *n* = 11). RNA amplification, hybridization and post-hybridization processing were performed according to manufacturer’s recommendations. The *NanoString* Human Immunology 2 and Human Myeloid Innate Immunity CodeSet, consisting of 594 and 770 human immune-related genes, were used to interrogate the sorted CD3+ and CD14+ cells, respectively. The raw digital counts of gene expression were exported to *nSolver* version 4.0 software for downstream analysis.

### Single-cell RNA sequencing

CD45+ live cells were FACS sorted targeting 16,000 live CD45+ cells at a final single cell suspension of 1000 cells/uL, which was then loaded on the 10X Genomics chip for each sample (PARDS *n*=10, controls *n*=4). Single cells were subsequently encapsulated into droplets via a gel bead in the emulsion (GEMs) method using the 10X Chromium controller. For gene expression, the Chromium Single Cell 3ʹ Gene Expression protocol (V3 Chemistry, 10X Genomics) was used. Downstream library construction was done using 10×3ʹ Gene Expression Library Construction kit and barcoded with i7 Illumina adapter indexes. Pooled libraries were then sequenced on the Illumina HiSeq 4000 platform using paired-end 151 bp reads to achieve 50,000 reads per cell for gene expression. Library construction and sequencing were performed in two batches.

Raw sequencing reads were aligned to the Human genome (GRCh38) using *CellRanger* version 7 software. *CellRanger’s* count utility was used to count the features. Cell barcodes and feature count matrices were created by aggregating filtered feature counts of each sample using the *CellRanger* aggregate utility. Subsequent data normalization and analysis were performed using the *Seurat* R package and custom R code. Cell count data were quality controlled and filtered based on cellular complexity (number of genes per cell) and mitochondrial reads. Cells with between 300 and 5,000 genes and mitochondrial reads less than 20% were kept for analysis. Data scaling, normalization, variable gene identification and clustering were performed using the *Seurat* pipeline. *SCTransform* from *Seurat* R package was used for batch normalization. Louvian clustering was performed on batch normalized data. To assign cluster identities, we used a list of canonical immune populations, and their classical markers. The exact test as implemented in the *edgeR* package was used for differential gene expression analysis between PARDS and control patients. Trajectory analysis was performed using *Velocyto* command line software, *scvelo* and *scanpy* Python libraries.

RNA expression profiles from monocytes stimulated with R837 and primed with IFN- γ were compared to those treated with R837 alone to mimic viral infection. Genes significantly upregulated in the presence of IFN- γ during the simulated viral infection were selected to construct a gene module signature. Using this list of upregulated genes, a module signature score was calculated for single-cell RNA-sequencing data obtained from PARDS patients and controls. The *ModuleScore* function from the *Seurat* package in R was employed for this calculation. Finally, the computed module scores were visualized by overlaying them onto the UMAP plot of PARDS patients from this study. In addition we analysed, two published single-cell RNA-sequencing datasets^35,36^. Raw FASTQ or BAM files were downloaded and processed as described in above. The ModuleScore for these datasets was also calculated and overlaid on their respective UMAP plots.

### Mass cytometry

Cryopreserved PBMCs were thawed, rested and stimulated with phorbol 12-myristate 13 acetate (PMA) and ionomycin (both from Sigma-Aldrich) for 5 hours (PARDS *n* = 35, controls *n* = 19). Brefeldin A and monensin (eBioscience) were added during the last 3 h of the incubation for blockade of protein transport. PBMCs were then barcoded and subjected to a surface and intracellular staining protocol (Table S2). Data acquisition was then performed using a XT mass cytometer (Standard BioTools) and output files were normalized using EQTM Six Element Calibration Beads^55^.

To identify cell populations, we applied cytometry analysis using self-organising maps (FlowSOM) clustering after random down-sampling to 10,000 cell events per sample^56^. Protein expression patterns of clusters were examined using dendrogram heat maps constructed using the *heatmaply* R package. Non-linear dimensionality reduction using tSNE and UMAP was performed to visualise multi-dimensional expression landscapes in two dimensions. Non-parametric tests were used to compare the cell frequency between PARDS patients and healthy controls with multiple corrections. Further details of the processing and staining steps, and data analysis can be found in the supplementary material.

### Enzyme linked immunosorbent assay (ELISA)

IL-1α (R&D Systems Inc), IL-1β (Invitrogen), IL-1RA (R&D Systems Inc), interferon gamma (IFN-γ) (Invitrogen), myeloperoxidase (MPO) (R&D Systems Inc) and neutrophil elastase (ELA2) (R&D Systems Inc) from DTL samples were assayed using ELISA according to manufacturer’s recommendations (PARDS *n* = 40, controls *n* = 24). Assay concentrations, which were run in duplicates, were averaged and extrapolated from standard curves. Urea nitrogen concentration was measured by colourimetric detection (Invitrogen), and the urea dilution method was used to standardise DTL to plasma concentration. Concentration of cytokines were measured in pg/ml and Log_2_ transformed prior to analysis on GraphPad PRISM (version 10.0.3) software.

### PBMC and monocyte in vitro cell culture

For PBMC cell culture, cryopreserved healthy donor PBMCs (*n* = 12) were thawed, rested and seeded into 96-well plates at cell density of 5×10^5^ per well with complete cell culture media (RPMI 1640 medium supplemented with 10% (v/v) human serum (Corning) and 1× (v/v) penicillin-streptomycin-glutamine (Gibco). For monocyte culture, the CD14+ monocyte negative selection isolation kit (EasySep Human Monocyte Isolation, Stem Cell Technologies) was used according to the manufacturer’s instructions for monocyte isolation prior to seeding into 96-well plates at the same cell density with complete cell culture media.

Reagents for in vitro cell culture included HMW Poly (I:C) (InvivoGen), Imuquimod/R837 (InvivoGen), CL264 (InvivoGen), CL306 (InvivoGen), HMW Poly (I:C) lyovec (InvivoGen), LMW Poly (I:C) (InvivoGen), ATP (adenosine 5’-triphosphate disodium) (InvivoGen), recombinant human IFN-γ (PeproTech,), Enpatoran (M5049) (InvivoGen) at specified concentrations. PBMCs were examined using flow cytometry (panel antibodies - Table S3) to demonstrate the subset population responding to TLR7 activation and producing IL-1β.

### SDS-PAGE and western blot

Monocyte culture supernatant was removed and protein was precipitated^57^. Briefly, supernatant was combined with equal amounts of methanol and 1/3 amount of chloroform and centrifuged at 14800 g for 10 min. The top aqueus solution was then discarded and another 1.6X the initial volume of methanol added. After centrifuging at 14800 × *g* for 15min, the top solution was removed, leaving behind the protein precipitate, which was combined with the cell extract prior to performing SDS-PAGE.

Monocytes from the in vitro cell culture were lysed in SDS lysis buffer (containing 62.5mM Tris pH 6.8, 2% SDS, 1mM EDTA, 20% glycerol) with 2mM dithiothreitol, and 1× Halt™ Protease and Phosphatase Inhibitor Cocktail, and subsequently boiled for 10min. The DC Protein Assay (Bio-Rad, #5000112) was used to estimate the protein concentration. After which, the sample was diluted with 4× Laemmli sample buffer (Bio-Rad, #1610747), resolved by SDS-PAGE, and then transferred onto 0.2 μm PVDF membranes (Bio-Rad, #1620177). Blocking of the membranes with 5% nonfat milk was performed and membranes were incubated overnight at 4 °C with the indicated primary antibodies. Membranes were then incubated at room temperature with HRP-conjugated secondary antibodies for 1 h. Subsequent detection of chemiluminescence signals was performed using ChemiDoc MP Imaging System (Bio-Rad) with SuperSignal West Pico PLUS Chemiluminescent HRP Substrate (Thermo Scientific # 3457*)*. Image analysis was performed using ImageJ (version 1.53 m) ^58^

The following primary antibodies dilutions were used: Invitrogen: IL-1β (#P420B) 1:500, caspase 1 (#*MA516215*) 1:1,000, gasdermin-D (#*PA5115330*) 1:1,000 and β–actin (#*MA1140*) 1:20,000. The secondary antibodies used were Cell Signaling Technology: Anti-mouse IgG, HRP-linked (#7076) and anti-rabbit IgG, HRP-linked (#7074). All primary antibodies were diluted according to protocols in 5% (w/v) bovine serum albumin in Tris-buffered saline with 0.1% (v/v) Tween-20 (TBST) containing 0.1% (w/v) sodium azide. The concentration of secondary HRP-linked antibodies used was 1:2000 diluted in TBST with 5% (w/v) nonfat milk.

### Bulk RNA sequencing

Bulk RNA sequencing of monocytes from the in vitro cell culture conditions (R837, IFN-γ, IFN-γ + R837 and PBS) were analysed (sufficient RNA, *n* = 6). Total RNA was isolated using the PicoPure RNA-Isolation kit (Arcuturus, Ambion) and cDNA was generated with the SMART-Seq v4 Ultra Low Input RNA Kit for Sequencing (Clontech), according to manufacturers’ protocols. Illumina-ready cDNA libraries were generated from amplified cDNA using the Nextera XT DNA Library Prep Kit (Illumina) and multiplexed for 2×150 bp-sequencing. NGS was performed externally at NovogeneAIT on a NovaSeqXplus platform.

Raw reads files were aligned to the Human genome (GRCh38) using the *STAR.v2* gene alignment programme. Gene counts were calculated using the *featureCount* utility of the *Subread* command-line programme. The *EdgeR* package *exactTest* for two-group comparison was used for differential gene expression analysis. Genes were considered significant with FDR<0.05, log counts per million (Log CPM)>1, and Log_2_ fold change >2 in all the comparisons (IFN-γ vs PBS, R837 vs PBS, IFN-γ+R837 vs PBS and IFN-γ+R837 vs R837 alone).

### Reporting summary

Further information on research design is available in the Nature Portfolio Reporting Summary linked to this article.

## Supplementary information

Supplementary Information

Reporting Summary

Transparent Peer Review file

## Source data

Source data

## Data Availability

The raw count data generated in this study have been deposited in the GEO public repository under accession code GSE327171. The processed data used to produce figures are available in the Zenodo repository (10.5281/zenodo.18843576). Associated data generated in this study are provided in the Source Data file. Source data are provided with this paper.

## Code availability

Code used for the analysis and to produce figures is deposited in the Zenodo repository (10.5281/zenodo.18843576).
