# Supplementary material for: A high dimensionality approach reveals immunopathogenic responses driving severe pediatric acute respiratory distress syndrome
Source: Nat Commun. 2026 May 15;17:6466. doi: 10.1038/s41467-026-73181-2 (PMC13376821; doi:10.1038/s41467-026-73181-2)
Supplement: Supplementary file 1 — Supplementary Information [file 41467_2026_73181_MOESM1_ESM.pdf]

Supplementary material

A High Dimensionality Approach Reveals Immunopathogenic Responses Driving Severe Pediatric Acute Respiratory Distress Syndrome

Judith Ju Ming Wong*[1,2,3], Herng Lee Tan[4], Clare Wei Tian Foo[1,3], Nicholas Kim Huat Khoo[3], Yik-Lam Cho[3], Su Li Poh[3], Martin Wasser[2,3], Sharifah Hazirah[3], Jing Yao Leong[3], Yee Hui Mok[1,2], Daryl Zhang Wei Lee[5,6], Pavanish Kumar[2,3], Joo Guan Yeo [2,3,7†], Sylvie Alonso*[5,6,8†], Salvatore Albani*[2,3,7†]

[1] Children's Intensive Care Unit, Department of Pediatric Subspecialties, KK Women's and Children's Hospital, Singapore, Singapore

[2] Duke-NUS Medical School

[3] Translational Immunology Institute, SingHealth Duke-NUS Academic Medical Centre, Singapore, Singapore

[4] Respiratory Therapy Service, Division of Allied Health Specialties, KK Women's and Children's Hospital, Singapore, Singapore

[5] Infectious Diseases Translational Research Programme, Department of Microbiology & Immunology, Yong Loo Lin School of Medicine, National University of Singapore, Singapore

[6] Immunology Programme, Life Sciences Institute, National University of Singapore, Singapore

[7] Division of Medicine, KK Women's and Children's Hospital, Singapore, Singapore

[8] National Centre for Infectious Diseases, Singapore

† These authors contributed equally to this work

Content

		pg.
1.	Figure S1: Immune cell profile from flow cytometry of deep tracheal lavage samples in PARDS and controls	*3*
2.	Figure S2: Cytokine trends in deep tracheal lavage samples from PARDS patients (original cohort) who progressed in severity after diagnosis	*4*
3.	Figure S3: IL-1β concentrations in deep tracheal lavage samples from the independent cohort of PARDS patients	*5*
4.	Figure S4: Cells from each patient sample mapped onto the UMAP	*6*
5.	Figure S5: Expression of lineage markers in single-cell RNA sequencing cell clusters and frequency of cells in each cluster	*7*
6.	Figure S6: Heatmap of the top 5 upregulated genes in each 10X cell cluster	*8*
7.	Figure S7: tSNE plots of mass cytometry derived immune cell lineage markers, myeloid cell markers and IL1β expression	*9*
8.	Figure S8: IL-1β production upon activation with TLR7 agonists R837, CL264 and CL307	*10*
9.	Figure S9: Suppression of R837 dependent IL-1β production upon priming with IFN-γ in healthy children and patients with PARDS	*11*
10.	Figure S10: Detection of IL-1β production in an *in vitro* cell culture model of $CD14^+$ monocytes using flow cytometry and western blot	*12*
11.	Figure S11: Gene ontology pathways that were up and downregulated in monocytes exposed to IFN-γ priming and TLR7 activation	*13-14*
12.	Figure S12: Overlay of gene module from *in vitro* monocytes model on PARDS comparion dataset (PRJNA971535)	*15*
13.	Figure S13: Overlay of gene module from *in vitro* monocytes model on pediatric comparion dataset samples (GSE155249)	*16*
14.	Table S1. Flow Cytometry panel for deep tracheal lavage samples from patients with PARDS and controls	*17*
15.	Table S2: Mass cytometry panel for peripheral blood mononuclear cells from patients with PARDS and controls	*18*
16.	Table S3. Flow Cytometry panel for peripheral blood mononuclear cells in *in vitro* studies	*19*
17.	Table S4: *NanoString* - Top 20 differentially expressed genes in $CD3^+$ T cell and $CD14^+$ myeloid clusters	*20*
18.	Table S5: Single-cell RNA sequencing number of cells	*21*
19.	Table S6: Single-cell RNA sequencing for deep tracheal lavage samples - Top 20 genes in T cell clusters	*22*
20.	Table S7: Single-cell RNA sequencing for deep tracheal lavage samples - Top 20 genes in myeloid clusters	*23*
21.	Table S8. Summary of single-cell RNA sequencing annotation of pulmonary cell clusters from published datasets	*24*
22.	Table S9: Forty-five unique FlowSOM cell clusters generated from mass cytometry	*25*
23.	References	*26*

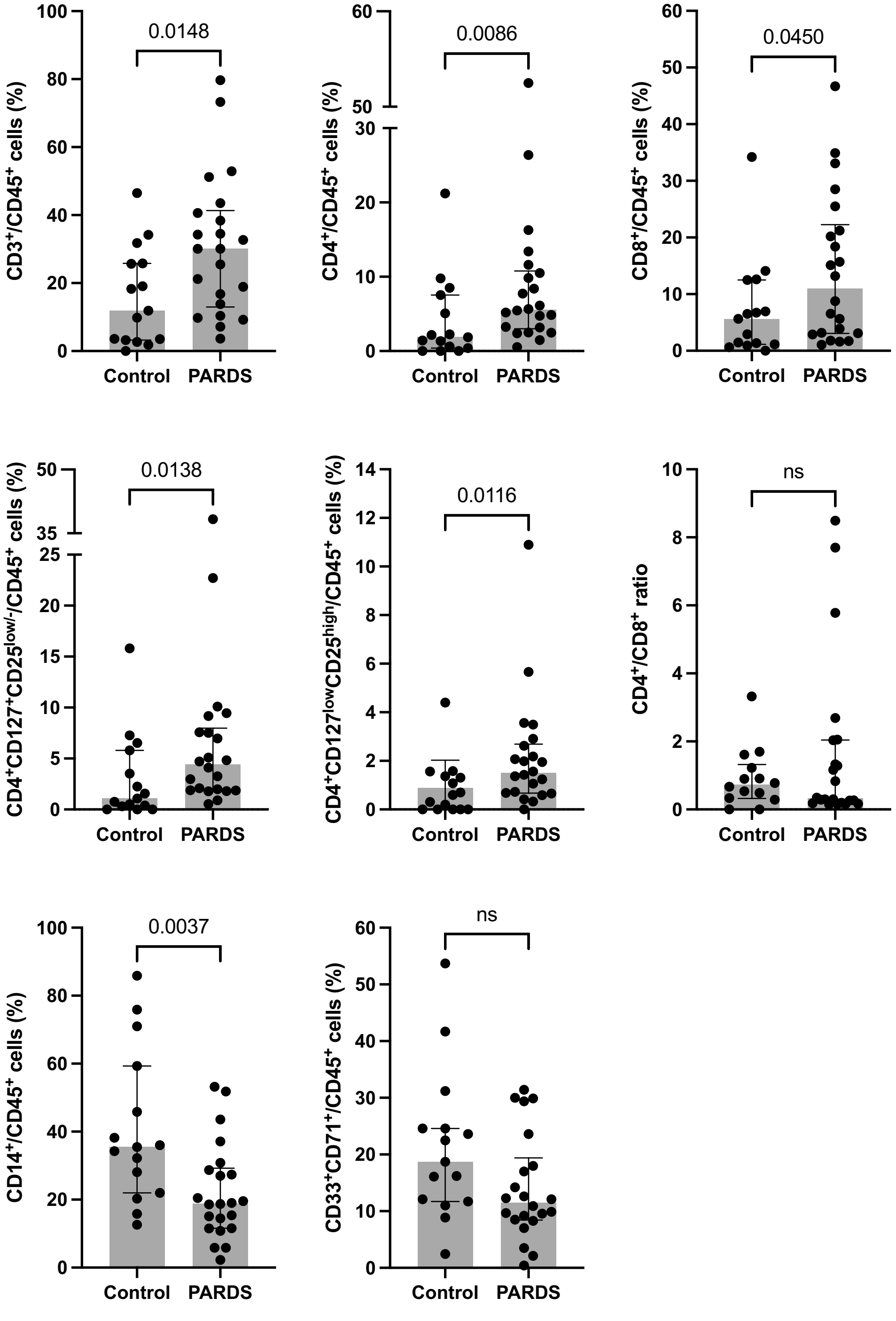

Figure S1: Immune cell profile from flow cytometry of deep tracheal lavage samples in PARDS and controls

Samples analyzed; PARDS at diagnosis, n=22 and controls, n=15. Mann Whitney-U test was used for two group comparisons of cell subset frequencies.

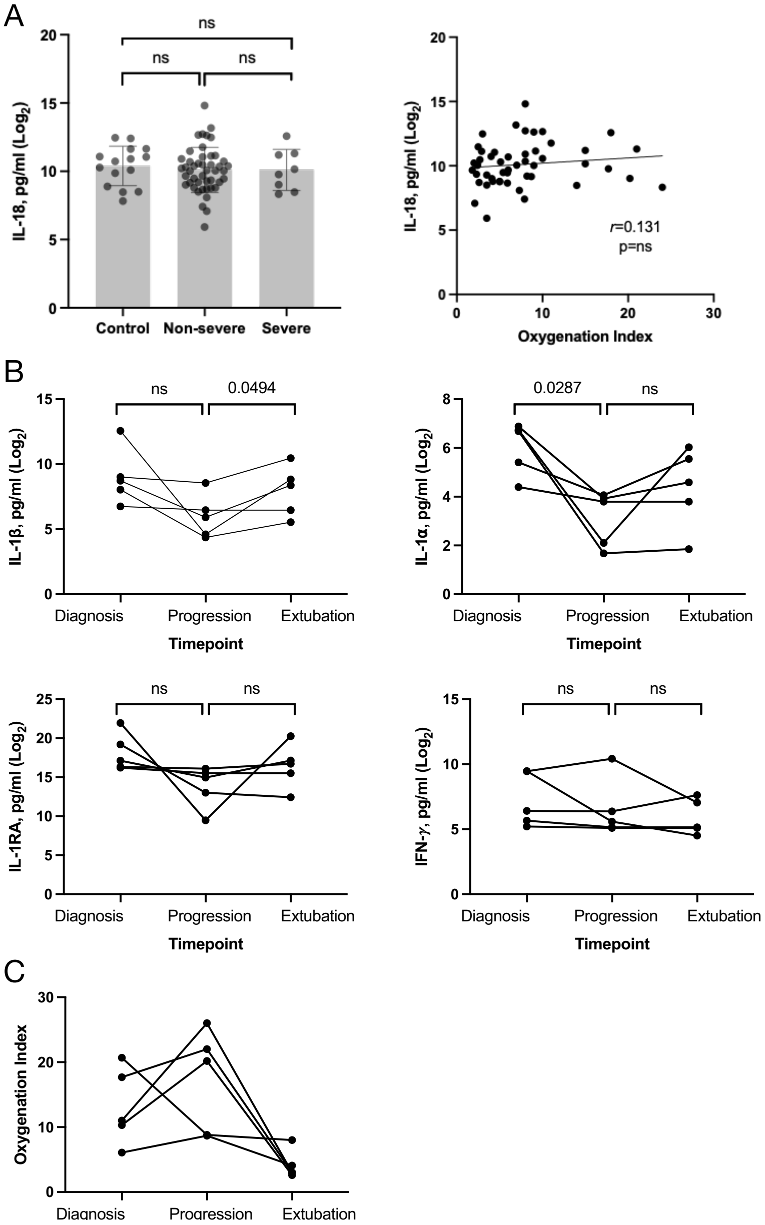

Figure S2: Cytokine trends in deep tracheal lavage samples from PARDS patients (original cohort) who progressed in severity after diagnosis

A.IL-18 concentration in the deep tracheal lavage fluid and its correlation (Pearson's) with the oxygenation index. Student's t-test was used for two group comparisons of IL-18 concentration between severe PARDS vs. control, non-severe PARDS vs. control and non-severe vs. severe PARDS. B. Samples from 5 patients with PARDS were obtained at 3 timepoints. IL-1β, IL-1α and IL-1RA levels at diagnosis, progression of disease and resolution of disease (extubation). C. Oxygenation index calculated at the point of sampling for the 5 patients. Student's t-test was used for two group comparisons of cytokine concentrations between diagnosis vs. progression and progression vs. extubation samples.

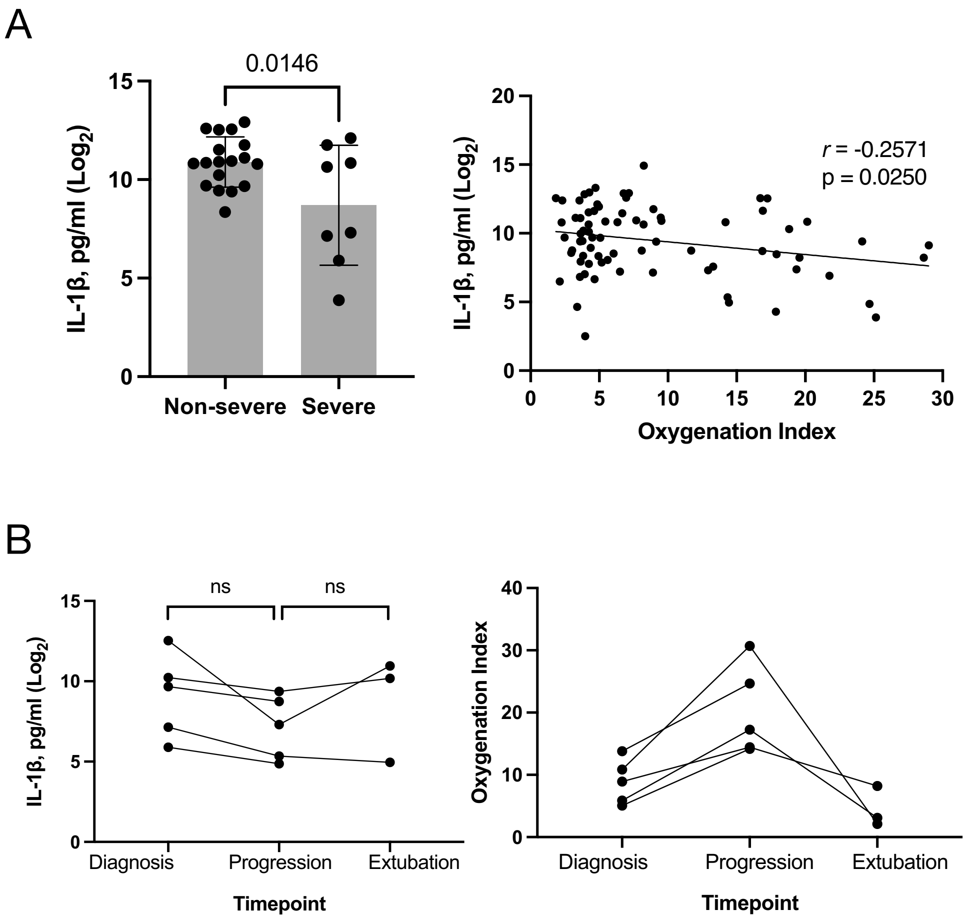

Figure S3: IL-1β concentrations in deep tracheal lavage samples from the independent cohort of PARDS patients

A.IL-1β concentrations in severe vs. non-severe PARDS and its correlation with the oxygenation index. Samples from 26 patients with PARDS were obtained at diagnosis and daily for the first 7 days of PARDS. Student's t-test comparison of IL-1β concentration between non-severe vs. severe PARDS. Pearson correlation, *r*, of IL-1β concentration and the oxygenation index. B.IL-1β at diagnosis vs. progression and progression vs. extubation samples with corresponding oxygenation index. Samples from 5 patients with PARDS were obtained at 3 timepoints. Two patients in this cohort died and do not have extubation samples. Student's t-test was used for two group comparisons.

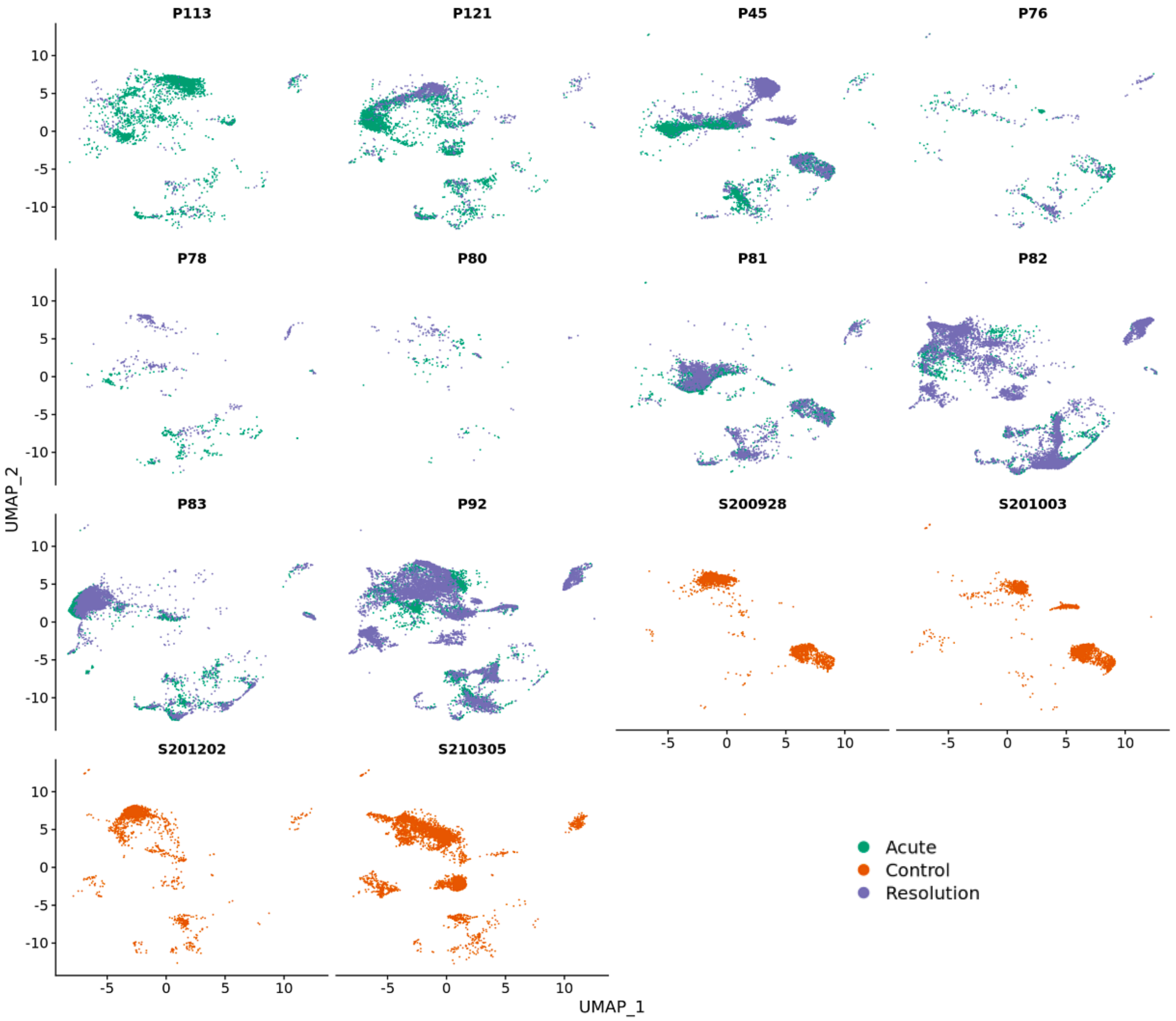

Figure S4: Cells from each patient sample mapped onto the UMAP

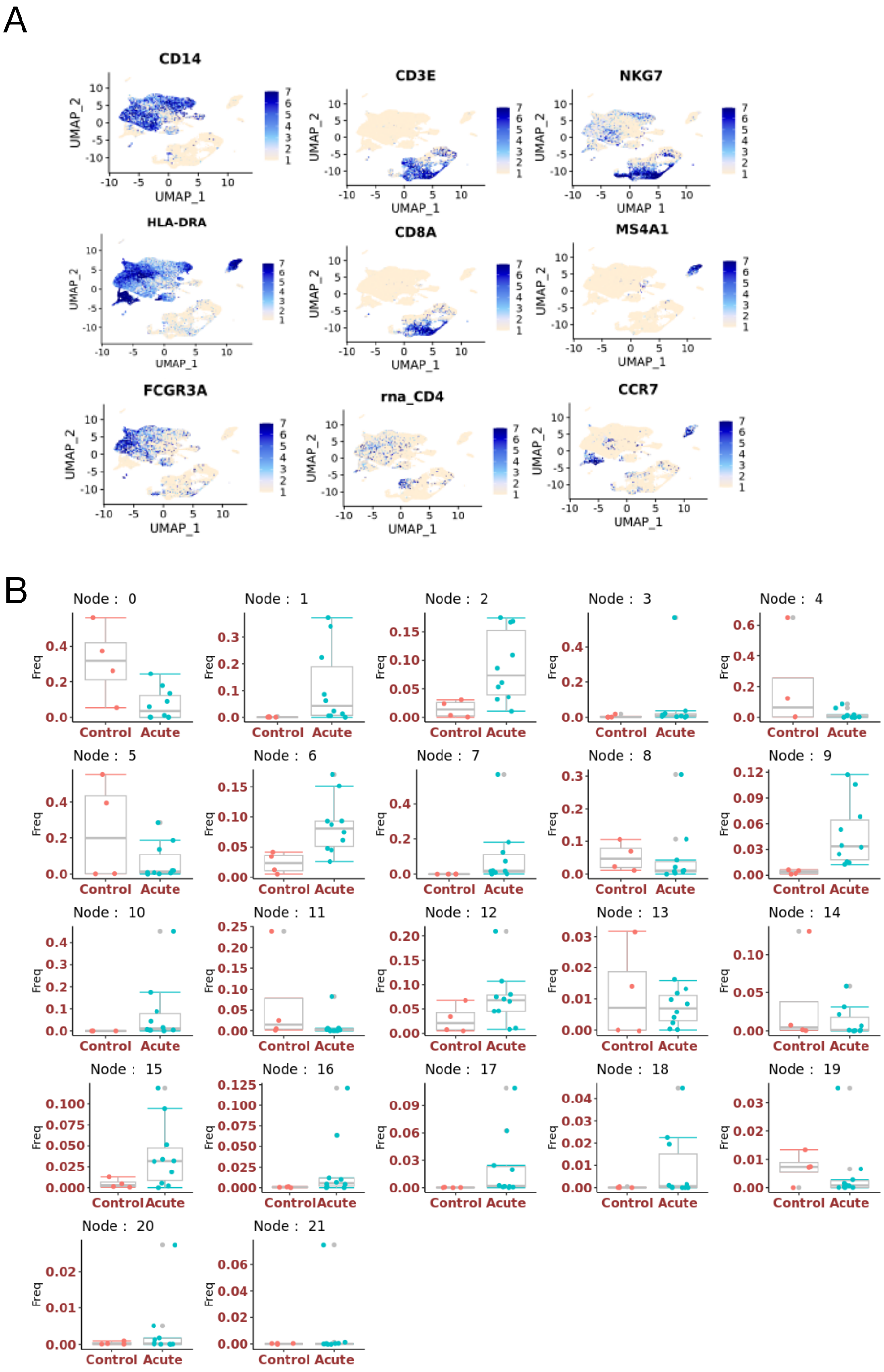

Figure S5: Expression of lineage markers in single-cell RNA sequencing cell clusters and frequency of cells in each cluster

A.Expression of lineage markers in cell clusters overlain on UMAP and B.frequency of cells in each cluster derived from single-cell RNA sequencing data

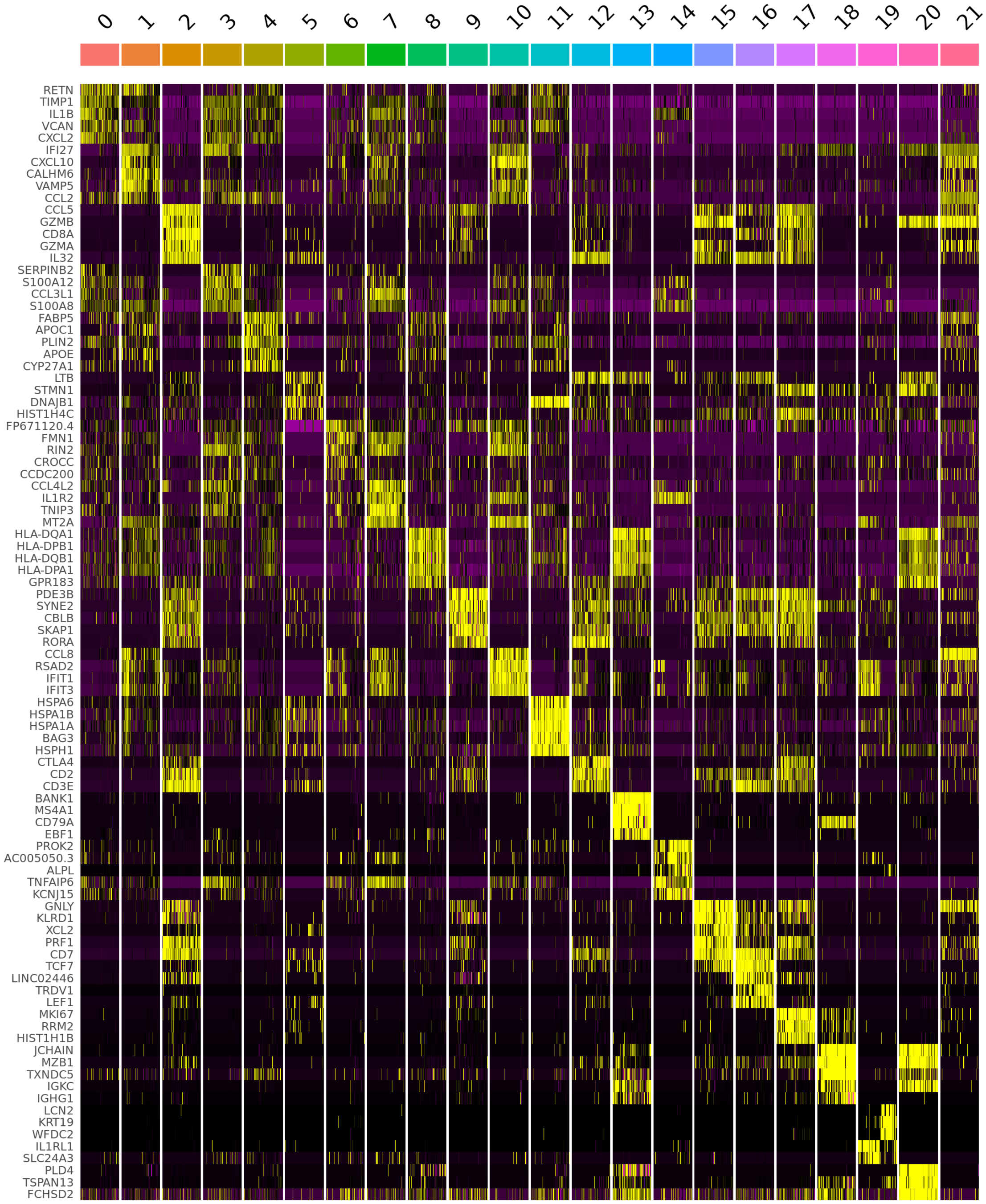

Figure S6: Heatmap of the top 5 upregulated genes in each 10X cell cluster

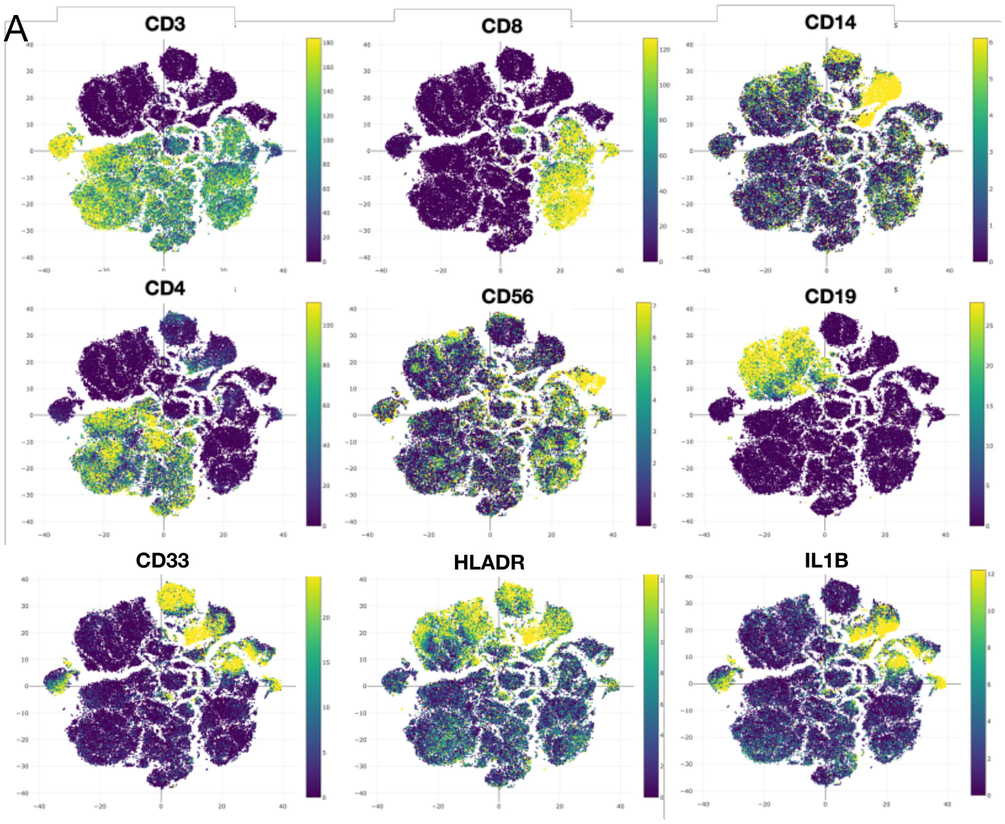

Figure S7: tSNE plots of mass cytometry derived immune cell lineage markers, myeloid cell markers and IL-1β expression

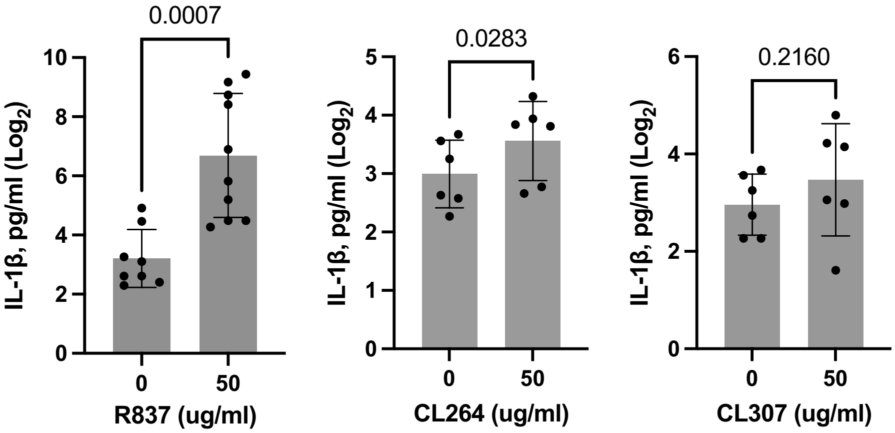

Figure S8: IL-1β production upon activation with TLR7 agonists R837, CL264 and CL307

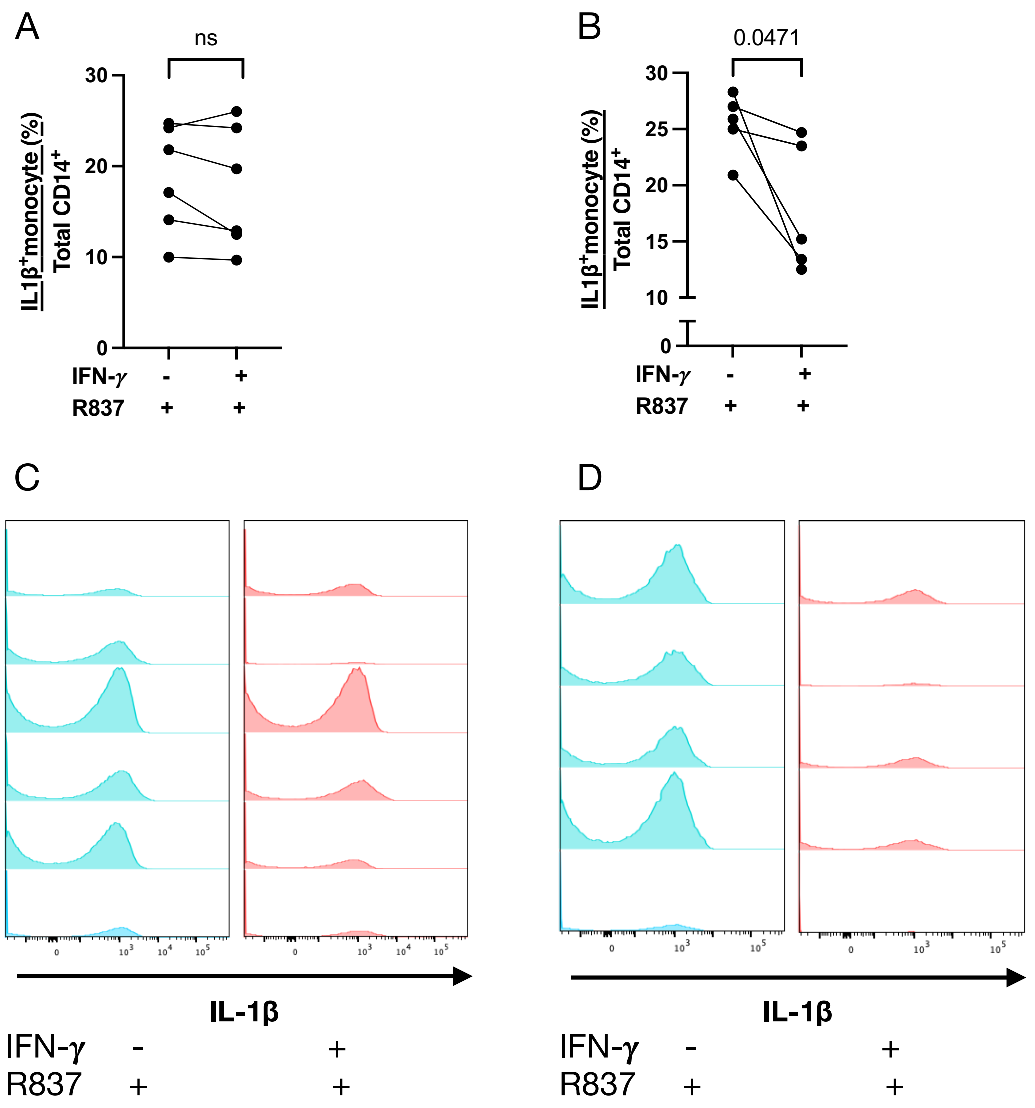

Figure S9: Suppression of R837 dependent IL-1β production upon priming with IFN-γ in healthy children and patients with PARDS

PBMCs from 5 healthy children and 6 patients with PARDS were exposed to TLR7 activation with 50μg/ml of R837 with and without 100ng/ml of IFN-γ priming. IL-1β expression was determined by flow cytometry using the BD FACSDiscover™ S8. A,Percentage IL-1β^{+} monocytes of total CD14^{+} in PARDS and B, control. C, Median fluoresnce intensity (MFI) plot of IL-1β expression in CD14^{+} monocytes in PARDS and D, control.

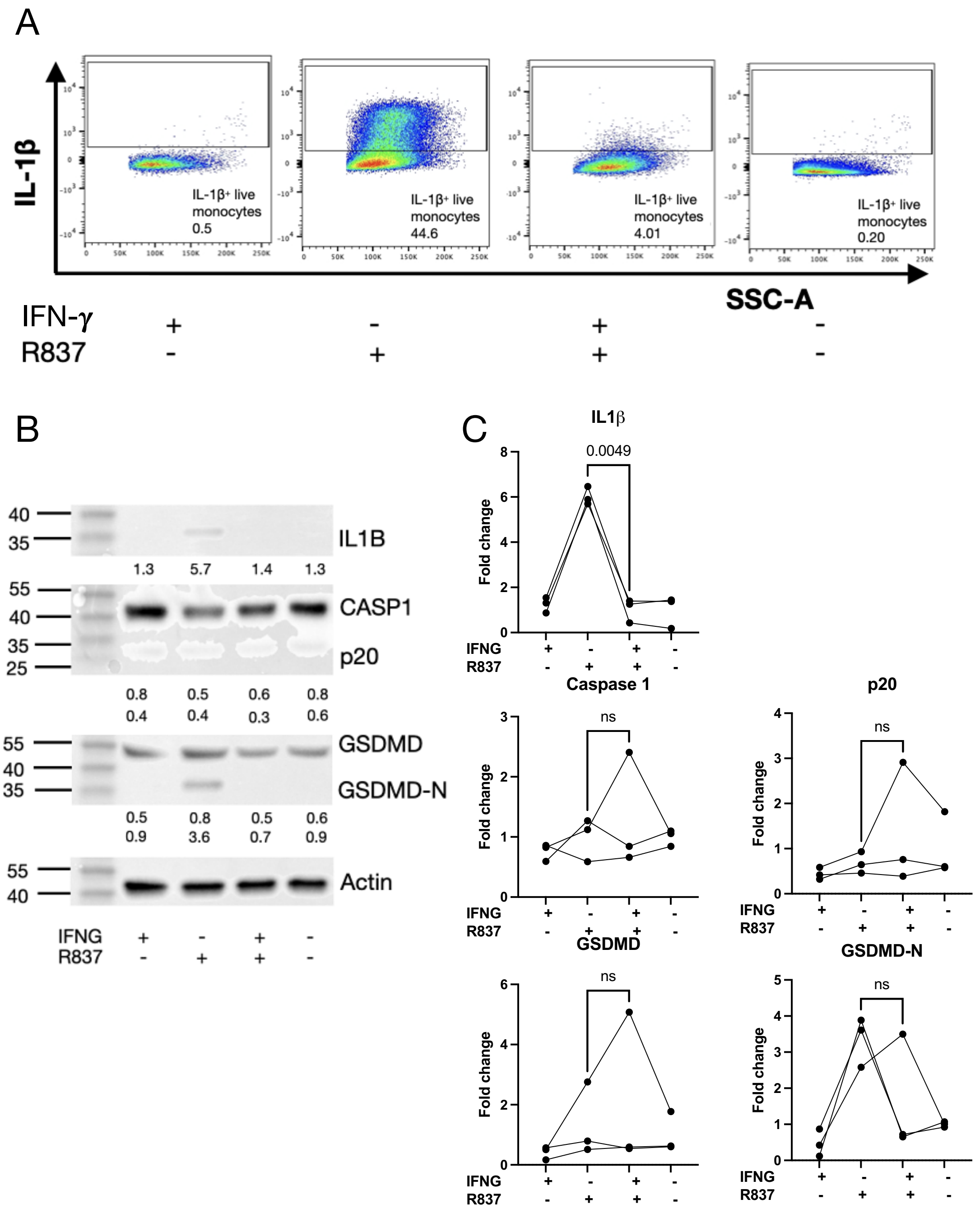

Figure S10: Detection of IL-1β production in an *in vitro* cell culture model of CD14^{+} monocytes using flow cytometry and western blot

A.Representative flow cytomnetry bivariate plot demonstrating IL-1β production in live CD14^{+} monocytes with different *in vitro* monocyte culture conditions (100ng/ml IFN-γ priming, 50ul/ml R837 stimulation, IFN-γ priming + R837 stimulation, control). B, C. Detection of IL-1β and inflammasome proteins by Western blot under indicated culture conditions (n=3 independent samples). Band intensity was measured using ImageJ version 1.53m.

A

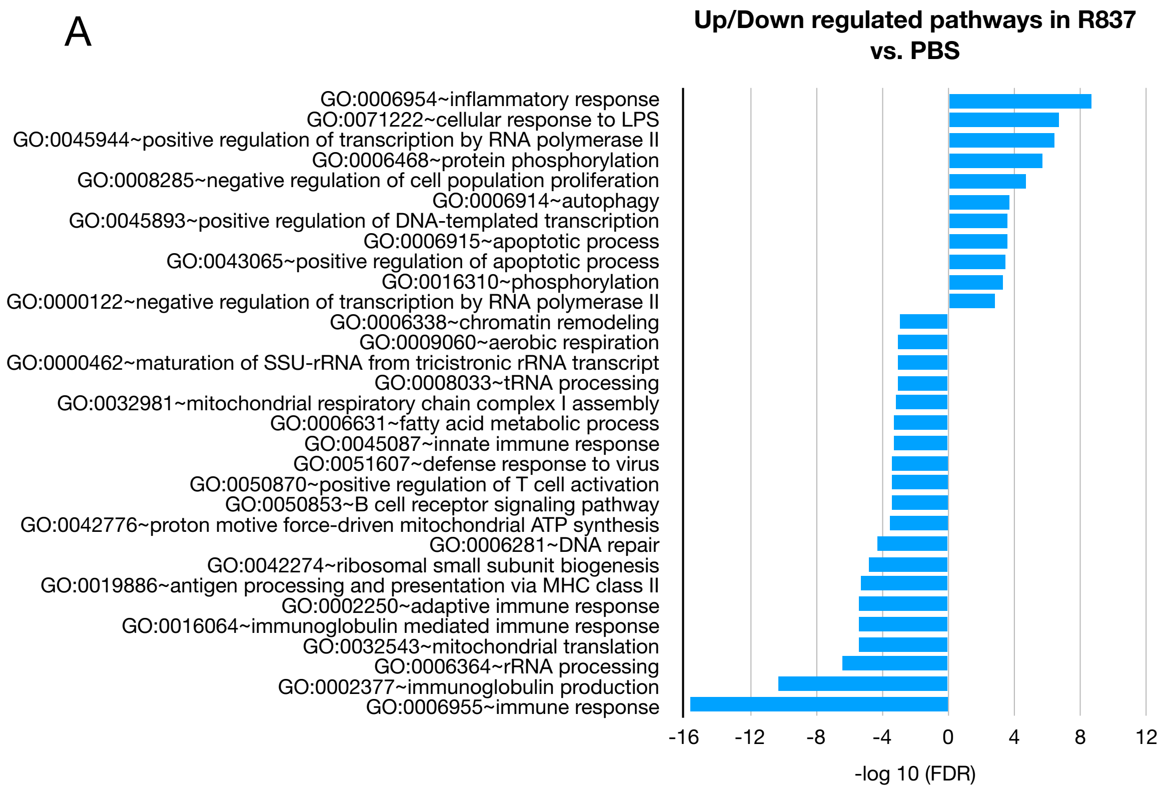
Up/Down regulated pathways in R837 vs. PBS
GO:0006954~inflammatory response
GO:0071222~cellular response to LPS
GO:0045944~positive regulation of transcription by RNA polymerase II
GO:0006468~protein phosphorylation
GO:0008285~negative regulation of cell population proliferation
GO:0006914~autophagy
GO:0045893~positive regulation of DNA-templated transcription
GO:0006915~apoptotic process
GO:0043065~positive regulation of apoptotic process
GO:0016310~phosphorylation
GO:0000122~negative regulation of transcription by RNA polymerase II
GO:0006338~chromatin remodeling
GO:0009060~aerobic respiration
GO:0000462~maturation of SSU-rRNA from tricistronic rRNA transcript
GO:0008033~tRNA processing
GO:0032981~mitochondrial respiratory chain complex I assembly
GO:0006631~fatty acid metabolic process
GO:0045087~innate immune response
GO:0051607~defense response to virus
GO:0050870~positive regulation of T cell activation
GO:0050853~B cell receptor signaling pathway
GO:0042776~proton motive force-driven mitochondrial ATP synthesis
GO:0006281~DNA repair
GO:0042274~ribosomal small subunit biogenesis
GO:0019886~antigen processing and presentation via MHC class II
GO:0002250~adaptive immune response
GO:0016064~immunoglobulin mediated immune response
GO:0032543~mitochondrial translation
GO:0006364~rRNA processing
GO:0002377~immunoglobulin production
GO:0006955~immune response
-16
-12
-8
-4
0
4
8
12
-log 10 (FDR)

B

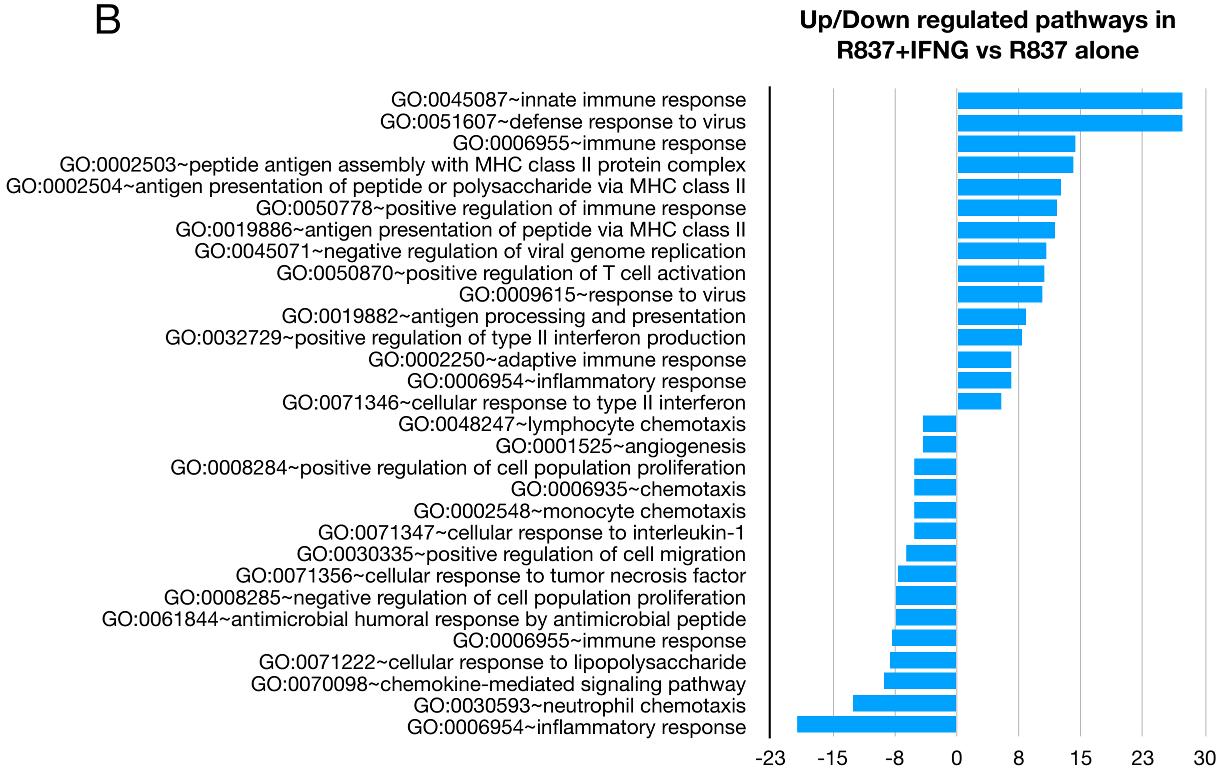
Up/Down regulated pathways in R837+IFNG vs R837 alone
GO:0045087~innate immune response
GO:0051607~defense response to virus
GO:0006955~immune response
GO:0002503~peptide antigen assembly with MHC class II protein complex
GO:0002504~antigen presentation of peptide or polysaccharide via MHC class II
GO:0050778~positive regulation of immune response
GO:0019886~antigen presentation of peptide via MHC class II
GO:0045071~negative regulation of viral genome replication
GO:0050870~positive regulation of T cell activation
GO:0009615~response to virus
GO:0019882~antigen processing and presentation
GO:0032729~positive regulation of type II interferon production
GO:0002250~adaptive immune response
GO:0006954~inflammatory response
GO:0071346~cellular response to type II interferon
GO:0048247~lymphocyte chemotaxis
GO:0001525~angiogenesis
GO:0008284~positive regulation of cell population proliferation
GO:0006935~chemotaxis
GO:0002548~monocyte chemotaxis
GO:0071347~cellular response to interleukin-1
GO:0030335~positive regulation of cell migration
GO:0071356~cellular response to tumor necrosis factor
GO:0008285~negative regulation of cell population proliferation
GO:0061844~antimicrobial humoral response by antimicrobial peptide
GO:0006955~immune response
GO:0071222~cellular response to lipopolysaccharide
GO:0070098~chemokine-mediated signaling pathway
GO:0030593~neutrophil chemotaxis
GO:0006954~inflammatory response
-23
-15
-8
0
8
15
23
30

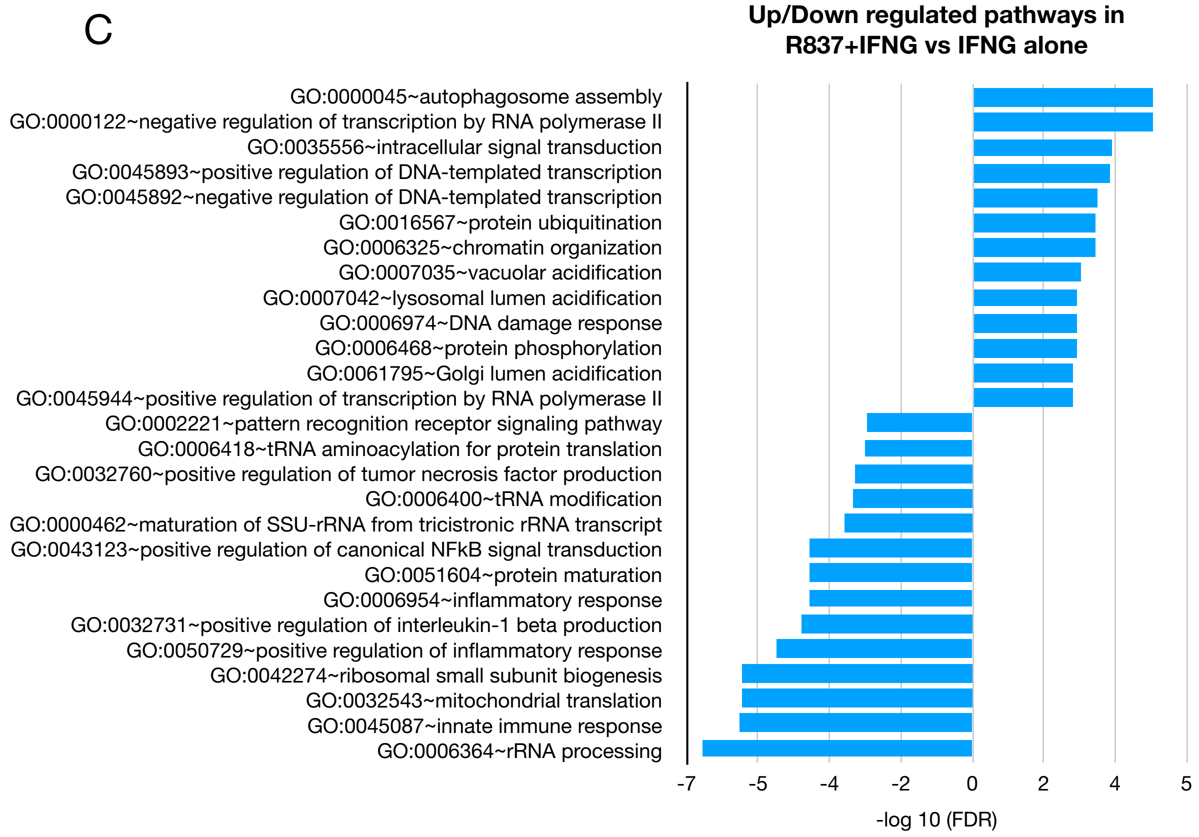

Figure S11: Gene ontology pathways that were up and downregulated in monocytes exposed to IFN-g priming and TLR7 activation

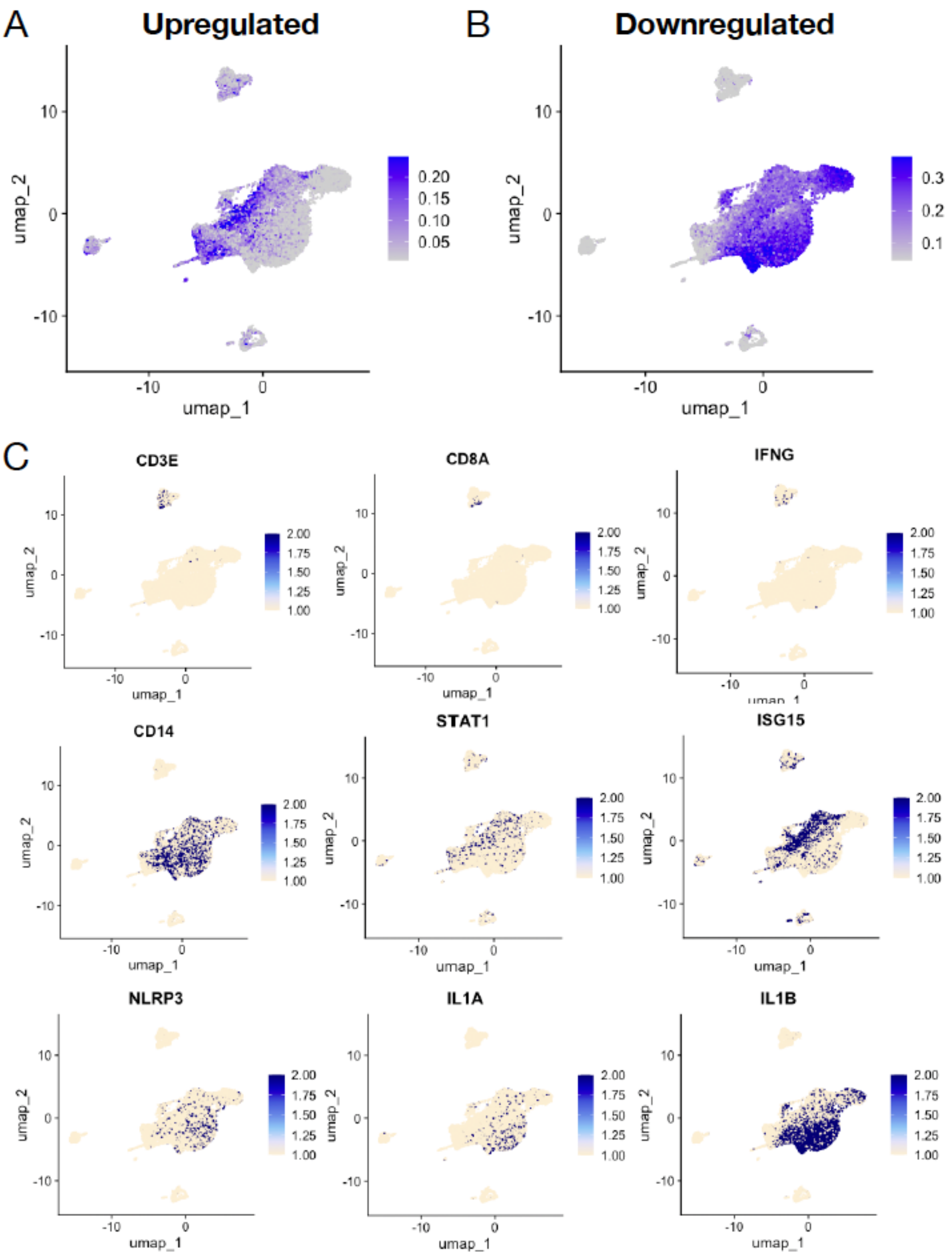

Figure S12: Overlay of gene module from *in vitro* monocytes model on PARDS comparion dataset (PRJNA971535)

Gene module derived from bulk RNA sequencing of cultured monocytes activated with 50ug/ml R837 with IFN-γ priming vs. R837 alone was applied onto the single-cell RNA sequencing PRJNA971535 dataset. A. Module score of upregulated genes and B. downregulated genes overlayed onto the UMAP. C. Individual gene expression genes overlayed onto UMAP

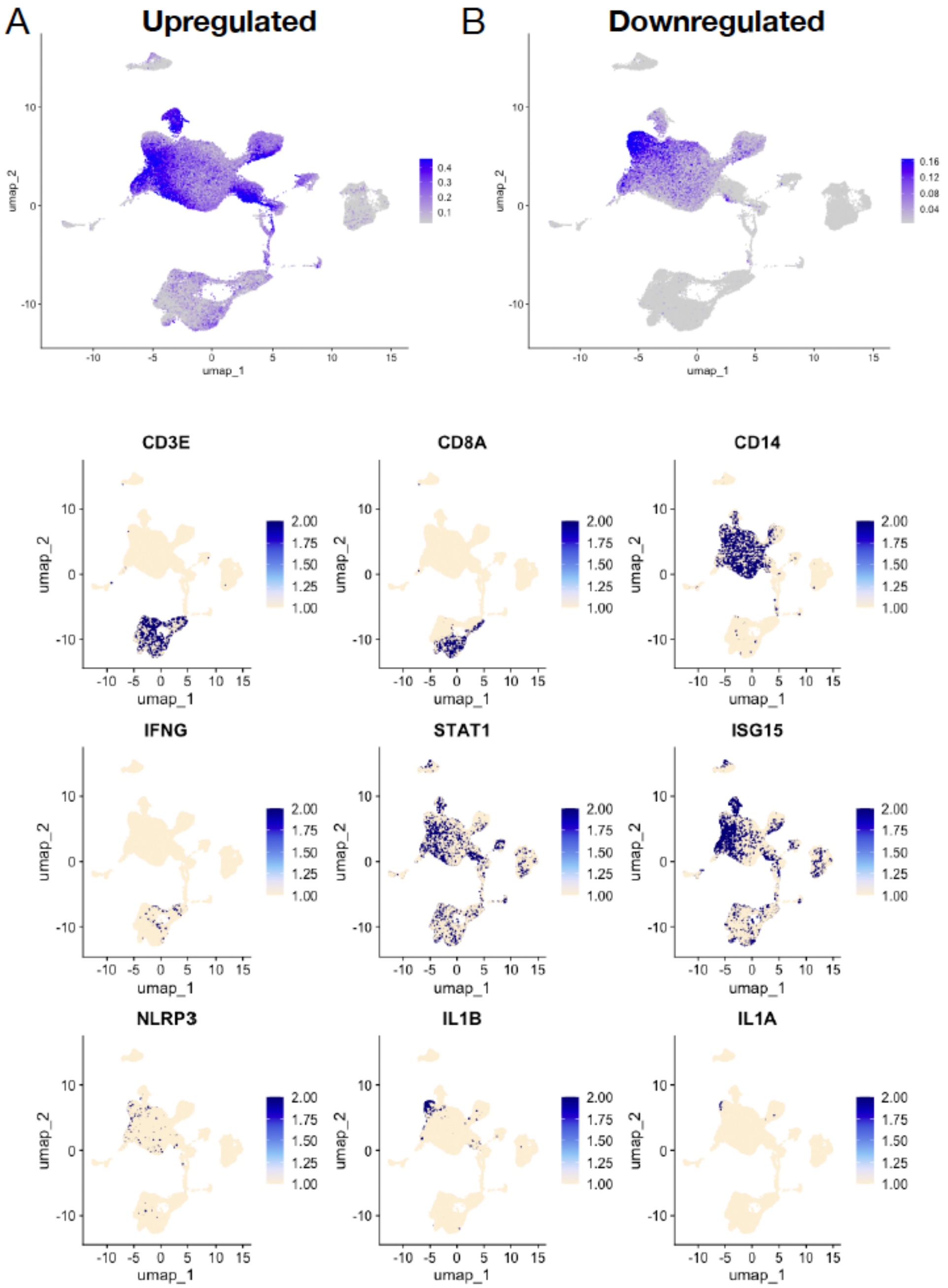

Figure S13: Overlay of gene module from *in vitro* monocytes model on pediatric comparion dataset samples (GSE155249)

Gene module derived from bulk RNA sequencing of cultured monocytes activated with 50ug/ml R837 with IFN-γ priming vs. R837 alone was applied onto the single-cell RNA sequencing GSE155249 dataset. A. Module score of upregulated genes and B. downregulated genes overlayed onto the UMAP. C. Individual gene expression genes overlayed onto UMAP

Table S1. Flow cytometry panel for deep tracheal lavage samples from patients with PARDS and controls

Marker	Clone	Cat#	Vendor	Dilution
CD45	HI30	304051	Biolegend	40
EpCAM	9C4	324217	Biolegend	40
CD3	UCHT1	300424	Biolegend	100
CD4	OKT4	317438	Biolegend	40
CD8	RPA-T8	301006	Biolegend	40
CD25	BC96	302612	Biolegend	40
CD127	A0195D	351332	Biolegend	40
CD33	P67.6	366605	Biolegend	40
CD71	CY1G4	334105	Biolegend	40
CD163	GHI/61	333607	Biolegend	40
CD14	MoP9	560180	BD	40

Table S2: Mass cytometry panel for peripheral blood mononuclear cells from patients with PARDS and controls

Metal	Antibody	Clone	Vendor
89	CD45A	HI30	Fluidigm
106	CD45B	HI30	Fluidigm
110	CD19	HIB19	Biolegend
111	CD45RA	HI100	Biolegend
112	CD14	Tuk4	Invitrogen
113	CD45C	HI30	Fluidigm
114	CD56	NCAM16.2	BD
115	CD45D	HI30	Biolegend
116	CD8	SK1	Biolegend
139	HLA-DR	L243	Biolegend
141	CD244	C1.7	Biolegend
142	CD107A	H4A3	Biolegend
143	CD3	UCHT1	Biolegend
144	IL6	MQ2-13A5	Biolegend
145	IL4	8D4-8	Biolegend
146	TCRGD_FITC	FIT-22	Biolegend
147	PD-1	EH12.2H7	Biolegend
148	CD4	SK3	Biolegend
149	IL-2	MQ1-17H12	Biolegend
150	CCR4	L291H4	Biolegend
151	GATA3	TWAJ	eBioscience
152	TNF-a	Mab11	Biolegend
153	CD25	2A3	bdbiosciences
154	CD15	H198	Biolegend
155	CD152	BNI3	BD
156	CD28	CD28.2	Biolegend
157	CXCR5	RF8B2	BD
158	CCR7	G043H7	Biolegend
159	CXCR3	G025H7	Biolegend
160	CD161	HP-3G10	Biolegend
161	CCR2	K036C2	Biolegend
162	CD160	BY55	Biolegend
163	TBX21/T-bet	4B10	BioXell
164	CX3CR1	K0124E1	Biolegend
165	FoxP3	PCH101	eBioscience
166	Ki67	20Raj1	eBioscience
167	TCRa7.2	3C10	Biolegend
168	IFN-y	B27	Biolegend
169	IL-17A	BL168	Biolegend
170	IL-8	BH0814	Biolegend
171	TIGIT	MBSA43	Invitrogen
172	IL-1β	JK1B-1	Biolegend
173	GranzymeB	ab103159	Abcam
174	CD33	WM53	Biolegend
175	MRGX2 (MasR)	K125H4	Biolegend
176	iCOS	C398.4A	Biolegend
191/193	DNA		
195	Live/Dead		
209	CD16	3G8	Fluidigm

Table S3. Flow Cytometry panel for peripheral blood mononuclear cells in *in vitro* studies

Marker	Clone	Cat#	Vendor	Dilution
CD14	M5E2	301822	Biolegend	50
CD19	HIB19	302244	Biolegend	50
CD3	SK7	344808	Biolegend	50
CD56	5.1H11	362552	Biolegend	50
IL-1β	JK1B-1	508206	Biolegend	50
IL-1α	364-3B3-14	500106	Biolegend	50
CD11c	Bu15	337218	Biolegend	50
HLA-DR	G46-6	582331	BD	50
CD1c	L161	331524	Biolegend	50
CD141	M80	344110	Biolegend	50
CD123	6H6	306022	Biolegend	50

Table S4: *NanoString* - Top 20 differentially expressed genes in CD3⁺ T cell and CD14⁺ myeloid clusters

CD3$^+$ T cells	CD14$^+$ myeloid cells	
**Top 20 upregulated genes**	**Top 20 upregulated genes**	**Top 20 downregulated genes**
MX1	ISG15	JUN
IL2RG	CXCL10	FCGR2A
LCP2	C1QB	GPR183
OAZ1	C1QA	IL1RN
CCL4	SIGLEC1	HIF1A
CTSC	CCL8	JAK3
GBP5	IFIT1	SIGLEC5
FKBP5	CEBPD	AREG
CD97	CXCL11	FCAR
IL32	PSMB9	MPEG1
GNLY	EIF2B4	CXCL5
CD81	IRF7	MARCKSL1
GZMA	MX1	ADAM8
IFI16	TM7SF3	CXCL8
CD8A	IFNB1	NLRP3
IRF7	TNFSF10	IL1B
PSMB7	CBR1	IL1RAP
KLRB1	PRDX1	IL1A
CCR5	IL3RA	SERPINB9
LAG3	CCL7	PTGS2

There were no downregulated CD3+ T cell genes meeting fold change>1.5 criteria

Table S5: Single-cell RNA sequencing number of cells

Study_ID	Status	Timepoint	Cells
P113_1_G	PARDS	1	2696
P113_2_G	PARDS	2	200
P121_1_G	PARDS	1	3164
P121_2_G	PARDS	2	1064
P45_10_GEX	PARDS	1	3656
P45_12_GEX	PARDS	2	4398
P76_26_GEX	PARDS	1	459
P76_30_GEX	PARDS	2	337
P78_1_G	PARDS	1	311
P78_2_G	PARDS	2	378
P80_1_G	PARDS	1	94
P80_2_G	PARDS	2	128
P81_3_GEX	PARDS	1	2930
P81_4_GEX	PARDS	2	2706
P82_1_G	PARDS	1	1635
P82_2_G	PARDS	2	7717
P83_21_GEX	PARDS	1	2083
P83_24_GEX	PARDS	2	3547
P92_1_G	PARDS	1	3414
P92_2_G	PARDS	2	7765
S200928_GEX	Control		2151
S201003_GEX	Control		2203
S201202_GEX	Control		1887
S210305_GEX	Control		4511
Total			59434

Timepoint 1 PARDS diagnosis
Timepoint 2 Extubation

Table S6: Single-cell RNA sequencing for deep tracheal lavage samples - Top 20 genes in T cell clusters

Cluster 2 "IFN-Cytotoxic T cell 1"	Cluster 5 "Double negative T cell"	Cluster 9 "IFN-Cytotoxic T cell 2"	Cluster 12 "T regulatory"	Cluster 16 "γδT-cells"	Cluster 17 "IFN-Cytotoxic T cell 3"
CCL5	LTB	PDE3B	IL32	CD7	MYBL2
GZMB	STMN1	SYNE2	RORA	TCF7	HIST1H3F
CD8A	HSP90AA1	CBLB	CTLA4	LINC02446	CCNA2
GZMA	CD3D	SKAP1	CD2	TRDV1	HIST1H3G
IL32	IL32	RORA	CD3E	LEF1	HIST1H3B
CD7	DNAJB1	CD96	LTB	CD3E	MKI67
PRF1	LEF1	THEMIS	SPOCK2	PTPRCAP	FAM111B
CD3E	ITM2A	PTPN22	ICOS	IKZF2	RRM2
PTPRCAP	HSPD1	PPP1R16B	PTPRCAP	CD3D	BIRC5
CD8B	HIST1H4C	CD247	ETS1	TRDC	UBE2C
LAG3	CD3E	ZEB1	CD3D	LCK	HIST1H1B
CD3D	HSPE1	INPP4B	CD247	SPOCK2	POLQ
CST7	BACH2	ANKRD36C	ITK	CD3G	ASPM
CD2	PTPRCAP	ANK3	LAG3	CXCR3	DIAPH3
NKG7	ID3	PGAP1	TNFRSF4	TRBC1	NUSAP1
GZMK	CAMK4	ABLIM1	CD69	ETS1	HIST1H2AJ
CD96	TYMS	CAMK4	PIM2	TRGV4	TPX2
IFNG	ETS1	PRF1	CD7	TRDV3	NCALD
LCK	SOX4	CCL5	SYNE2	IL32	PKMYT1
GZMH	CD69	TTN	KLRB1	SAMD3	HIST1H3C

Tale S7: Single-cell RNA sequencing for deep tracheal lavage samples - Top 20 genes in myeloid clusters

Cluster 0 “Inflammatory monocyte”	Cluster 1 “IFN related MoAM”	Cluster 3 “Inflammatory MoAM”	Cluster 4 “Mature AM”
RETN	IFI27	SERPINB2	FABP5
TIMP1	CXCL10	IFI27	APOC1
IL1B	CALHM6	S100A12	PLIN2
VCAN	VAMP5	CCL3L1	APOE
CXCL2	CCL2	S100A8	CYP27A1
CXCL8	CCL8	RNASE2	CD36
CXCL3	WARS	TNFAIP6	MARCO
S100A12	APOBEC3A	S100A9	FBP1
S100A9	GBP1	SLC39A8	CTSD
G0S2	SERPING1	CCL7	CSTB
PID1	IFITM3	RIN2	MMP19
S100A8	IFI6	CCL4L2	GPNMB
PHLDA1	FCGR3A	APOBEC3A	CTSL
LYZ	LYZ	LYZ	CD163
FCN1	MT2A	CXCL3	EGR1
IL1RN	GLUL	IL1B	VSIG4
MMP19	LGALS3BP	MIR3945HG	CD9
MCEMP1	CD14	CXCL2	LGALS3
OLR1	ANKRD22	CXCL8	MRC1
LUCAT1	GBP5	CCL3	FABP4
**Cluster 6 “Indeterminate”**	**Cluster 7 “Inflammatory AM”**	**Cluster 8 “DCs”**	**Cluster 10 “IFN related AM”**
FP671120.4	CCL4L2	HLA-DQA1	CXCL10
FMN1	CCL3L1	HLA-DPB1	CCL8
RIN2	IL1R2	HLA-DQB1	RSAD2
CEMIP	TNIP3	HLA-DPA1	IFIT1
SLC8A1	MT2A	GPR183	IFIT3
CROCC	TNFAIP6	HLA-DRB5	ISG15
ADAMTS2	IL6	HLA-DRA	DEFB1
IL1R2	G0S2	NR4A3	IFIT2
MCTP1	CCL20	HLA-DRB1	MT2A
AL163541.1	FMN1	RGS1	APOBEC3A
CCDC200	PLAC8	CCR7	TNFSF10
SLC39A8	MIR3945HG	CD86	CCL2
EXT1	CCL4	GRASP	HERC5
AZIN1-AS1	SLC1A3	CD74	MX1
ALDH1A2	IL18	AREG	CXCL11
TPST1	IL1RN	CST7	DDX58
PLD1	EBI3	ALCAM	IFI27
PELI1	CCL8	GSN	RIN2
PRR16	C15orf48	RFTN1	CCL7
MGLL	IL1B	SLC7A11	NCF1

Table S8: Summary of single-cell RNA sequencing annotation of pulmonary cell clusters from published datasets

Myeloid cluster	Marker genes	Tissue/disease	Literature/ population
Cluster 0:<br>Inflammatory monocyte	IL1B<br>S100A12<br>S100A9<br>S100A8<br>CD14	BAL/AHRF<br>BAL/COVID-19<br>BAL/Healthy<br>Lung tissue/ resected lung tumor	"Inflammatory monocytes"[1]<br>"Monocyte-IL1B"[2]<br>"Monocyte" [3]<br>"Classical monocyte" [4]
Cluster 1:<br>IFN related MoAM	IFI27<br>CXCL10<br>CXCL9<br>IFITM3	BAL/Healthy and CF	"Chemokine AM" [5]
Cluster 3:<br>Inflammatory MoAM	SERPINB2<br>S100A12<br>S100A8<br>TNFAIP6<br>S100A9<br>IL1B<br>CXCL8	Not previously defined	
Cluster 4:<br>Mature AM	FABP4<br>MARCO<br>APOE<br>CD163<br>MRC1<br>MSR1	BAL/COVID-19<br>BAL/AHRF<br>BAL/Healthy<br>Lung tissue/ resected lung tumor	"Tissue resident AM"[2]<br>"Mature AM" [1]<br>"Macrophage" [3]<br>"Macrophage" [4]
Cluster 6:<br>Indeterminate	FP671120.4<br>FMN1<br>RIN2<br>CEMIP<br>SLC8A1	Not previously defined	
Cluster 7:<br>Inflammatory AM	CCL4L2<br>CCL3L1<br>IL6	Not previously defined	
Cluster 8:<br>Dendritic cell	CD1C<br>HLA-DR<br>CLEC10A	BAL/AHRF<br>AL/Healthy	"Dendritic cell" [1]<br>"cDC2" [3]
Cluster 10:<br>IFN related AM	CXCL10<br>IFIT1<br>IFIT3<br>ISG15<br>DEFB1<br>MX1	BAL/AHRF<br>BAL/Healthy	"IFN-related macrophage" [1]<br>"m5" [3]

Table S9: Forty-five unique FlowSOM cell clusters generated from mass cytometry

Lineage	Subset	Subset number
CD4 T cells	$CD152^+$ central memory CD4	2
	$CD161^+TNFA^+IL17A^+PD1^+CD152^+Ki67^+$ effector memory CD4	4
	$CD161^+TNFA^+IL17A^+$ effector memory CD4	5
	$CD161^+$ effector memory CD4	6
	$CD25^+$ effector memory CD4	7
	$CXCR3^+CX3CR1^+$ naïve CD4	10
	$IL2^+$ naïve CD4	17
	$IL4^+$ naïve CD4	18
	$IL6^+$ naïve CD4	19
	$IL8^+$ naïve CD4	21
	$PD1^+CD152^+CD161^+$ effector memory CD4	26
	$PD1^+CD152^+Ki67^+$ effector memory CD4	27
	$PD1^+CD152^+TIGIT^+$ effector memory CD4	28
	$TNFA^+IFG\gamma^+$ effector memory CD4	30
	$TNFA^+IL2^+$ effector memory CD4	31
	Central memory CD4	36
	Effector memory CD4	37
	Memory Treg	38
	Naïve CD4	40
	Naïve Treg	42
	Temra CD4	43
CD8 T cells	$GB^+IFN\gamma^+$ effector memory CD8	13
	$GB^+IFN\gamma^+$ temraCD8	14
	GB^+ temra CD8	15
	$IFN\gamma^+$ effector memory CD8	16
	$IL8^+$ naïve CD8	22
	$Tbet^+GB^+IFN\gamma^+Ki67^+$ effector memory CD8	32
	$Tbet^+GB^+Ki67^+$ effector memory CD8	33
	Naïve CD8	41
	Temra CD8	44
Other T cells	$Tbet^+GB^+$ $\gamma\delta$T	34
	$\gamma\delta$T	35
	Double negative T cell	12
	$TBET^+CD161^+TNFA^+IFN\gamma^+$ double negative T cell	29
B cell	B	1
	$CXCR5^-Ki67^+$ B cell	11
Myeloid	$CD15^+$ $IL1\beta^-$ monocyte	3
	$CXCR3^+CX3CR1^+$ monocyte	9
	Monocyte	39
	$IL8^+IL1\beta^+$ myeloid cell	20
	Myeloid cell	24
NK cell	$CD56^{hi}$ NK cell	8
	NK	25
Others	ILC2	23
	Undefined	45

References:

1. Morrell ED, Holton SE, Lawrance M *et al*. The transcriptional and phenotypic characteristics that define alveolar macrophage subsets in acute hypoxemic respiratory failure. Nature communications 2023; 14(1):7443. 10.1038/s41467-023-43223-0
2. Wauters E, Van Mol P, Garg AD *et al*. Discriminating mild from critical COVID-19 by innate and adaptive immune single-cell profiling of bronchoalveolar lavages. Cell Research 2021; 31(3):272-290. 10.1038/s41422-020-00455-9
3. Mould KJ, Moore CM, McManus SA *et al*. Airspace Macrophages and Monocytes Exist in Transcriptionally Distinct Subsets in Healthy Adults. American journal of respiratory and critical care medicine 2021; 203(8):946-956. 10.1164/rccm.202005-1989OC
4. Travaglini KJ, Nabhan AN, Penland L *et al*. A molecular cell atlas of the human lung from single-cell RNA sequencing. Nature 2020; 587(7835):619-625. 10.1038/s41586-020-2922-4
5. Li X, Kolling FW, Aridgides D *et al*. ScRNA-seq expression of IFI27 and APOC2 identifies four alveolar macrophage superclusters in healthy BALF. Life Sci Alliance 2022; 5(11). 10.26508/lsa.202201458
