# Supplementary material for: A high dimensionality approach reveals immunopathogenic responses driving severe pediatric acute respiratory distress syndrome
Source: Nat Commun. 2026 May 15;17:6466. doi: 10.1038/s41467-026-73181-2 (PMC13376821; doi:10.1038/s41467-026-73181-2)
Supplement: Supplementary file 2 — Reporting Summary [file 41467_2026_73181_MOESM2_ESM.pdf]

nature portfolio

Corresponding author(s): Judith Ju Ming Wong

Last updated by author(s): 6th April 2026

Reporting Summary

Nature Portfolio wishes to improve the reproducibility of the work that we publish. This form provides structure for consistency and transparency in reporting. For further information on Nature Portfolio policies, see our Editorial Policies and the Editorial Policy Checklist.

Please do not complete any field with "not applicable" or n/a. Refer to the help text for what text to use if an item is not relevant to your study. For final submission: please carefully check your responses for accuracy; you will not be able to make changes later.

Statistics

For all statistical analyses, confirm that the following items are present in the figure legend, table legend, main text, or Methods section.

n/a	Confirmed	
☐	☑	The exact sample size (*n*) for each experimental group/condition, given as a discrete number and unit of measurement
☐	☑	A statement on whether measurements were taken from distinct samples or whether the same sample was measured repeatedly
☐	☑	The statistical test(s) used AND whether they are one- or two-sided<br>*Only common tests should be described solely by name; describe more complex techniques in the Methods section.*
☑	☐	A description of all covariates tested
☐	☑	A description of any assumptions or corrections, such as tests of normality and adjustment for multiple comparisons
☐	☑	A full description of the statistical parameters including central tendency (e.g. means) or other basic estimates (e.g. regression coefficient) AND variation (e.g. standard deviation) or associated estimates of uncertainty (e.g. confidence intervals)
☐	☑	For null hypothesis testing, the test statistic (e.g. *F*, *t*, *r*) with confidence intervals, effect sizes, degrees of freedom and *P* value noted<br>*Give P values as exact values whenever suitable.*
☑	☐	For Bayesian analysis, information on the choice of priors and Markov chain Monte Carlo settings
☑	☐	For hierarchical and complex designs, identification of the appropriate level for tests and full reporting of outcomes
☐	☑	Estimates of effect sizes (e.g. Cohen's *d*, Pearson's *r*), indicating how they were calculated

Our web collection on statistics for biologists contains articles on many of the points above.

Software and code

Policy information about availability of computer code

Data collection	Becton Dickinson (BD) FACSDiva and FACSChorus software (flow cytometry), CyTOF software Standard BioTools (mass cytometry), Perkin Elmer EnSpire 2300 (ELISA, colorimetry), ChemiDoc MP Imaging System Bio-Rad (western blot).
Data analysis	FlowJo v10.10.1 (flow/ mass cytometry); nSolver v4.0 (Nanostring); CellRanger, Seurat, SCTransform, Velocyto, scvelo, scanpy, ModuleScore (single-cell RNA sequencing); ImageJ verion v1.53m (western blot), GraphPad PRISM v11.0.0

For manuscripts utilizing custom algorithms or software that are central to the research but not yet described in published literature, software must be made available to editors and reviewers. We strongly encourage code deposition in a community repository (e.g. GitHub). See the Nature Portfolio guidelines for submitting code & software for further information.

Data

Policy information about availability of data

All manuscripts must include a data availability statement. This statement should provide the following information, where applicable:

- Accession codes, unique identifiers, or web links for publicly available datasets
- A description of any restrictions on data availability
- For clinical datasets or third party data, please ensure that the statement adheres to our policy

The raw count data generated in this study have been deposited in the GEO public repository under accession code GSE327171.
The processed data used to produce figures are available in the Zenodo repository (https://doi.org/10.5281/zenodo.18843576).
Associated data generated in this study are provided in the Supplementary Information/Source Data file.

Research involving human participants, their data, or biological material

Policy information about studies with human participants or human data. See also policy information about sex, gender (identity/presentation), and sexual orientation and race, ethnicity and racism.

Reporting on sex and gender	No sex and gender-based analyses were performed.
Reporting on race, ethnicity, or other socially relevant groupings	All subjects were Asian and no race or ethnicity based analyses were performed.
Population characteristics	All subjects were children; had PARDS in the disease group or were ICU (no lung disease)/ healthy controls.
Recruitment	All subjects were recruited to the study based on clinical criteria from a single center.
Ethics oversight	SingHealth Centralised Institutional Review Board (CIRB ref. no. 2017-3076, 2015-2231, 2019-2961 and 2018-2676). Patients recruited under CIRB 2019-2961 received transport reimbursement.

Note that full information on the approval of the study protocol must also be provided in the manuscript.

Field-specific reporting

Please select the one below that is the best fit for your research. If you are not sure, read the appropriate sections before making your selection.

☑ Life sciences ☐ Behavioural & social sciences ☐ Ecological, evolutionary & environmental sciences

For a reference copy of the document with all sections, see nature.com/documents/nr-reporting-summary-flat.pdf

Life sciences study design

All studies must disclose on these points even when the disclosure is negative.

Sample size	No statistical approach was used to pre-determine the sample size
Data exclusions	No data were excluded
Replication	Findings in this study were reproduced in an independent cohort of PARDS patients.
Randomization	Not applicable
Blinding	Not applicable

Behavioural & social sciences study design

All studies must disclose on these points even when the disclosure is negative.

Study description	
Research sample	
Sampling strategy	
Data collection	
Timing	
Data exclusions	
Non-participation	
Randomization	

Ecological, evolutionary & environmental sciences study design

All studies must disclose on these points even when the disclosure is negative.

Study description	
Research sample	
Sampling strategy	
Data collection	
Timing and spatial scale	
Data exclusions	
Reproducibility	
Randomization	
Blinding	

Did the study involve field work? ☐ Yes ☐ No

Field work, collection and transport

Field conditions	
Location	
Access & import/export	
Disturbance	

Reporting for specific materials, systems and methods

We require information from authors about some types of materials, experimental systems and methods used in many studies. Here, indicate whether each material, system or method listed is relevant to your study. If you are not sure if a list item applies to your research, read the appropriate section before selecting a response.

Materials & experimental systems

n/a	Involved in the study	
☐	☑	Antibodies
☑	☐	Eukaryotic cell lines
☑	☐	Palaeontology and archaeology
☑	☐	Animals and other organisms
☐	☑	Clinical data
☑	☐	Dual use research of concern
☑	☐	Plants

Methods

n/a	Involved in the study	
☑	☐	ChIP-seq
☐	☑	Flow cytometry
☑	☐	MRI-based neuroimaging

Antibodies

Antibodies used	Full list of antibodies used for flow cytometry is found in Table S1 & S3, mass cytometry inTable S2 and Western blot primary and secondary antibodies in Methods section under SDS-PAGE and Western blot section.
Validation	All antibodies are commercially available and validated.

Eukaryotic cell lines

Policy information about cell lines and Sex and Gender in Research

Cell line source(s)	
Authentication	
Mycoplasma contamination	
Commonly misidentified lines (See ICLAC register)	

Palaeontology and Archaeology

Specimen provenance	
Specimen deposition	
Dating methods	

☐ Tick this box to confirm that the raw and calibrated dates are available in the paper or in Supplementary Information.

Ethics oversight	

Note that full information on the approval of the study protocol must also be provided in the manuscript.

Animals and other research organisms

Policy information about studies involving animals; ARRIVE guidelines recommended for reporting animal research, and Sex and Gender in Research

Laboratory animals	
Wild animals	
Reporting on sex	
Field-collected samples	
Ethics oversight	

Note that full information on the approval of the study protocol must also be provided in the manuscript.

Clinical data

Policy information about clinical studies

All manuscripts should comply with the ICMJE guidelines for publication of clinical research and a completed CONSORT checklist must be included with all submissions.

Clinical trial registration	Not applicable
Study protocol	Not applicable
Data collection	Discovery cohort January 2018 to August 2023, validation cohort September 2023 to December 2024.
Outcomes	PARDS severity

Dual use research of concern

Policy information about dual use research of concern

Hazards

Could the accidental, deliberate or reckless misuse of agents or technologies generated in the work, or the application of information presented in the manuscript, pose a threat to:

No | Yes
- [x] No [ ] Yes Public health
- [x] No [ ] Yes National security
- [x] No [ ] Yes Crops and/or livestock
- [x] No [ ] Yes Ecosystems
- [x] No [ ] Yes Any other significant area

Experiments of concern

Does the work involve any of these experiments of concern:

No | Yes
- [x] No [ ] Yes Demonstrate how to render a vaccine ineffective
- [x] No [ ] Yes Confer resistance to therapeutically useful antibiotics or antiviral agents
- [x] No [ ] Yes Enhance the virulence of a pathogen or render a nonpathogen virulent
- [x] No [ ] Yes Increase transmissibility of a pathogen
- [x] No [ ] Yes Alter the host range of a pathogen
- [x] No [ ] Yes Enable evasion of diagnostic/detection modalities
- [x] No [ ] Yes Enable the weaponization of a biological agent or toxin
- [x] No [ ] Yes Any other potentially harmful combination of experiments and agents

Plants

Seed stocks

Novel plant genotypes

Authentication

ChIP-seq

Data deposition

- [ ] Confirm that both raw and final processed data have been deposited in a public database such as GEO.
- [ ] Confirm that you have deposited or provided access to graph files (e.g. BED files) for the called peaks.

Data access links
May remain private before publication.

Files in database submission

Genome browser session
(e.g. UCSC)

Methodology

Replicates

Sequencing depth

Antibodies

Peak calling parameters

Data quality

Software

Flow Cytometry

Plots

Confirm that:

☑ The axis labels state the marker and fluorochrome used (e.g. CD4-FITC).

☑ The axis scales are clearly visible. Include numbers along axes only for bottom left plot of group (a 'group' is an analysis of identical markers).

☑ All plots are contour plots with outliers or pseudocolor plots.

☑ A numerical value for number of cells or percentage (with statistics) is provided.

Methodology

Sample preparation	Sample preparation listed in Methods
Instrument	BD FACSAria II SORP (Becton Dickinson) and BD FACSDiscover™ S8
Software	FlowJo software 10.10.1
Cell population abundance	Cells were sorted based on CD3, CD14 or CD45 surface expression using fluorescence activated cell sorting
Gating strategy	Cells were gated into live CD3, CD4, CD8, CD4+CD127+CD25-, CD4+CD127-CD25+, CD14, CD14+CD33+CD71+

☑ Tick this box to confirm that a figure exemplifying the gating strategy is provided in the Supplementary Information.

Magnetic resonance imaging

Experimental design

Design type

Design specifications

Behavioral performance measures

Imaging type(s)

Field strength

Sequence & imaging parameters

Area of acquisition

Diffusion MRI ☐ Used ☐ Not used

Preprocessing

Preprocessing software

Normalization

Normalization template

Noise and artifact removal

Volume censoring

Statistical modeling & inference

Model type and settings

Effect(s) tested

Specify type of analysis: ☐ Whole brain ☐ ROI-based ☐ Both

Statistic type for inference

(See Eklund et al. 2016)

Correction

Models & analysis

n/a	Involved in the study
☐	☐ Functional and/or effective connectivity
☐	☐ Graph analysis
☐	☐ Multivariate modeling or predictive analysis

Functional and/or effective connectivity

Graph analysis

Multivariate modeling and predictive analysis

This checklist template is licensed under a Creative Commons Attribution 4.0 International License, which permits use, sharing, adaptation, distribution and reproduction in any medium or format, as long as you give appropriate credit to the original author(s) and the source, provide a link to the Creative Commons license, and indicate if changes were made. The images or other third party material in this article are included in the article's Creative Commons license, unless indicated otherwise in a credit line to the material. If material is not included in the article's Creative Commons license and your intended use is not permitted by statutory regulation or exceeds the permitted use, you will need to obtain permission directly from the copyright holder. To view a copy of this license, visit http://creativecommons.org/licenses/by/4.0/
