# Supplementary material for: A high dimensionality approach reveals immunopathogenic responses driving severe pediatric acute respiratory distress syndrome
Source: Nat Commun. 2026 May 15;17:6466. doi: 10.1038/s41467-026-73181-2 (PMC13376821; doi:10.1038/s41467-026-73181-2)
Supplement: Supplementary file 3 — Transparent Peer Review file [file 41467_2026_73181_MOESM3_ESM.pdf]

nature portfolio

Peer Review File

A High Dimensionality Approach Reveals Immunopathogenic Responses Driving Severe Pediatric Acute Respiratory Distress Syndrome

Corresponding Author: Dr Judith Ju Ming Wong

This file contains all reviewer reports in order by version, followed by all author rebuttals in order by version.

Version 0:

Reviewer comments:

Reviewer #1

(Remarks to the Author)
The present study investigates the immune pathogenesis of PARDS using a multidimensional approach across lung and plasma samples. It identifies three distinct dysregulated immune pathways in severe PARDS, including IFN-γ-driven suppression of IL-1β responses and impaired macrophage function, which may compromise pathogen clearance. These findings are supported by transcriptomic, proteomic, and in vitro data, highlighting potential targets for future therapeutic strategies.

A key strength of the study includes its multidimensional approach aimed at identifying immune signatures characterizing the complex disease entity of PARDS. Further an important aspect of the study is the inclusion of the lung compartment in the analysis, allowing to capture pathophysiological processes directly at the site of disease. This organ-specific approach enhances the relevance and accuracy of the identified immune signatures.

However, the study has several important limitations that require substantial revision. It is a single-center study, which limits generalizability; including additional centers would significantly strengthen its impact. Moreover, the absence of longitudinal data precludes conclusions about the temporal dynamics of the observed findings.
Patient group classification raises several concerns regarding potential confounders, such as the distribution of pathogens (viral, bacterial, fungal, or indirect) across the mild, moderate, and severe groups. This is especially relevant given known differences in immune signatures—e.g., IL-1β—in direct versus indirect ARDS.

There are also inconsistencies in sample sizes across the manuscript. For example, in Figure 2 (severe group): IL-1β (n=8), IL-1α (n=8), IL-1ra (n=6), MPO (n=10), and ELA (n=9), despite the manuscript stating 70 patients were analyzed, and Table 1 listing only 69 (mild: 18, moderate: 25, severe: 26). Pathogen identification totals only 67 patients (viral: 37, bacterial: 23, fungal: 4, none: 3), raising further questions. These inconsistencies should be addressed.

It is unclear why comparisons in e.g. Figures 1 and 2 use differing subgroup compositions. Were other comparisons not significant, or were they not performed? There is also no mention of adjustments for multiple testing.

The proposed link between IFN-γ priming and IL-1β production requires further mechanistic support. Data on cell viability, Western blots for IL-1β regulation, and inclusion of IL-18 and inflammasome-independent cytokines (e.g., TNF-α, IL-6) would help clarify the specificity of this relationship. Further this experiment raises the question of whether adult PBMCs are an appropriate model, given the authors' own emphasis in the introduction that immune pathophysiology in adults cannot be directly extrapolated to children.

My recommendation is that the manuscript, in its current form, should not be accepted for publication.

Reviewer #2

(Remarks to the Author)
Wong et al. present a high-dimensional immune profiling study of pediatric acute respiratory distress syndrome (PARDS),

using a combination of transcriptomics, mass cytometry, single-cell RNA sequencing, and in vitro modeling. The authors provide novel insights into PARDS immunopathogenesis, especially the role of cytotoxic CD8+ T cells, IFN-γ signaling, and suppressed IL-1β responses in the pulmonary myeloid compartment.The manuscript addresses a clear gap in the literature, given the relatively sparse characterization of PARDS compared to adult ARDS. The study is technically sound very good but there are several points that require clarification.

Major Comments:
In Figures 1c, 1d, and 2, the addition of a "PARDS" category alongside the stratified severity groups (mild, moderate, severe) is confusing and potentially misleading, as it represents a combination of those same data points. The authors should consider relabeling or restructuring the x-axis to avoid redundancy. Additionally, the statistical analyses in these figures are not clearly annotated. Please indicate the specific tests used and clarify whether adjustments for multiple comparisons were performed.
The manuscript inconsistently presents fold-change data (e.g., log2 in some figures, possibly log10 in others). All fold changes should be reported in a consistent format (preferably log2, in line with single-cell transcriptomics convention) and clearly stated in the figure legends and methods.
While the authors observe decreased IL-1β-related gene expression in IFN-stimulated myeloid clusters (e.g., clusters 1 and 10), the implications for PARDS pathology are underdeveloped. Specifically, pro-inflammatory clusters 3 and 7 still exhibit upregulated IL1B expression. A more nuanced discussion is needed to reconcile this heterogeneity and to explain how IL-1β expression in some clusters contributes—or fails to contribute—to the overall lung immune environment.

The manuscript would benefit from a direct comparison of PARDS immune features to publicly available adult ARDS datasets, especially at the single-cell level. For instance:
Are similar IFN-responsive macrophage clusters observed in adult ARDS? Is IL-1β suppression also a hallmark in adult viral ARDS? Addressing this would contextualize whether the identified signatures are unique to PARDS or conserved across age groups.

Figure 6 illustrates that IFN-γ suppresses IL-1β in monocytes via TLR7 signaling. However, the observation that LPS (TLR4) stimulation in IFN-γ-primed monocytes *enhances* IL-1β contradicts the central hypothesis that IL-1β suppression is a pathogenic driver of PARDS. This paradox weakens the interpretation that reduced IL-1β is inherently detrimental. To strengthen this, the authors could: Analyze whether TLR4 expression is suppressed in the myeloid single-cell data from PARDS patients. Clarify whether the observed suppression is pathway-specific (TLR7 vs. TLR4), which might reflect viral vs bacterial stimulus contexts.
The layout and labeling of panels in Figure 6 are confusing and appear arbitrarily ordered. A logical grouping of panels (e.g., dose-response first, then suppression assays, then rescue experiments) would aid readability. Additionally, statistical tests appear inconsistently applied across panels. All graphs should include clear p-values or statistical annotations, and the number of replicates should be stated.

Minor:
The terms “CD3+ IFN-γ signature” and “CD14+ myeloid dysfunction” should be explicitly defined early on, perhaps with a visual schematic, to help non-specialist readers.
Ensure all supplementary figures referenced (e.g., S1B, S3, S4) are consistently cited and their relevance to key points is clearly articulated.
The acronym "IFNg" should be replaced with “IFN-γ” throughout for clarity and formatting consistency with immunology standards.

Version 1:

Reviewer comments:

Reviewer #1

(Remarks to the Author)
Thank you for answering the questions and revising the manuscript. In my opinion, some of the biological relationships described have not been sufficiently investigated to support their current interpretation. I would suggest more cautious wording, as the data in some parts do not yet provide robust evidence of pathophysiologically relevant changes.

Given the relatively small group size, a normal distribution, which is assumed in both the Student's t-test and the Pearson correlation, seems unlikely. It is advisable to consider nonparametric alternatives such as Mann-Whitney-U-Test and Spearman’s rank correlation.

The analyses should also consistently include negative controls in order to draw valid conclusions from the data. For example Fig. 1B / Fig 2 B. In order to be able to assess any effect between the different patient groups, I believe it is essential to see whether there is any difference in gene regulation compared to the controls.

It seems not sufficient to show only the percentages of the different cell types (e.g. Fig 1 and 2) in order to answer the question of which cell types are predominant in PARDS lungs. Reporting only percentages in FACS analysis can be

misleading. Percentages, can be affected by changes in other populations, and may obscure biologically or clinically relevant differences. Absolute numbers are essential for accurate interpretation and meaningful comparisons between samples. Especially if this results in a key statement of the paper.

Although there may be a weak correlation between IFN-γ and the oxygenation index in Fig. 1, the question arises as to what biological relevance should be demonstrated here, since there is no difference in IFN-γ between controls, non-severe, and severe PARDS patients. Since IL-18 is mainly induced during viral infections and closely linked to IFN-γ signaling, including corresponding data would significantly strengthen the manuscript. As IL-18 activity depends on post-translational processing rather than transcriptional regulation, RNA-based analyses alone are insufficient to accurately assess its functional production.

Reviewer #3

(Remarks to the Author)
I thank the authors for proactively responding to the comments raised by Reviewer #2.

Version 2:

Reviewer comments:

Reviewer #1

(Remarks to the Author)
While I agree that the T cell subsets are increased in subsets in patients with PARDS as compared to age-matched controls (S1), Fig. 1c does not demonstrate a positive association with disease severity, as no differences are observed between severe and non-severe cases across any of the cell types shown. Please explain what you mean with: “This influx of T-cell subsets was positively associated with disease severity (Figure 1C).”

You state that quantitatively, the proportion of pulmonary CD14+ cells was significantly reduced in DTL samples from PARDS vs controls (Figure S1), in particular non-severe PARDS vs. controls. In Fig. 2C (CD14+ cells), this effect is observed only in non-severe PARDS; moreover, there is no significant difference between non-severe and severe cases, and no statistical difference is detected for $CD33^+$ cells. Given this, it is unclear why Fig. 1 and Fig. 2 differentiates between severe and non-severe PARDS. Please explain why certain effects are observed exclusively in non-severe PARDS, while others are evident only in severe PARDS. For me this appears highly inconsistent and may be driven by random variation rather than indicating a correlation with disease severity in either Fig. 1 or Fig. 2.

In Fig. 3, you report that alveolar myeloid cells are the most abundant cell population. This heterogeneous subset includes innate immune cells and likely comprises $CD14^+$ cells (also shown in Fig.3 C), which you previously report to be reduced. Please clarify how these findings can be reconciled.

Figure 6 is somewhat confusing. Were all cells treated with ATP as signal 2 for inflammasome activation, as indicated in the graphical abstract? This treatment is not shown in the corresponding graphs. In addition, could you provide data on cell viability? Since TLR7-mediated IL-1β production is caspase-1 dependent and therefore activates pyroptosis. The observed reduction in cytokine production, depending on the experimental setup and prior IFN-γ priming, could also be explained by increased cell death rather than reduced cytokine production. In any case, the proposed mechanism is not novel, as it was already described by Guarda et al. in Immunity (2011), and therefore adds limited new insight to the field.

After evaluation, I feel that, while the study is carefully conducted, the presented data do not provide a substantial advance for the field. Identification of subphenotypes with clear clinical relevance would ideally require data from multiple centers. Although the identified cell clusters appear plausible, their biological significance remains unclear in several instances, and any association with disease severity is only weakly supported. Additionally, the mechanistic link demonstrated in cell culture, while interesting, is in my opinion established in the literature, albeit not previously shown specifically in the context of PARDS.

Version 3:

Reviewer comments:

Reviewer #1

(Remarks to the Author)

Open Access This Peer Review File is licensed under a Creative Commons Attribution 4.0 International License, which permits use, sharing, adaptation, distribution and reproduction in any medium or format, as long as you give appropriate credit to the original author(s) and the source, provide a link to the Creative Commons license, and indicate if changes were made.
In cases where reviewers are anonymous, credit should be given to 'Anonymous Referee' and the source.
The images or other third party material in this Peer Review File are included in the article's Creative Commons license, unless indicated otherwise in a credit line to the material. If material is not included in the article's Creative Commons license and your intended use is not permitted by statutory regulation or exceeds the permitted use, you will need to obtain permission directly from the copyright holder.
To view a copy of this license, visit https://creativecommons.org/licenses/by/4.0/

<u>Title: A High Dimensionality Approach Reveals Immunopathogenic Responses Driving Severe Pediatric Acute Respiratory Distress Syndrome</u>

<u>Manuscript no.: NCOMMS-25-32018-T</u>

Reviewer #1 (Remarks to the Author):

The present study investigates the immune pathogenesis of PARDS using a multidimensional approach across lung and plasma samples. It identifies three distinct dysregulated immune pathways in severe PARDS, including IFN-γ-driven suppression of IL-1β responses and impaired macrophage function, which may compromise pathogen clearance. These findings are supported by transcriptomic, proteomic, and in vitro data, highlighting potential targets for future therapeutic strategies.

A key strength of the study includes its multidimensional approach aimed at identifying immune signatures characterizing the complex disease entity of PARDS. Further an important aspect of the study is the inclusion of the lung compartment in the analysis, allowing to capture pathophysiological processes directly at the site of disease. This organ-specific approach enhances the relevance and accuracy of the identified immune signatures.

However, the study has several important limitations that require substantial revision. It is a single-center study, which limits generalizability; including additional centers would significantly strengthen its impact.

We are in full agreement with the reviewer that an independent validation of our findings is desirable. To this end, we have employed two independent cohorts and shown the reproducibility of the data. We agree that including patient samples from a second site would strengthen the generalizability of our findings, however, we do not have the opportunity to recruit from an independent site. Instead, we have performed the validation in a new independent cohort of patients recruited from our centre which confirm the association between PARDS severity (oxygenation index) and pulmonary IL-1β concentration. The recruitment period of the original and validation cohort are Oct 2018 to August 2023 and September 2023 to December 2024, respectively.

The purpose of making the data available with this publication is to also provide the opportunity to disseminate the knowledge and entice other investigators to repeat the study and ensure its universal validity. We are at full disposal to facilitate this process, which is beyond the first reasonable objective for a first observation.

In the revision, we have added the following sentence and analysis:

"An independent cohort under the same study protocol was recruited from the same site over September 2023 to December 2024 as a validation cohort."

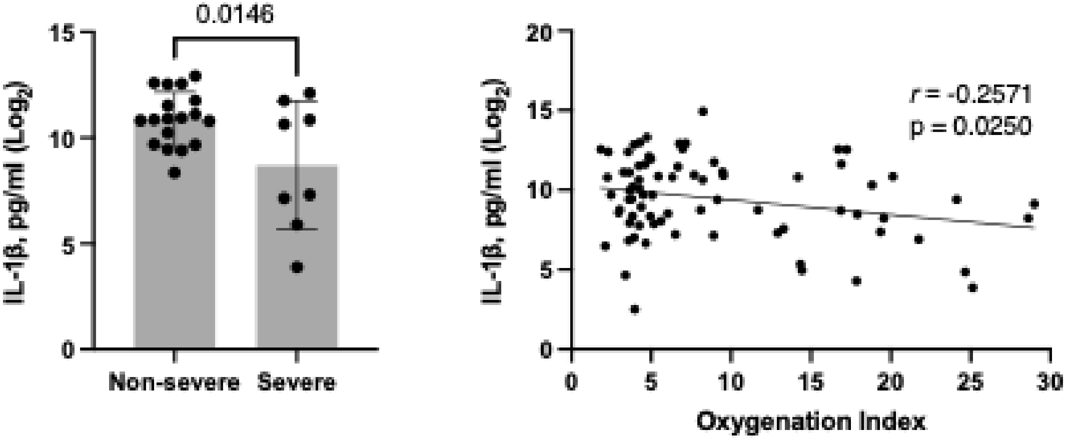

Figure S3A: IL-1β concentrations in deep tracheal lavage samples from the independent cohort of PARDS patients

A.IL-1β concentrations in severe vs. non-severe PARDS and its correlation with the oxygenation index. Samples from 26 patients with PARDS were obtained at diagnosis and daily for the first 7 days of PARDS. Student's t-test comparison of IL-1β concentration between non-severe vs. severe PARDS. Pearson correlation, *r*, of IL-1β concentration and the oxygenation index.

Moreover, the absence of longitudinal data precludes conclusions about the temporal dynamics of the observed findings.

We are in full agreement with the reviewer and in the revision present available longitudinal data from the original cohort with samples taken from patients who progress to severe PARDS below along with the oxygenation index calculated at the time of sampling showing the inverse relationship between IL-1β and OI i.e. with increasing OI, there is a reduction in IL-1β. In contrast, IFN-γ does not follow this trajectory, being highest at the start of illness and trending downward during the course of illness consistent with its priming effect.

Figure S2

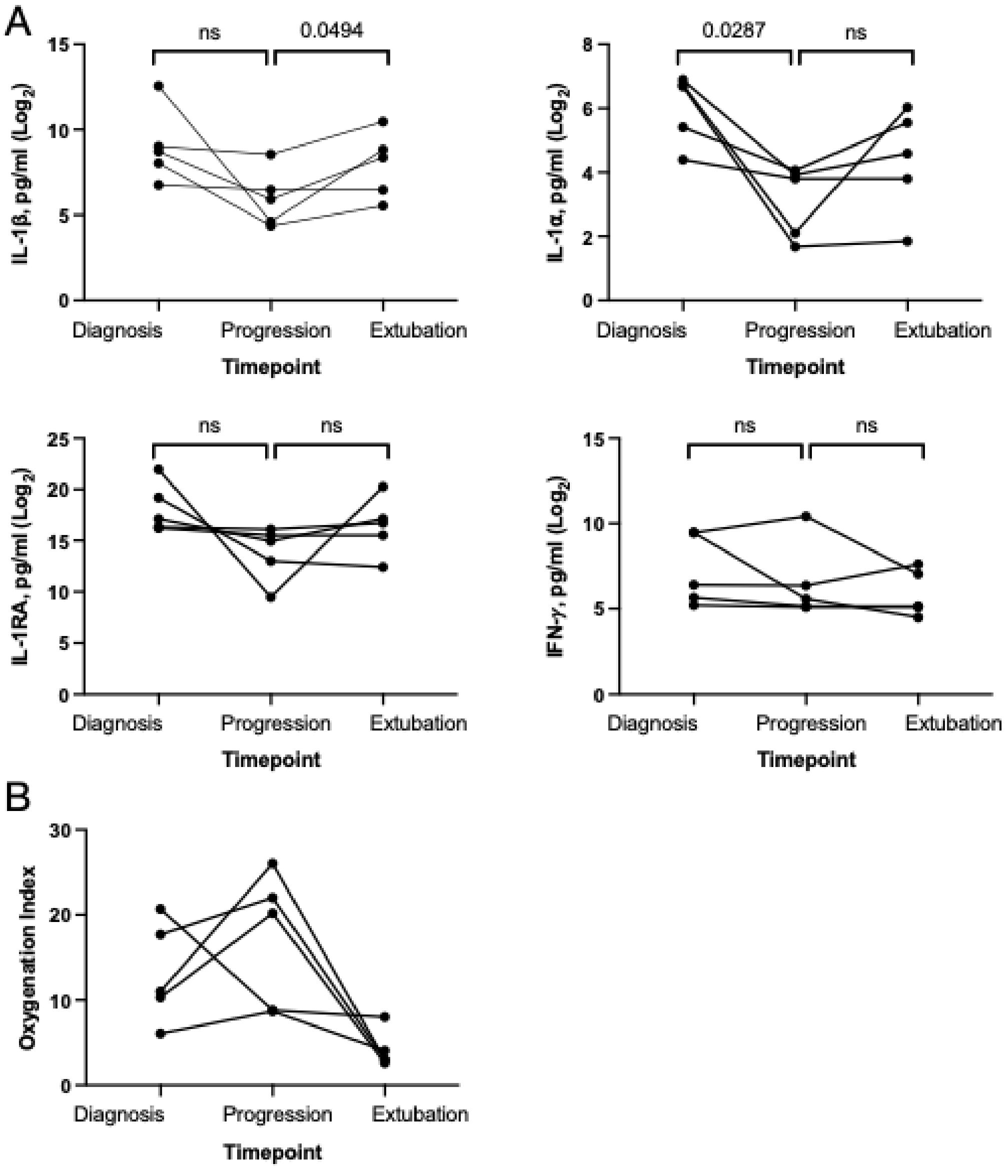

Figure S2: Cytokine trend in deep tracheal lavage samples from PARDS patients (original cohort) who progressed in severity after diagnosis

Samples from 5 patients with PARDS were obtained at 3 timepoints. A. IL-1β, IL-1α and IL-1RA levels at diagnosis, progression of disease and resolution of disease (extubation). B.Oxygenation index calculated at the point of sampling. Student's t-test was used for two group comparisons of cytokine concentrations between diagnosis vs. progression and progression vs. extubation samples.

Longitudinal data taken from the independent validation cohort shows a consistent trend and is presented below.

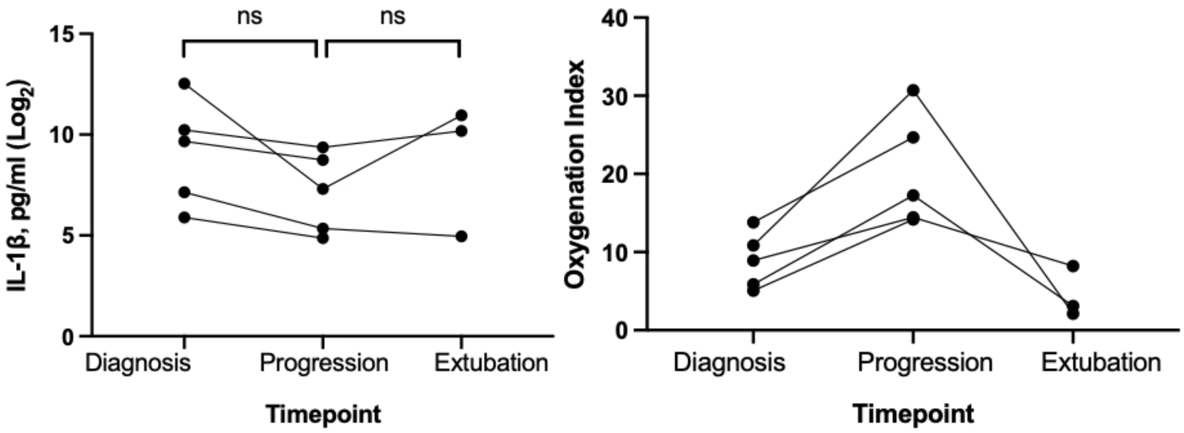

Figure S3: IL-1β concentrations in deep tracheal lavage samples from the independent cohort of PARDS patients

B.IL-1β at diagnosis vs. progression and progression vs. extubation samples with corresponding oxygenation index. Samples from 5 patients with PARDS were obtained at 3 timepoints. Two patients in this cohort died and do not have extubation samples. Student's t-test was used for two group comparisons.

Patient group classification raises several concerns regarding potential confounders, such as the distribution of pathogens (viral, bacterial, fungal, or indirect) across the mild, moderate, and severe groups. This is especially relevant given known differences in immune signatures—e.g., IL-1β—in direct versus indirect ARDS.

We considered this concern when we designed the study and anticipated that direct PARDS may be mechanistically different from indirect PARDS [1]. Moreover, the natural epidemiology of triggers for PARDS are mostly direct triggers (approximately 80% in epidemiological studies) [2, 3]. Hence, our study is mainly relevant for direct PARDS. We have amended the manuscript to ensure this is clearly stated and propose a new study to compare direct vs. indirect or sepsis, ARDS vs. sepsis+ARDS groups.

In the revision, we have added the following sentences in the Results and Discussion section to indicate that the majority of patients in our study consisted of direct viral PARDS.

"The majority of cases had direct PARDS 61/69 (88.4%) and of these, a viral pathogen was identified in 37/69 (53%)."

"TLR7 recognises viral ssRNA in keeping with the common triggering agent in PARDS."

"As the majority of cases included in this study had direct PARDS triggers, future work should examine differences, if any, between indirect and direct PARDS."

In the manuscript, viral PARDS was defined as PARDS caused by pneumonia whereby a viral or no pathogen (presumed viral) was identified, this included mixed infections where a virus was identified. Bacterial PARDS was defined as PARDS caused by pneumonia whereby a sole bacterial pathogen was identified. We have included this in the methods section:

"In this study, viral PARDS was defined as PARDS caused by pneumonia whereby a viral or no pathogen (presumed viral) was identified, this included mixed infections where a virus was identified."

We present to the reviewers, analyses comparing the cytokine profile of bacterial vs. viral PARDS and in particular, pulmonary IL-1β concentration in mild, moderate and severe bacterial PARDS. IL-1β, IL-1α and MPO were higher in bacterial PARDS compared to viral PARDS and there was no trend of reducing IL-1β across severity groups as was observed in viral PARDS. As the number of patients with bacterial ARDS were too small to make meaningful comparisons we elected not to include this in the manuscript.

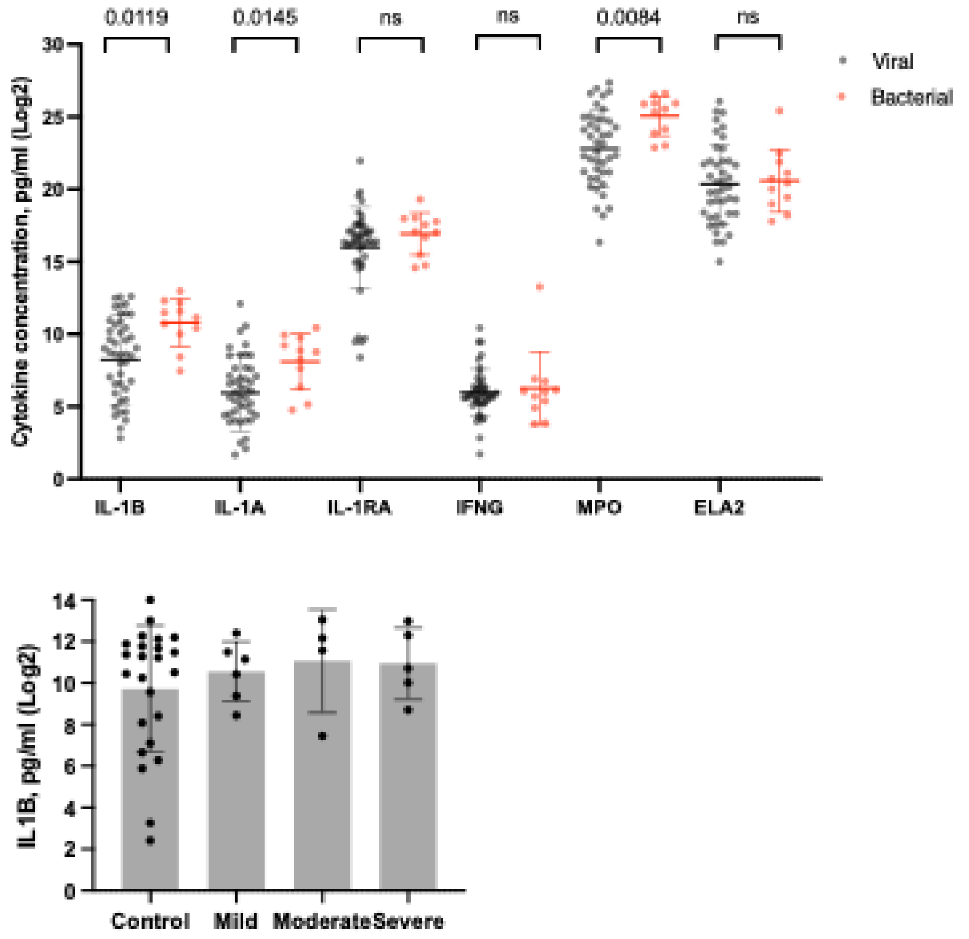

Figure R1.1: Comparison of cytokine concentrations at PARDS diagnosis between viral and bacterial PARDS (top) and cytokine concentrations across bacterial PARDS severity groups.

There are also inconsistencies in sample sizes across the manuscript. For example, in Figure 2 (severe group): IL-1β (n=8), IL-1α (n=8), IL-1ra (n=6), MPO (n=10), and ELA (n=9), despite the manuscript stating 70 patients were analyzed, and Table 1 listing only 69 (mild: 18, moderate: 25, severe: 26). Pathogen identification totals only 67 patients (viral: 37, bacterial: 23, fungal: 4, none: 3), raising further questions. These inconsistencies should be addressed.

We thank the reviewer for identifying this error – some of the dots had been cut-off due to the range of the Y-axes. We have rectified this and all dots are now represented in each figure. We have also added information regarding the number of samples used in the footnote of each figure. We have also amended the number of pathogens isolated from patients in Table 1.

Table 1: Clinical characteristics of patients with PARDS

Clinical characteristics	Total, n=69
**Pathogen isolated**	
**Virus**	**37 (53.6)**
**Bacteria**	**25 (36.2)**
**Fungal**	**5 (7.3)**
**None**	**13 (18.8)**

Some patients had mixed infections and the total exceeds 69 patients.

It is unclear why comparisons in e.g. Figures 1 and 2 use differing subgroup compositions. Were other comparisons not significant, or were they not performed? There is also no mention of adjustments for multiple testing.

We have amended the figure legend for Figures 1 and 2 to include information on all subgroup comparisons performed and correction for multiple testing. To minimise multiple comparisons, we have also reduced the number of subgroups from mild, moderate, vs. severe to non-severe vs. severe.

The proposed link between IFN-γ priming and IL-1β production requires further mechanistic support. Data on cell viability, Western blots for IL-1β regulation, and inclusion of IL-18 and inflammasome-independent cytokines (e.g., TNF-α, IL-6) would help clarify the specificity of this relationship. Further this experiment raises the question of whether adult PBMCs are an appropriate model, given the authors' own emphasis in the introduction that immune pathophysiology in adults cannot be directly extrapolated to children.

We thank the reviewer for these constructive comments and provide further data to support our findings. Regarding cell viability, the original flow cytometry analysis had already gated out dead cells and show that IL-1β expression was increased in *live* monocytes upon R837 stimulation (new Figure 7) – expression of IL-1β in *live* monocytes were then shown to be decreased when IFN-γ priming was used. We have included a representative plot of data from flow cytometry of live $CD14^{+}$ monocyte singlet cells.

Regarding mechanistic support with western blot, we have added data which showed that IL-1β was produced upon TLR7 activation by R837 and this was suppressed by IFN-γ priming. Relevant proteins in the classical inflammasome pathway were also examined. However, there was no observable change in band intensity for full-length caspase 1, it's cleaved product p20, full-length GSDMD or it's cleaved product, GSDMD-N. This may indicate that activation of the classical inflammasome pathway was not involved in R837 dependent IL-1β production.

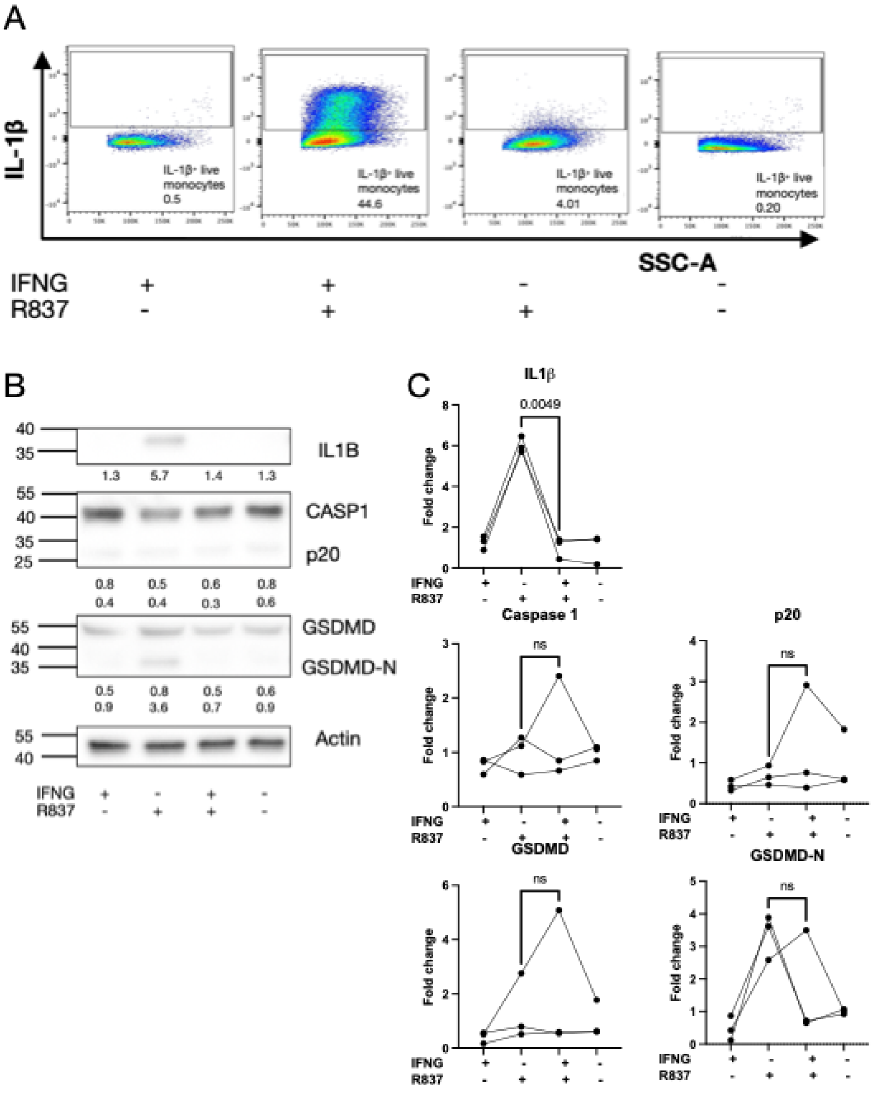

Figure S9: IL-1β production in an *in vitro* cell culture model of CD14⁺ monocytes

A. Representative flow cytometry bivariate plot demonstrating IL-1β production in live CD14⁺ monocytes with different *in vitro* monocyte culture conditions B. Detection of IL-1β and inflammasome proteins by Western blot under various culture conditions. Band intensity was measured using ImageJ version 1.53m

Regarding IL-18 and inflammasome-independent cytokines (e.g., TNF-α, IL-6), we refer to the bulk RNA sequencing data from the *in vitro* monocyte culture. *IL6* mRNA expression was increased upon TLR7 activation by R837 (Figure 8) and IFN-γ priming

suppressed transcription of *IL6*, along with *IL1B*. However, *TNFA* gene did not show the same trend (we have added this in Figure 8). Though IL-6 and TNF-α are products of NFκB and MAPK pathways in common with IL-1β, previous studies have reported the cytokine profile upon R837 stimulation of human monocytes which induces IL-1β and IL-6 but not TNF-α – this is consistent with our data [4]. In contrast, R848 (TLR7/8 agonist) stimulation of human monocytes induces all three cytokines. IL-18 was also not upregulated by R837 (we have added this in Figure 8), supporting the postulation that IL-1β production downstream of TLR7 activation pathway may not be dependent on the classical inflammasome pathway.

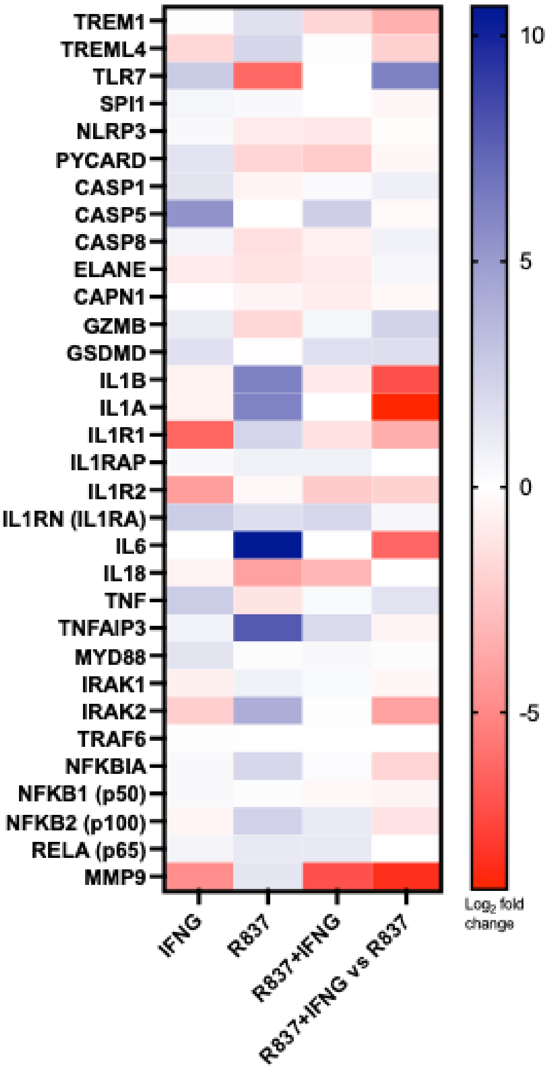

Figure 7: Bulk RNA sequencing of *in vitro* monocyte conditions mapped alongside NanoString and single-cell RNA sequencing clinical samples

Lastly, we believe that adult PBMC/monocytes are representative of the immune pathophysiology occurring in PARDS, though we were unable to obtain sufficient number of PBMC/monocytes from children to show it. Children have been shown to express a

high basal interferon tone as compared to adults in both the healthy state and during acute viral infection (COVID-19) [5]. This is postulated to be due to recurrent viral infections in childhood. We postulate that the higher interferon tone behaves as the priming effect seen in our *in vitro* monocyte culture from adult donors. A study to directly compare pediatric and adult IFN-γ and IL-1β responses during viral ARDS would be necessary in the future to confirm this hypothesis.

We attempted to expose healthy pediatric and PARDS disease PBMCs to the *in vitro* cell culture conditions, but experienced several challenges. Foremost, the number of viable pediatric PBMCs obtained after cryopreservation were limited (0.2-2.0 x10^6) precluding the possibility of performing further monocyte purification. R837-dependent IL-1β production was suppressed by IFN-γ in PBMCs from healthy children, but not PARDS patients. This may potentially be because PBMCs are already primed *in vivo* during the acute infection in PARDS and no longer demonstrate further suppression of IL-1β with *in vitro* IFN-γ priming.

We have added the following sentences to the Results and Discussion section:

"This suppression was evident in PBMCs from healthy children, but not PARDS patients (Figure S8)."

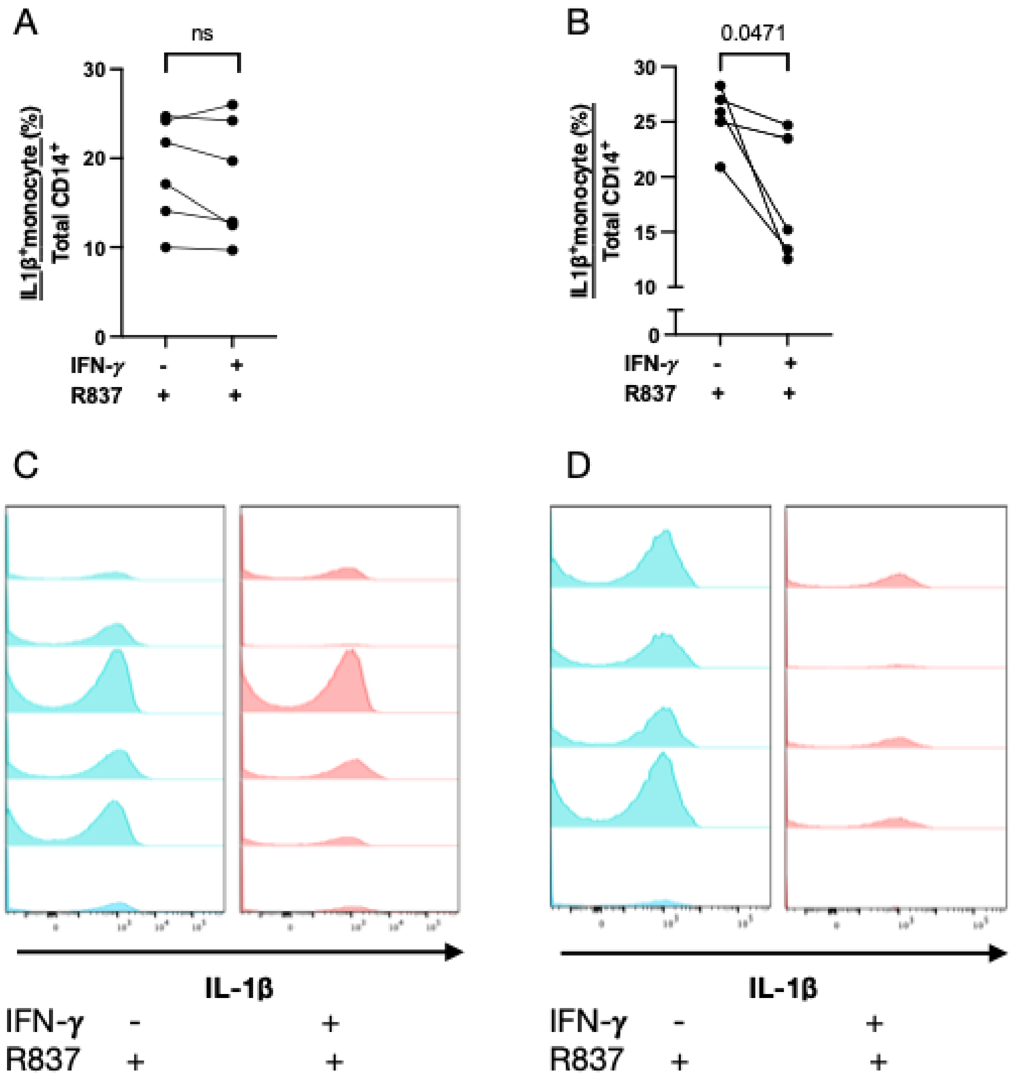

Figure S8: Suppression of R837-dependent IL-1β production upon priming with IFN-γ in healthy children and PARDS PBMCs

PBMCs from 5 healthy children and 6 patients with PARDS were exposed to TLR7 activation with 50μg/ml of R837 with and without 100ng/ml of IFN-γ priming. IL-1β expression was determined by flow cytometry using the BD FACSDiscover™ S8. A,Percentage IL-1β^{+} monocytes of total CD14^{+} in PARDS and B, control. C, Median fluoresnce intensity (MFI) of IL-1β expression in CD14^{+} monocytes in PARDS and D, control. E, MFI plot of IL-1β expression in CD14^{+} monocytes in PARDS and F, control.

"Although the *in vitro* monocyte model utilized healthy adult PBMCs or monocytes, we believe that it can to recapitulate the immune pathophysiology of PARDS. Children have been shown to express a high basal interferon tone as compared to adults in both the healthy state and during acute viral infection (COVID-19) [5]. This was postulated to be due to frequent and recurrent asymptomatic viral infections in childhood. We postulate that the higher interferon tone behaves as the priming effect seen in our *in vitro* monocyte

model from adult donors. Indeed, PBMCs from healthy children exhibited a consistent suppression of IL-1β production with IFN-γ priming. However, children with PARDS who had been recently exposed to a respiratory viral infection (and presumably developed an interferon response *in vivo*) showed no further suppression when primed *in vitro* with IFN-γ. Patients who develop severe PARDS possibly experience an intense IFN-γ response, are hyperresponsive to the IL-1 suppressive effects of IFN-γ or already have a high basal expression of interferon resulting in low IL-1β expression. A study to directly compare pediatric and adult IFN-γ and IL-1β responses during viral ARDS would be necessary in the future to confirm this hypothesis."

My recommendation is that the manuscript, in its current form, should not be accepted for publication.

Reviewer #2 (Remarks to the Author):

Wong et al. present a high-dimensional immune profiling study of pediatric acute respiratory distress syndrome (PARDS), using a combination of transcriptomics, mass cytometry, single-cell RNA sequencing, and in vitro modeling. The authors provide novel insights into PARDS immunopathogenesis, especially the role of cytotoxic CD8+ T cells, IFN-γ signaling, and suppressed IL-1β responses in the pulmonary myeloid compartment.The manuscript addresses a clear gap in the literature, given the relatively sparse characterization of PARDS compared to adult ARDS. The study is technically sound very good but there are several points that require clarification.

Major Comments:

In Figures 1c, 1d, and 2, the addition of a "PARDS" category alongside the stratified severity groups (mild, moderate, severe) is confusing and potentially misleading, as it represents a combination of those same data points. The authors should consider relabeling or restructuring the x-axis to avoid redundancy. Additionally, the statistical analyses in these figures are not clearly annotated. Please indicate the specific tests used and clarify whether adjustments for multiple comparisons were performed.

We thank the reviewer for this feedback. In the revision we have removed the "PARDS" category from main Figure 1C, 1D, 2C, 2D, and 2E and transferred these data to the supplement (Figure S1). We have also amended the figures and figure legends to include information on all subgroup comparisons performed and any correction for multiple testing. To minimise multiple comparisons, we have also reduced the number of subgroups from mild, moderate, vs. severe to non-severe vs. severe.

The manuscript inconsistently presents fold-change data (e.g., log2 in some figures, possibly

log10 in others). All fold changes should be reported in a consistent format (preferably log2, in line with single-cell transcriptomics convention) and clearly stated in the figure legends and methods.

We confirm that Log_2 was used in all single-cell RNA sequencing analyses and have included this in the figure legends.

While the authors observe decreased IL-1β-related gene expression in IFN-stimulated myeloid clusters (e.g., clusters 1 and 10), the implications for PARDS pathology are underdeveloped. Specifically, pro-inflammatory clusters 3 and 7 still exhibit upregulated IL1B expression. A more nuanced discussion is needed to reconcile this heterogeneity and to explain how IL-1β expression in some clusters contributes—or fails to contribute—to the overall lung immune environment.

We thank the reviewer for this thoughtful comment. In the revision, we have added a more in-depth discussion on the pro-inflammatory clusters 3 and 7 in the Discussion section:

"While we observed a decrease in IL-1β-related gene expression in IFN-stimulated myeloid clusters (1 and 10), some PARDS clusters (3 and 7) still exhibited an upregulated *IL1B* expression, indicating that at least at the sampling timepoint, heterogenous cell populations co-exist in the lung microenvironment. This is consistent with single-cell RNA sequencing data from children with PARDS and adults with critical COVID-19 which also exhibits heterogenous monocyte and macrophage populations of differing pro-inflammatory and IFN states [6, 7]. Further work is needed to elucidate the role of each of these cell populations in driving severe disease. However, despite the presence of heterogeneous cell clusters, our study demonstrated an overall reduction in IL-1β protein in severe disease."

The manuscript would benefit from a direct comparison of PARDS immune features to publicly available adult ARDS datasets, especially at the single-cell level. For instance: Are similar IFN-responsive macrophage clusters observed in adult ARDS? Is IL-1β suppression also a hallmark in adult viral ARDS? Addressing this would contextualize whether the identified signatures are unique to PARDS or conserved across age groups.

We are in full agreement with the reviewer that a comparison with published adult datasets would be useful. We performed a literature search to identify single-cell RNA sequencing data of lung fluid/bronchoalveolar lavage from patients with ARDS (table below). We found that most ARDS studies performed single-cell RNA sequencing on whole blood/PBMC samples and that lung fluid/bronchoalveolar lavage data was only available for severe/critical COVID-19 infection. Studies utilizing bulk RNA/ microarray trancriptomics were considered unsuitable for comparison with our single-cell dataset.

Table R1.1: Summary of single-cell RNA sequencing studies in ARDS

Study	Cohort	Sample origin	Methods	Accession no.
Ferlage 2023*	Pediatric ARDS, n=12	Tracheal aspirate	scRNA-sequencing	PRJNA971535
Grant 2021*	COVID-19 n=10	BAL	scRNA-sequencing	GSE155249
Liao 2020	COVID-19 Severe/ critical, n=6	BAL	scRNA-sequencing	GSE145926
Wauters 2021	COVID-19 Critical, n=26	BAL	scRNA-sequencing	EGAS00001004717
He 2020	COVID-19 Severe, n=3	BAL	scRNA-sequencing	GSE147143
Chua 2020	COVID-19 n=24	BAL	scRNA-sequencing	EGAS00001004481
Song 2023	Pediatric ARDS, n=1	Lung tissue Blood	scRNA-sequencing	GSE223793
Melms 2021	COVID-19	Lung tissue	scRNA-sequencing	GSE171524
Boyd 2021	Influenza A infection	Lung tissue	scRNA-sequencing	PRJNA613670
Habermann 2020	Pulmonary fibrosis	Lung tissue	scRNA-sequencing	GSE135893
Tsukui 2020	Pulmonary fibrosis	Lung tissue	scRNA-sequencing	GSE132771
Reyfman 2019	Pulmonary fibrosis	Lung tissue	scRNA-sequencing	GSE122960
Valenzi 2019	Systemic sclerosis-interstitial lung disease	Lung tissue	scRNA-sequencing	GSE128169
Vieira Braga 2019	Asthma	Lung tissue	scRNA-sequencing	GSE130148
Qian 2023	Sepsis ARDS	PBMC	scRNA-sequencing	GSE242127
Sinha 2022	COVID-19 Bacterial ARDS/ sepsis	Peripheral blood leukocytes and lymphocytes	scRNA-sequencing	GSE157789
	Moderate-severe COVID-19	BAL	scRNA-sequencing	GSE145926
	Bacterial ARDS/ sepsis	BAL	scRNA-sequencing	GSE167118
Li 2022	COVID-19 ARDS	Whole blood	scRNA-sequencing	GSE172114
Yao 2021	ARDS	PBMC	scRNA-sequencing	GSE154567
Ray 2020	ARDS	PBMC	scRNA-sequencing	GSE151263
Jiang 2020	ARDS	PBMC	scRNA-sequencing	GSE151263
Park 2022	COVID-19	Autopsy tissue	Shotgun meta-transcriptomics	GSE159678
Sarma 2021	COVID-19 ARDS	Tracheal aspirate	Bulk RNA sequencing	GSE163426
Overmyer 2021	COVID-19	Leukocytes	Bulk RNA sequencing	GSE157103

Borrmann 2023	COVID-19 ARDS	Whole blood	Bulk RNA sequencing	
Eltobgy 2024	ARDS<br>COVID-19 ARDS	PBMC	Bulk RNA sequencing	GSE273149
Dolinay 2012	ALI	Whole blood	Microarray	GSE32707
Howrylak 2009	ALI	Whole blood	Microarray	GSE10474
Gharib 2018	ARDS	PBMC<br>Alveolar macrophage	Microarray	GSE89953
Kangelaris 2015	ARDS	Whole blood	Microarray	GSE66890
Bradley 2016	ARDS	BAL<br>Blood	Microarray	GSE76293
Liao 2021	ARDS	PBMC	Microarray	GEO176529
Bos 2018	ARDS	Whole blood	Microarray	GSE65682
Warner 2014	ARDS	Blood<br>BAL	Microarray	NA
Morrell 2018	ARDS<br>n=26	Alveolar macrophages<br>PBMC	Microarray	GSE89953
Kangelaris 2015	Sepsis ARDS	Whole blood	Microarray	GSE66890
Morrell 2019	ARDS<br>n=35	Alveolar macrophages	Microarray	GSE116560
Kovach 2015	ARDS	Alveolar macrophages<br>Peripheral leukocytes	Microarray	NA
Nick 2016	Sepsis ARDS	Peripheral neutrophils	Microarray	GSE3037
Najafipour 2022	COVID-19 ARDS	BAL<br>Blood	miRNA sequencing	NA

*Used as comparison datasets

Hence, we selected one pediatric ARDS and one adult critical COVID-19 single-cell RNA sequencing dataset of lung fluid/bronchoalveolar lavage for analysis. We found that a similar IFN-gene response high–*IL1B* gene low phenomena in *CD14* expressing myeloid cells (Figure S11) in the pediatric ARDS dataset. In the adult dataset, however, there were 2 clusters co-expressing IFN-response genes and *IL1B* genes and 1 cluster which expressed IFN response genes (*CXCL10*) without *IL1B* genes (Figure S12). Both studies did not show corresponding IL-1β protein concentrations.

In the revision, we have included the comparison and a discussion of the IFN and IL1 signature from our study with the adult (GSE155249) and pediatric dataset (PRJNA971535):

"To contextualize whether the identified signatures were unique to PARDS or conserved across age groups, we compared our data with two available published single-cell RNA sequencing datasets. The first, a pediatric single-cell RNA sequencing dataset of tracheal

aspirates from young children with PARDS, we observe a similar IFN-gene response high–*IL1B* gene low phenomena in *CD14* expressing myeloid cells (Figure S11). In this comparison dataset, 12 patients with PARDS were included – 11/12 (92%) had a viral pathogen detected, of which 7/12 (58%) had RSV. Using the monocyte *in vitro* cell culture bulk RNA sequencing gene module, upregulated genes correspond to IFN-stimulated clusters and downregulated genes correspond to *IL1B* expressing clusters. The second, an adult critical COVID-19 bronchoalveolar lavage dataset, an enriched population of CD4$^+$, CD8$^+$ T cells and monocytes exhibiting a strong *IFNG* response was observed. Here, though there were cell clusters co-expressing IFN-response genes and *IL1B* genes (TRAM2 and MoAM1), there were also clusters which expressed IFN response genes (*CXCL10*) without *IL1B* genes (MoAM2). Applying the monocyte *in vitro* cell culture bulk RNA sequencing gene module showed that cells expressing *ISG15* and *STAT1* expressed little *IL1B* (Figure S12).”

Figure 6 illustrates that IFN-γ suppresses IL-1β in monocytes via TLR7 signaling. However, the observation that LPS (TLR4) stimulation in IFN-γ-primed monocytes *enhances* IL-1β contradicts the central hypothesis that IL-1β suppression is a pathogenic driver of PARDS. This paradox weakens the interpretation that reduced IL-1β is inherently detrimental. To strengthen this, the authors could: Analyze whether TLR4 expression is suppressed in the myeloid single-cell data from PARDS patients. Clarify whether the observed suppression is pathway-specific (TLR7 vs. TLR4), which might reflect viral vs bacterial stimulus contexts.

We thank the reviewer for this thoughtful comment. Based on our patient data most cases of PARDS were triggered by viral agents and therefore, TLR7 was thought to mediate the immune pathophysiologic responses. We showed that it is through the TLR7 pathway that IFN-γ suppresses IL-1β production in primary human monocytes consistent with a viral cause of PARDS. In the revision, we have added sentences in the Results and Discussion section to indicate that the majority of patients in our study consisted of viral PARDS.

“The majority of cases had direct PARDS 61/69 (88.4%) and of these, a viral pathogen was identified in 37/69 (53%).”

“TLR7 recognises viral ssRNA in keeping with the common triggering agent in PARDS.”

We show (above) that bacterial PARDS is not associated with a similar IL-1β suppression and this is consistent with published data demonstrating that through the TLR4 pathway IFN-γ enhances IL-1β production. The contrasting effect of IFN-γ priming on TLR4 activation may be species specific and articles shortlisted here based on human myeloid cells, report a consistent synergistic effect of IFN-γ priming on TLR4 activation [8-10].

- **Qiao Y, Giannopoulou Eugenia G, Chan Chun H et al. Synergistic Activation of Inflammatory Cytokine Genes by Interferon-γ-Induced Chromatin Remodeling and Toll-like Receptor Signaling. Immunity 2013; 39(3):454-469. https://doi.org/10.1016/j.immuni.2013.08.009**

- Masters S, Mielke L, Cornish A et al. Regulation of interleukin-1β by interferon-γ is species specific, limited by suppressor of cytokine signalling 1 and influences interleukin-17 production. EMBO reports 2010; 11:640-646. 10.1038/embor.2010.93
- Chen J, Ivashkiv LB. IFN-γ abrogates endotoxin tolerance by facilitating Toll-like receptor-induced chromatin remodeling. Proceedings of the National Academy of Sciences 2010; 107(45):19438-19443. doi:10.1073/pnas.1007816107

Regarding TLR expression in PARDS, data from our *NanoString* analysis show that TLR7 gene expression was upregulated in severe PARDS vs. control, whereas TLR4 was down regulated in moderate and severe PARDS vs control.

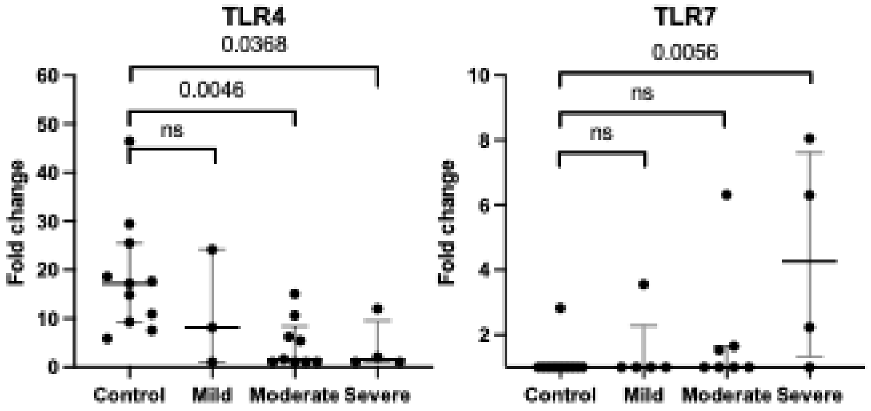

Figure R1.2: TLR4 and TLR7 expression in control, mild, moderate and severe PARDS. Data is derived from *NanoString*. Student's t-test was used for two group comparisons of gene fold change between mild vs. control, moderate vs. control and severe vs. control.

Regarding the mechanism, TLR4 and TLR7 pathways are presented in the figure below and show that both TLRs activate cytokine expression by the MAPK and NFKB pathways. Since these pathways are common between the TLRs, it is likely that the mechanism of differential IL-1β regulation seen in the *in vitro* cell culture occurs proximal to these pathways. In this regard, we observe differential TREM1 and TREML4 gene expression elicited upon TLR4 and TLR7 activation, with LPS causing an upregulation and R837 causing a downregulation of these genes. Activation of TREM1 together with TLR4 has been shown to amplify production of proinflammatory cytokines [11, 12]. Previous studies have shown that TLR4-dependent TRIF-IFN pathway activation (unique to TLR4, absent in TLR7) is required for TREM1 upregulation[13]. While the effect of TREM1 in TLR7 signalling is unclear, TREML4 has been reported to amplify cytokine production as a result of TLR7 activation [14]. Hence, the effect of IFN-γ priming on downregulating these adaptor proteins may play a role in the respective TLR4 and TLR7 responses.

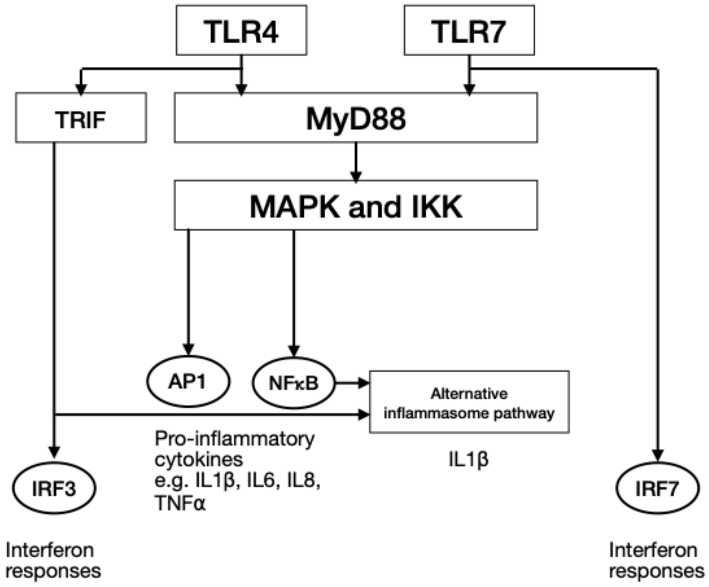

Figure R1.3: Summary of the TLR4 and TLR7 pathways

The layout and labelling of panels in Figure 6 are confusing and appear arbitrarily ordered. A logical grouping of panels (e.g., dose-response first, then suppression assays, then rescue experiments) would aid readability. Additionally, statistical tests appear inconsistently applied across panels. All graphs should include clear p-values or statistical annotations, and the number of replicates should be stated.

We thank the reviewer for these constructive comments. We have amended the layout of Figure 6 as suggested. For clarity, figures have been split into Figure 6 and 7. All statistical tests are annotated in the figure with adjusted p-values. The number of replicates are included in the legend.

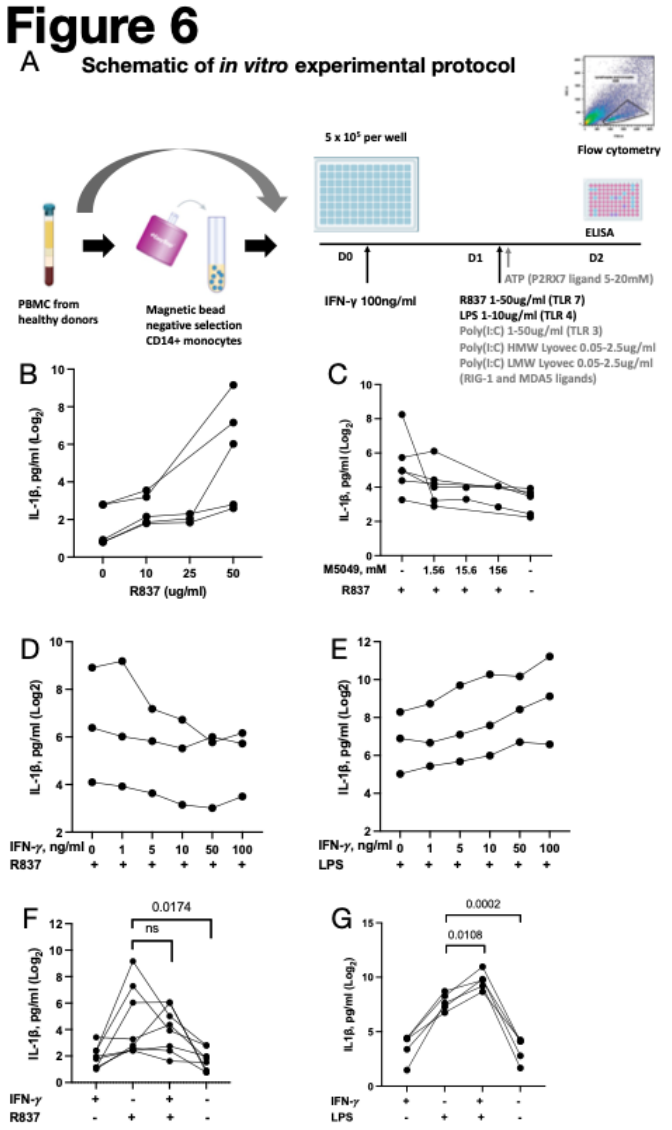

Figure 6: *In vitro* human PBMC model suggesting the mechanism of IL1 suppression in severe PARDS

A. Schematic of the *in vitro* experimental protocol. B. Dose-response plot of IL-1β production across increasing concentrations of R837. C. IL-1β suppression by increasing doses of M5049 (specific TLR7 antagonist) 3hours prior to R837 activation. D, E. IL-1β production after 24 h priming with increasing concentration of IFN- γ followed by R837 and LPS stimulation. F. Summary plot of IL-1β production upon activation with 50ug/ml R837 or G. 10ug/ml LPS with and without IFN- γ priming. D. Suppression of R837 (at 50μg/ml) dependent IL-1β production after 24 h priming with increasing concentration of IFN- γ . E. Enhancement of LPS (at 10μg/ml) dependent IL-1β production after 24 h priming with increasing concentration of IFN- γ . F. Summary plot of IL-1β production upon activation with 50μg/ml R837 and G. 10μg/ml LPS with and without 100ng/ml IFN-γ priming. Student's t-test was used for two group comparisons. Only significant *p* values

after Bonferroni correction were reported. Experimental conditions for each donor sample were performed in duplicates.

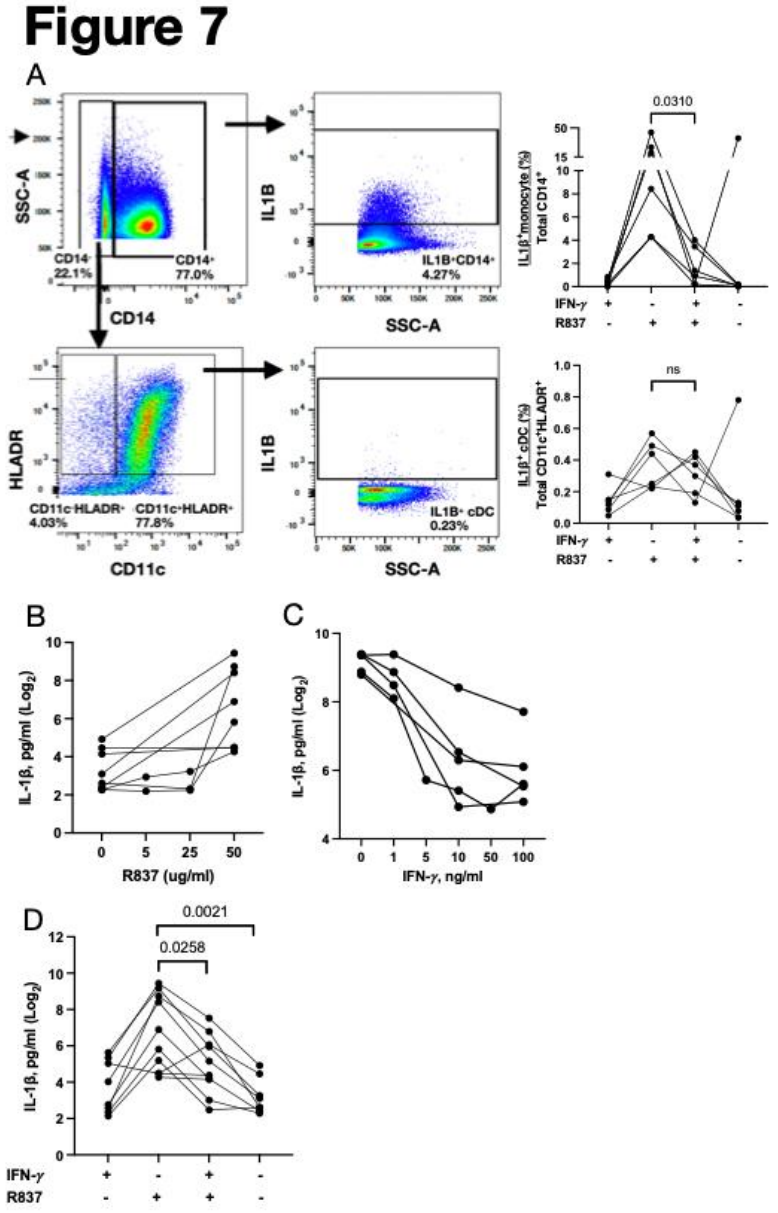

Figure 7: *In vitro* human monocyte model recapitulating the mechanism of IL1 suppression in severe PARDS

A. Flow cytometry gating strategy and percentage of IL-1β producing monocytes and dendritic cells. B. Dose-response plot of IL-1β production in monocytes across increasing R837 concentration. C. Suppression of R837 (at 50μg/ml) dependent IL-1β production in monocytes by increasing doses of IFN-γ priming. D. Summary plot of IL-1β production in monocytes upon activation with 50μg/ml R837 with and without 100ng/ml IFN-γ priming. Student's t-test was used for two-group comparisons. Only significant *p* values

after Bonferroni correction were reported. Experimental conditions for each donor sample were performed in duplicates.

Minor:
The terms “CD3+ IFN-γ signature” and “CD14+ myeloid dysfunction” should be explicitly defined early on, perhaps with a visual schematic, to help non-specialist readers.

Thank you for this comment. We have added a visual schematic/ graphical abstract of the study.

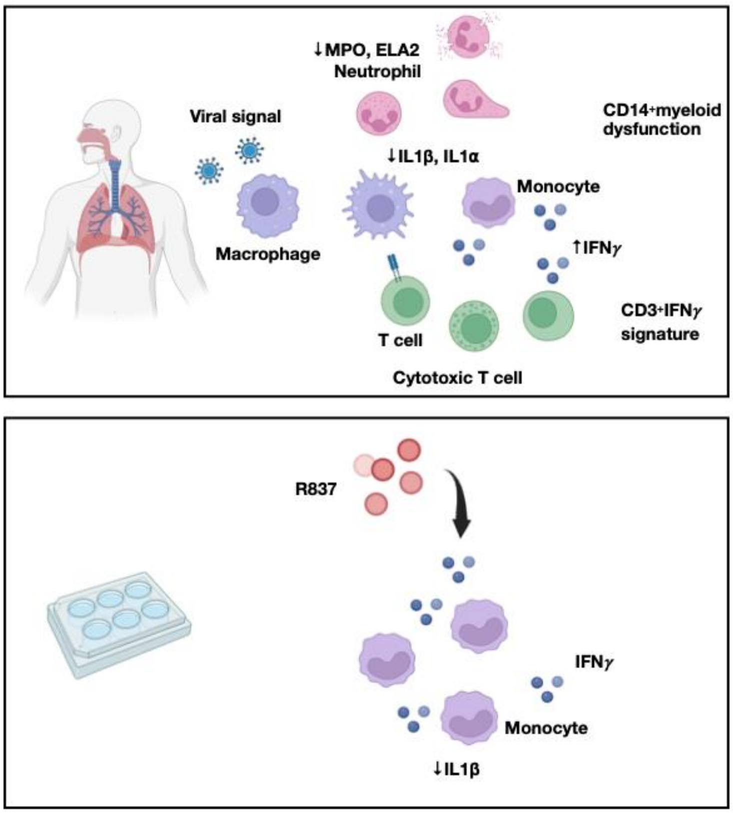

Ensure all supplementary figures referenced (e.g., S1B, S3, S4) are consistently cited and their relevance to key points is clearly articulated.

We have checked and ensured that supplemental figures are cited correctly.

The acronym "IFNg" should be replaced with “IFN-γ” throughout for clarity and formatting consistency with immunology standards.

We have checked and replaced all with IFN-γ.

References:

1. Morrell ED, Bhatraju PK, Mikacenic CR *et al*. Alveolar Macrophage Transcriptional Programs Are Associated with Outcomes in Acute Respiratory Distress Syndrome. American journal of respiratory and critical care medicine 2019; 200(6):732-741. 10.1164/rccm.201807-1381OC
2. Khemani RG, Smith L, Lopez-Fernandez YM *et al*. Paediatric acute respiratory distress syndrome incidence and epidemiology (PARDIE): an international, observational study. The Lancet Respiratory medicine 2019; 7(2):115-128. 10.1016/s2213-2600(18)30344-8
3. Wong JJ, Phan HP, Phumeetham S *et al*. Risk Stratification in Pediatric Acute Respiratory Distress Syndrome: A Multicenter Observational Study. Critical care medicine 2017; 45(11):1820-1828. 10.1097/ccm.0000000000002623
4. Song R, Gao Y, Dozmorov I *et al*. IRF1 governs the differential interferon-stimulated gene responses in human monocytes and macrophages by regulating chromatin accessibility. Cell Reports 2021; 34(12):108891. https://doi.org/10.1016/j.celrep.2021.108891
5. Loske J, Röhmel J, Lukassen S *et al*. Pre-activated antiviral innate immunity in the upper airways controls early SARS-CoV-2 infection in children. Nature Biotechnology 2022; 40(3):319-324. 10.1038/s41587-021-01037-9
6. Grant RA, Morales-Nebreda L, Markov NS *et al*. Circuits between infected macrophages and T cells in SARS-CoV-2 pneumonia. Nature 2021; 590(7847):635-641. 10.1038/s41586-020-03148-w
7. Flerlage T, Crawford JC, Allen EK *et al*. Single cell transcriptomics identifies distinct profiles in pediatric acute respiratory distress syndrome. Nature communications 2023; 14(1):3870. 10.1038/s41467-023-39593-0
8. Qiao Y, Giannopoulou Eugenia G, Chan Chun H *et al*. Synergistic Activation of Inflammatory Cytokine Genes by Interferon-γ-Induced Chromatin Remodeling and Toll-like Receptor Signaling. Immunity 2013; 39(3):454-469. https://doi.org/10.1016/j.immuni.2013.08.009
9. Masters S, Mielke L, Cornish A *et al*. Regulation of interleukin-1β by interferon-γ is species specific, limited by suppressor of cytokine signalling 1 and influences interleukin-17 production. EMBO reports 2010; 11:640-646. 10.1038/embor.2010.93
10. Chen J, Ivashkiv LB. IFN-γ abrogates endotoxin tolerance by facilitating Toll-like receptor-induced chromatin remodeling. Proceedings of the National Academy of Sciences 2010; 107(45):19438-19443. doi:10.1073/pnas.1007816107
11. Ornatowska M, Azim AC, Wang X *et al*. Functional genomics of silencing TREM-1 on TLR4 signaling in macrophages. American journal of physiology Lung cellular and molecular physiology 2007; 293(6):L1377-1384. 10.1152/ajplung.00140.2007
12. Bleharski JR, Kiessler V, Buonsanti C *et al*. A Role for Triggering Receptor Expressed on Myeloid Cells-1 in Host Defense During the Early-Induced and Adaptive Phases of the Immune Response1. The Journal of Immunology 2003; 170(7):3812-3818. 10.4049/jimmunol.170.7.3812
13. Zheng H, Heiderscheidt CA, Joo M *et al*. MYD88-dependent and -independent activation of TREM-1 via specific TLR ligands. European Journal of Immunology 2010; 40(1):162-171. https://doi.org/10.1002/eji.200839156
14. Ramirez-Ortiz ZG, Prasad A, Griffith JW *et al*. The receptor TREML4 amplifies TLR7-mediated signaling during antiviral responses and autoimmunity. Nat Immunol 2015; 16(5):495-504. 10.1038/ni.3143

Title: A High Dimensionality Approach Reveals Immunopathogenic Responses Driving Severe Pediatric Acute Respiratory Distress Syndrome

Manuscript no.: NCOMMS-25-32018A

Reviewer #1 (Remarks to the Author):

Thank you for answering the questions and revising the manuscript. In my opinion, some of the biological relationships described have not been sufficiently investigated to support their current interpretation. I would suggest more cautious wording, as the data in some parts do not yet provide robust evidence of pathophysiologically relevant changes.

We concur with the reviewer's recommendation for a cautious interpretation. Accordingly, we have used cautious language throughout the manuscript indicating that findings were associative and merely indicate a possible pathogenic immune mechanism. By highlighting the need for future work throughout the Discussion section, we hope to also convey the need for further robust pathophysiological evidence.

Given the relatively small group size, a normal distribution, which is assumed in both the Student's t-test and the Pearson correlation, seems unlikely. It is advisable to consider nonparametric alternatives such as Mann-Whitney-U-Test and Spearman's rank correlation.

We thank the reviewer for this comment. The data distribution of respiratory analytes quantified by ELISA is illustrated below through histograms and Q-Q plots which demonstrate normality of transformed values. Accordingly, we have retained the use of parametric tests for this analysis. However, for FACS data expressed as percentage of CD45^{+} cells, we agree that the distribution is more suited to non-parametric analysis. We have amended the Figures 1, 2, 5 and S1 along with the figure legends accordingly.

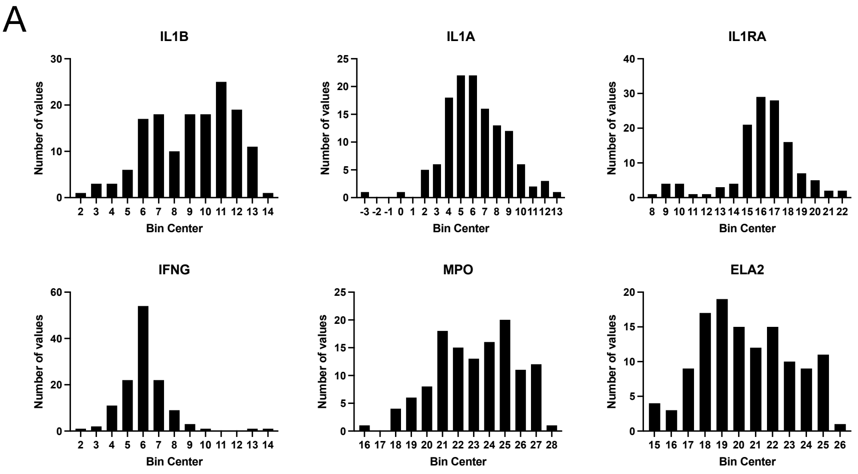

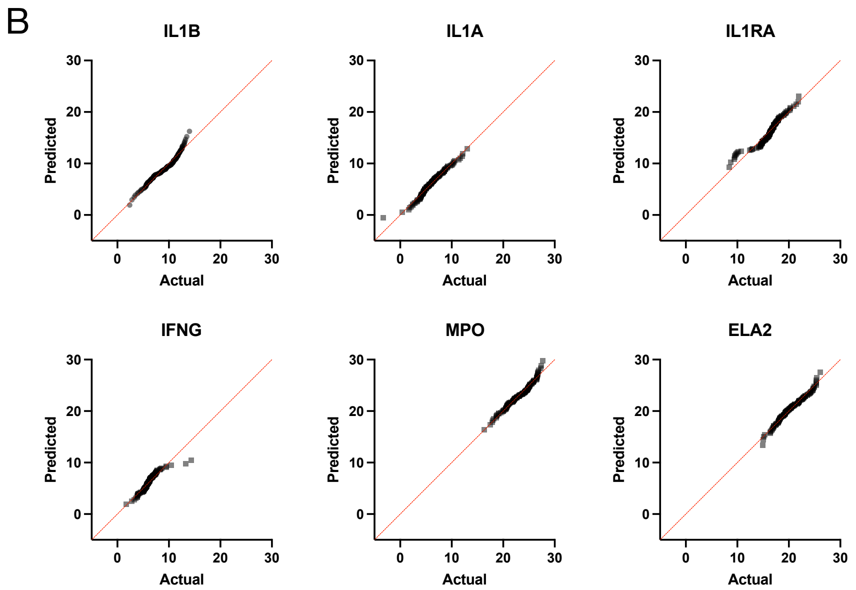

Histograms and Q-Q for each ELISA analyte

The analyses should also consistently include negative controls in order to draw valid conclusions from the data. For example Fig. 1B / Fig 2 B. In order to be able to assess any effect between the different patient groups, I believe it is essential to see whether there is any difference in gene regulation compared to the controls.

We thank the reviewer for this comment. We clarify that Figure 1B and 2B present *NanoString* differential expression (fold change) of respiratory T-cell and myeloid gene markers of non-severe and severe PARDS in comparison to control. This analysis incorporates a negative control cohort, as indicated in the respective figure legends, which also specify the number of patients included in each group.

It seems not sufficient to show only the percentages of the different cell types (e.g. Fig 1 and 2) in order to answer the question of which cell types are predominant in PARDS lungs. Reporting only percentages in FACS analysis can be misleading. Percentages, can be affected by changes in other populations, and may obscure biologically or clinically relevant differences. Absolute numbers are essential for accurate interpretation and meaningful comparisons between samples. Especially if this results in a key statement of the paper.

We thank the reviewer for this comment and the opportunity to clarify regarding the deep tracheal lavage (DTL) sampling technique. DTL specimens obtained from pediatric patients differ compared to protocolised bronchoalveolar lavage (BAL) performed for research in adult subjects. A key distinction lies in the variability of the return volume, which introduces differences in the dilution factor across samples. To account for this variability, we expressed cell subset data as a percentage of total CD45 cells within each lavage specimen. This approach was selected to better represent the

proportion of CD45 cells present in the alveolar lining fluid and to minimize the impact of sample dilution variability on the interpretation of results. In contrast, reporting absolute cell counts may be confounded by these variable dilution factors, potentially leading to inaccurate comparisons between samples. Therefore, percentages of total CD45 cells provide a more reliable metric for assessing cellular composition in pediatric DTL specimens. In the revision, we include an explanation in the Discussion section:

"And lastly, though BAL is considered the gold standard method of sampling of alveolar lining fluid, bronchoscopies are rarely performed in children. As such, we adopted the DTL method which is similar to a blind BAL. Though cellular composition of BAL and DTL was shown to be similar, there may be minor differences between the two sampling methods (55). A key distinction lies in the variability of the return volume, which introduces differences in the dilution factor across samples. To account for this variability, we expressed cell subset data as a percentage of total CD45 cells within each lavage specimen rather than absolute cell counts."

For reference, we have included the absolute counts of cell subsets below. These data indicate that the distribution of absolute counts across all subsets was similar and influenced by sample dilution and not reflective underlying biological factors.

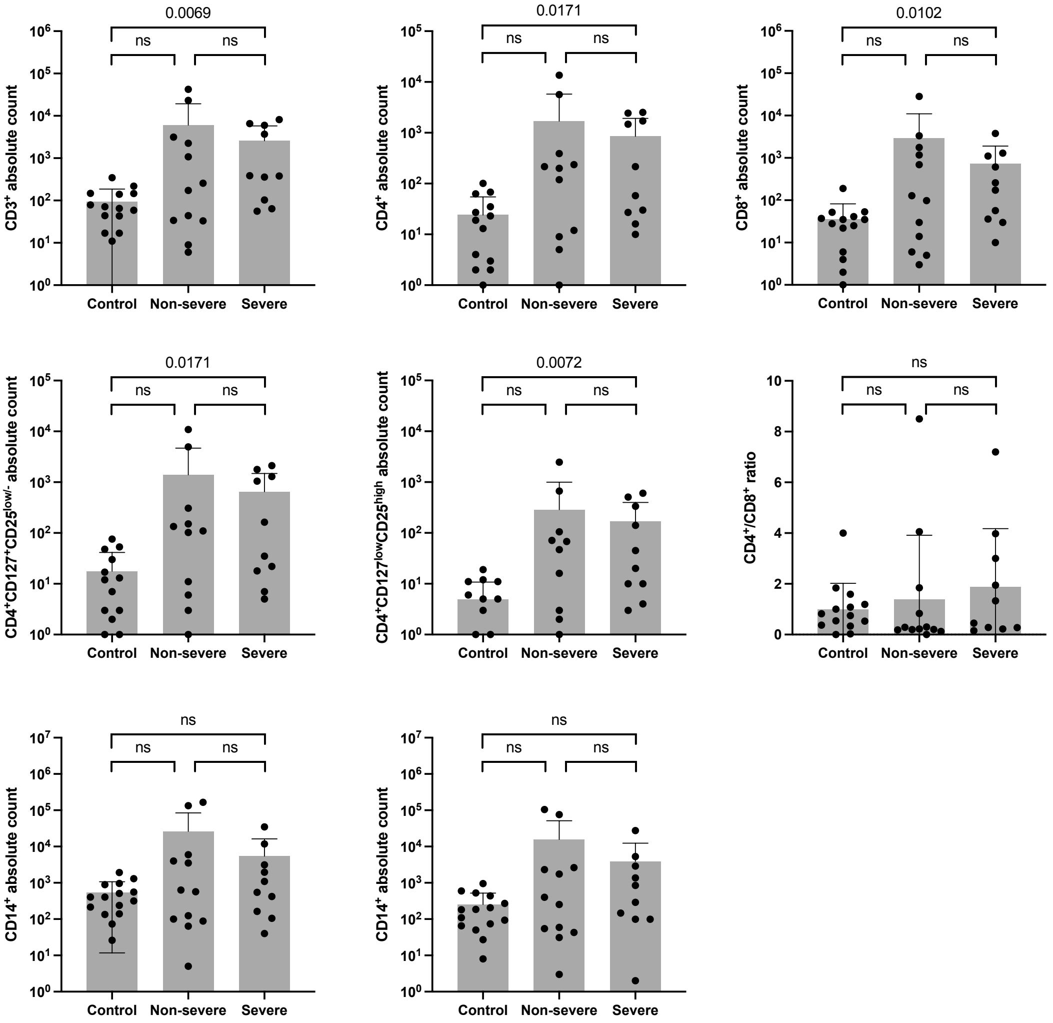

Although there may be a weak correlation between IFN-γ and the oxygenation index in Fig. 1, the question arises as to what biological relevance should be demonstrated here, since there is no difference in IFN-γ between controls, non-severe, and severe PARDS patients. Since IL-18 is mainly induced during viral infections and closely linked to IFN-γ signaling, including corresponding data would significantly strengthen the manuscript. As IL-18 activity depends on post-translational processing rather than transcriptional regulation, RNA-based analyses alone are insufficient to accurately assess its functional production.

We thank the reviewer for this insightful comment. In the revision, we have added data of IL-18 protein production and a discussion on its potential role. IL-18 production did not correlate with disease severity nor was it increased in severe PARDS vs. non-severe PARDS or controls. We have added the following sentences to the Results and Discussion section:

"To understand if these cytokine changes were related to the inflammasome pathway, we evaluated IL-18 as a corroborative marker of inflammasome activation however it had no relationship with PARDS severity (Figure S2)."

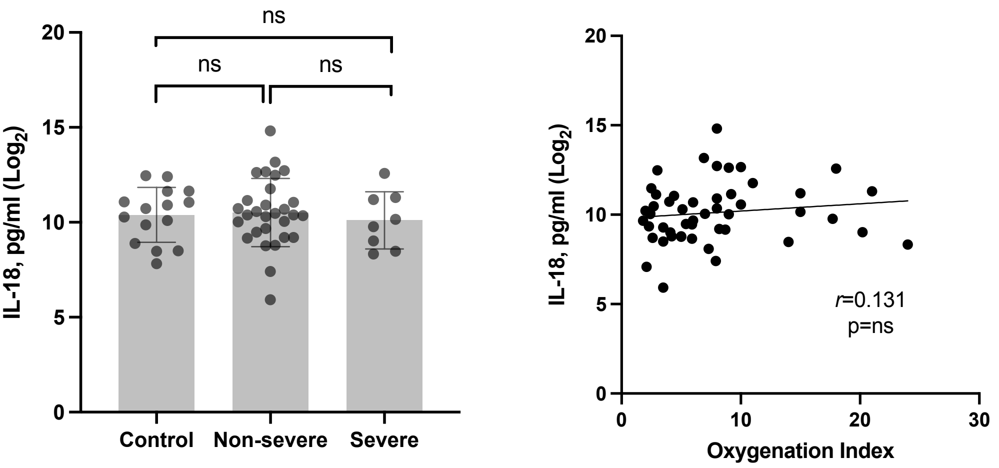

Figure S2A: IL-18 concentration

A.IL-18 concentration in the deep tracheal lavage fluid and its correlation (Pearson's) with the oxygenation index. Student's t-test was used for two group comparisons of IL-18 concentration between severe PARDS vs. control, non-severe PARDS vs. control and non-severe vs. severe PARDS.

""IL-1 production is closely associated with inflammasome activation, although inflammasome-independent IL-1 production has also been documented (43). Our study found that IL-18, an inflammasome-related cytokine and IFN-γ inducer, did not show a correlation with disease severity in PARDS patients, and inflammasome genes were not differentially expressed in the *in vitro* model. These findings indicate that the observed changes are possibly independent of inflammasome activity."

<u>Title: A High Dimensionality Approach Reveals Immunopathogenic Responses Driving Severe Pediatric Acute Respiratory Distress Syndrome</u>

<u>Manuscript no.: NCOMMS-25-32018B</u>

We thank the editor and reviewers for their comments and feedback, and the opportunity to provide further revision.

Reviewer #1 (Remarks to the Author):

While I agree that the T cell subsets are increased in subsets in patients with PARDS as compared to age-matched controls (S1), Fig. 1c does not demonstrate a positive association with disease severity, as no differences are observed between severe and non-severe cases across any of the cell types shown. Please explain what you mean with: “This influx of T-cell subsets was positively associated with disease severity (Figure 1C).”

We thank the reviewer for identifying this error and we have amended this sentence to:

“There was a significant increase of all pulmonary T-cell subsets in patients with PARDS as compared to age-matched controls (Figure S1) though this influx of T-cell subsets was not significantly associated with disease severity (Figure 1C).”

You state that quantitatively, the proportion of pulmonary CD14+ cells was significantly reduced in DTL samples from PARDS vs controls (Figure S1), in particular non-severe PARDS vs. controls. In Fig. 2C (CD14+ cells), this effect is observed only in non-severe PARDS; moreover, there is no significant difference between non-severe and severe cases, and no statistical difference is detected for $CD33^{+}$ cells. Given this, it is unclear why Fig. 1 and Fig. 2 differentiates between severe and non-severe PARDS. Please explain why certain effects are observed exclusively in non-severe PARDS, while others are evident only in severe PARDS. For me this appears highly inconsistent and may be driven by random variation rather than indicating a correlation with disease severity in either Fig. 1 or Fig. 2.

We thank the reviewer for the opportunity to clarify the rationale for stratifying data by PARDS severity. The decision to analyze data according to PARDS severity is grounded in clinical observations and published evidence. Severe forms of ARDS and PARDS are known to carry a substantially higher risk of adverse outcomes, including increased mortality[1, 2]. Additionally, patients with severe disease often experience different responses to therapeutic interventions compared to those with non-severe presentations [3, 4]. These distinctions suggest that the underlying immunological dysfunction in severe ARDS/PARDS may differ meaningfully from that observed in less severe cases. Therefore, it is important to characterize the immunological features of severe disease separately, to better understand the mechanisms involved and to potentially inform more targeted approaches to treatment.

We observed a quantitative reduction of CD14+ pulmonary myeloid cells in PARDS vs. controls. However the lack statistical difference between the severe PARDS vs. control group may be due to an inadequate sample size. Importantly, there was a qualitative/ functional difference in the myeloid cells from severe PARDS patients which was evident in the transcriptomic and proteomic data (IL1B, IL1A, MPO, ELA2).

In Fig. 3, you report that alveolar myeloid cells are the most abundant cell population. This heterogeneous subset includes innate immune cells and likely comprises $CD14^+$ cells (also shown in Fig.3 C), which you previously report to be reduced. Please clarify how these findings can be reconciled.

We thank the reviewer for this comment. In the single-cell RNA sequencing data, alveolar myeloid cells were the most abundant overall cell type constituting 55% of the cell population (40% expressing CD14; clusters 0, 1, 3, 7, 10) followed by CD3 expressing T cell, 23%. In the flow cytometry data which examines limited myeloid markers, the CD14 expressing cells were also the most abundant accounting for 30% of total CD45+ immune cells, followed by CD3 expressing T cells, 26%. The minor discrepancies between the percentages may be due to the number of patients with PARDS vs. controls in each experiment as well as technical differences between single-cell RNA sequencing and flow cytometry.

Figure 6 is somewhat confusing. Were all cells treated with ATP as signal 2 for inflammasome activation, as indicated in the graphical abstract? This treatment is not shown in the corresponding graphs. In addition, could you provide data on cell viability? Since TLR7-mediated IL-1β production is caspase-1 dependent and therefore activates pyroptosis. The observed reduction in cytokine production, depending on the experimental setup and prior IFN-γ priming, could also be explained by increased cell death rather than reduced cytokine production. In any case, the proposed mechanism is not novel, as it was already described by Guarda et al. in Immunity (2011), and therefore adds limited new insight to the field.

We thank the reviewer for the opportunity to clarify the Figure 6 and regarding the novelty of this work.

Regarding the Figure 6 – reagents for the *in vitro* cell culture in *grey* [Poly(I:C), Poly(I:C) HMW, Poly(I:C) LMW and ATP] were tested but did not produce any IL-1β response – these were not reported. In the revision, we have added this explanation in the figure legend.

"Figure 6 *In vitro* human PBMC model suggesting the mechanism of IL1 suppression in severe PARDS

A. Schematic of the *in vitro* experimental protocol. Results utilizing reagents in *black* for the *in vitro* cell culture were reported. Reagents in *grey* [Poly(I:C), Poly(I:C) HMW, Poly(I:C) LMW and ATP] were tested but did not produce measurable IL-1β response – these were not presented."

Regarding cell viability, in the flow cytometry analysis in which dead cells were already gated out, IL-1β expression was increased in *live* monocytes upon R837 stimulation – expression of IL-1β in *live* monocytes were then shown to be decreased when IFN-γ priming was used. We have included a representative plot of data from flow cytometry of live $CD14^+$ monocyte singlet cells (Figure S9A). We also provide to the reviewer data

on the percentage of live monocytes in the culture condition with and without R837 which did not indicate significant cell death upon R837 activation (below).

Lastly, with regards to novelty of the proposed mechanism, we would like to highlight that the data presented by Guarda et al. was conducted mainly in murine bone marrow derived macrophages. Inflammasome activation was initiated by LPS (primary signal via TLR4) followed by alum (second signal via P2RX7) – under these conditions, type I interferon priming (but not IFN-γ) inhibited inflammasome activity and IL-1β production. They went on to show the same observation in primary human monocytes. In contrast, we show that TLR7 activation (instead of TLR4 and in the absence of a second signal) induced IL-1β production in primary human monocytes, which was inhibited by IFN-γ priming. Though we were not able to delineate the exact mechanism for IL-1β suppression, we postulate that activation/inhibition of the inflammasome pathway was not involved. Data to support this postulation was the lack of cleaved caspase 1 and IL-1β products on Western blot as well as lack of differential regulation of inflammasome genes on bulk RNA sequencing.

A detailed discussion of these points is found in paragraph 4 and 6 of the Discussion section:

"IL-1 production is closely associated with inflammasome activation, although inflammasome-independent IL-1 production has also been documented [5]. Our study found that IL-18, an inflammasome-related cytokine and IFN-γ inducer, did not show a correlation with disease severity in PARDS patients, and inflammasome genes were not differentially expressed in the *in vitro* model. These results suggest that the observed changes in IL-1 are likely independent of inflammasome activity."

"The suppressive effect of IFN-γ on IL-1β production has been reported before albeit under different conditions. In murine models, TLR4 stimulation of bone marrow derived macrophages describe that IFN-γ priming inhibits the p65 region of NFκB from binding to the IL-1β promoter region which directly reduced *IL1B* mRNA expression [6, 7]. It was suggested that an epigenomic mechanism was responsible for this effect – H3K9 and H3K14 acetylation on the *IL1B* promoter region is induced by LPS, but when acetylation was inhibited by IFN-γ priming, chromatin accessibility was reduced and *IL1B* transcription reduced [7]. Separately, type I interferon priming was shown to inhibit inflammasome activation through LPS induced IL-10 via STAT3-dependent mechanism [8]. However, we showed that IFN-γ priming instead enhanced TLR4-induced IL-1β production in human monocytes. Indeed, the effect of IFN-γ priming on TLR4 activation may be species specific [9-11] and the described epigenetic mechanism may not be preserved in humans. Other potential mechanisms include the IL1 antagonizing effect of IL-10 [12] and IL-1RA [13], however, both were not raised in PARDS patients or in the *in vitro* cell model and therefore, cannot account for our observations. Further studies are thus required to determine the mechanism involved in the suppressive effect of IFN-γ on IL-1β production in PARDS."

Figure S9

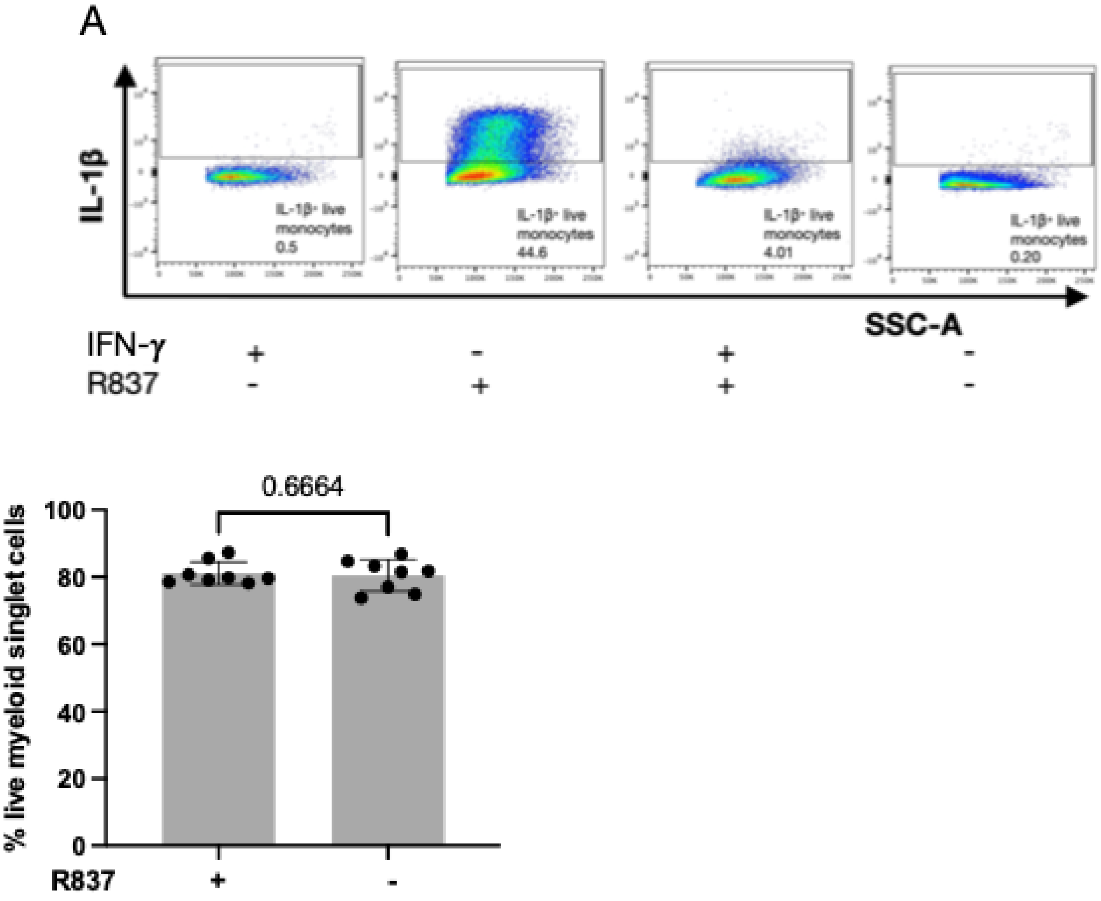

After evaluation, I feel that, while the study is carefully conducted, the presented data do not provide a substantial advance for the field. Identification of subphenotypes with clear clinical relevance would ideally require data from multiple centers. Although the identified cell clusters appear plausible, their biological significance remains unclear in several instances, and any association with disease severity is only weakly supported. Additionally, the mechanistic link demonstrated in cell culture, while interesting, is in my opinion established in the literature, albeit not previously shown specifically in the context of PARDS.

We thank the reviewer for this comment and fully agree that independent validation of our findings would be highly valuable. Such validation is essential to strengthen the robustness and generalisability of our results. As noted by the editor, our work is the first to demonstrate the interaction between IFN-γ and IL-1β, via TLR7 activation in the context of PARDS. Importantly, our findings suggest that this mechanism may play a role in the pathogenesis of this severe clinical phenotype. We believe that highlighting this context-specific involvement is a key contribution of our study and sets the stage for future multicentre investigations to further clarify its biological and clinical significance.

References

1. Wong JJ, Jit M, Sultana R *et al*. Mortality in Pediatric Acute Respiratory Distress Syndrome: A Systematic Review and Meta-Analysis. Journal of intensive care medicine 2017:885066617705109. 10.1177/0885066617705109
2. Bellani G, Laffey JG, Pham T *et al*. Epidemiology, Patterns of Care, and Mortality for Patients With Acute Respiratory Distress Syndrome in Intensive Care Units in 50 Countries. JAMA 2016; 315(8):788-800. 10.1001/jama.2016.0291
3. Villar J, Ferrando C, Martínez D *et al*. Dexamethasone treatment for the acute respiratory distress syndrome: a multicentre, randomised controlled trial. The Lancet Respiratory medicine 2020; 8(3):267-276. 10.1016/s2213-2600(19)30417-5
4. Moreno G, Carbonell R, Martin-Loeches I *et al*. Corticosteroid treatment and mortality in mechanically ventilated COVID-19-associated acute respiratory distress syndrome (ARDS) patients: a multicentre cohort study. Ann Intensive Care 2021; 11(1):159. 10.1186/s13613-021-00951-0
5. Netea MG, van de Veerdonk FL, van der Meer JW *et al*. Inflammasome-independent regulation of IL-1-family cytokines. Annu Rev Immunol 2015; 33:49-77. 10.1146/annurev-immunol-032414-112306
6. Eigenbrod T, Bode KA, Dalpke AH. Early inhibition of IL-1β expression by IFN-γ is mediated by impaired binding of NF-κB to the IL-1β promoter but is independent of nitric oxide. J Immunol 2013; 190(12):6533-6541. 10.4049/jimmunol.1300324
7. Hoeksema MA, Scicluna BP, Boshuizen MC *et al*. IFN-γ priming of macrophages represses a part of the inflammatory program and attenuates neutrophil recruitment. Journal of immunology (Baltimore, Md : 1950) 2015; 194(8):3909-3916. 10.4049/jimmunol.1402077
8. Guarda G, Braun M, Staehli F *et al*. Type I interferon inhibits interleukin-1 production and inflammasome activation. Immunity 2011; 34(2):213-223. 10.1016/j.immuni.2011.02.006
9. Qiao Y, Giannopoulou Eugenia G, Chan Chun H *et al*. Synergistic Activation of Inflammatory Cytokine Genes by Interferon-γ-Induced Chromatin Remodeling and Toll-like Receptor Signaling. Immunity 2013; 39(3):454-469. https://doi.org/10.1016/j.immuni.2013.08.009
10. Masters S, Mielke L, Cornish A *et al*. Regulation of interleukin-1β by interferon-γ is species specific, limited by suppressor of cytokine signalling 1 and influences interleukin-17 production. EMBO reports 2010; 11:640-646. 10.1038/embor.2010.93
11. Chen J, Ivashkiv LB. IFN-γ abrogates endotoxin tolerance by facilitating Toll-like receptor-induced chromatin remodeling. Proceedings of the National Academy of Sciences 2010; 107(45):19438-19443. doi:10.1073/pnas.1007816107
12. Sun Y, Ma J, Li D *et al*. Interleukin-10 inhibits interleukin-1β production and inflammasome activation of microglia in epileptic seizures. Journal of Neuroinflammation 2019; 16(1):66. 10.1186/s12974-019-1452-1
13. Mayer-Barber KD, Andrade BB, Barber DL *et al*. Innate and adaptive interferons suppress IL-1α and IL-1β production by distinct pulmonary myeloid subsets during Mycobacterium tuberculosis infection. Immunity 2011; 35(6):1023-1034. 10.1016/j.immuni.2011.12.002
